# Randomized Evaluation of the Effects of Anacetrapib through Lipid-modification (REVEAL)—A large-scale, randomized, placebo-controlled trial of the clinical effects of anacetrapib among people with established vascular disease: Trial design, recruitment, and baseline characteristics

**DOI:** 10.1016/j.ahj.2017.02.021

**Published:** 2017-05

**Authors:** M.J. Landray, REVEAL Collaborative Group, L. Bowman, F. Chen, E. Sammons, J.C. Hopewell, K. Wallendszus, W. Stevens, E. Valdes-Marquez, S. Wiviott, C.P. Cannon, E. Braunwald, R. Collins, M.J. Landray (Writing Committtee)

**Affiliations:** REVEAL Central Coordinating Office, Clinical Trial Service Unit and Epidemiological Studies Unit, Nuffield Department of Population Health, University of Oxford, United Kingdom

## Abstract

Patients with prior vascular disease remain at high risk for cardiovascular events despite intensive statin–based treatment. Inhibition of cholesteryl ester transfer protein by anacetrapib reduces low-density lipoprotein (LDL) cholesterol by around 25% to 40% and more than doubles high-density lipoprotein (HDL) cholesterol. However, it is not known if these apparently favorable lipid changes translate into reductions in cardiovascular events.

**Methods:**

The REVEAL study is a randomized, double-blind, placebo-controlled clinical trial that is assessing the efficacy and safety of adding anacetrapib to effective LDL-lowering treatment with atorvastatin for an average of at least 4 years among patients with preexisting atherosclerotic vascular disease. The primary assessment is an intention-to-treat comparison among all randomized participants of the effects of allocation to anacetrapib on major coronary events (defined as the occurrence of coronary death, myocardial infarction, or coronary revascularization).

**Results:**

Between August 2011 and October 2013, 30,449 individuals in Europe, North America, and China were randomized to receive anacetrapib 100 mg daily or matching placebo. Mean (SD) age was 67 (8) years, 84% were male, 88% had a history of coronary heart disease, 22% had cerebrovascular disease, and 37% had diabetes mellitus. At the randomization visit (after at least 8 weeks on a protocol-defined atorvastatin regimen), mean plasma LDL cholesterol was 61 (15) mg/dL and HDL cholesterol was 40 (10) mg/dL.

**Interpretation:**

The REVEAL trial will provide a robust evaluation of the clinical efficacy and safety of adding anacetrapib to an effective statin regimen. Results are anticipated in 2017.

## Background

Large-scale randomized trials have shown that lowering low-density lipoprotein (LDL) cholesterol concentration by about 40 mg/dL (1 mmol/L) for 4 to 5 years reduces the risk of coronary events (including myocardial infarction [MI], coronary death, and revascularization procedures) and stroke by about one-fifth,^1^ and that lowering LDL cholesterol more (either with more effective statin regimens or with the addition of ezetimibe) produces further reductions in risk.2., 3. Nevertheless, among individuals who already have occlusive vascular disease, the risk of cardiovascular events remains high.2., 3.

Although higher high-density lipoprotein (HDL) cholesterol concentration is associated with lower risk of coronary events in observational epidemiologic studies,4., 5. there is little evidence that treatments that raise HDL cholesterol reduce the risk of vascular events. Trials using fibrates (which raise HDL cholesterol by about 5%-10%) have produced mixed results,^6^ whereas adding niacin (which increases HDL cholesterol by 14%-20%) to a statin had no effect on the risk of cardiovascular events but was associated with significant hazards (including serious adverse events [SAEs] related to the gastrointestinal and musculoskeletal systems, skin, diabetes, infection, and bleeding).7., 8.

Cholesteryl ester transfer protein (CETP) facilitates exchange of cholesteryl esters and triglycerides between HDL particles and atherogenic apolipoprotein B–containing particles in the plasma. Genetic variants that lead to lower CETP activity are associated with higher HDL cholesterol levels and lower risk of coronary heart disease9., 10. (by contrast with other genetic variants that impact HDL cholesterol levels).^11^ Pharmacological CETP inhibition produces increases in HDL cholesterol and apolipoprotein A1, along with reductions in LDL cholesterol and apolipoprotein B.^12^ The magnitude of these effects varies between different CETP inhibitors. Torcetrapib was the first CETP inhibitor to be studied in a large-scale outcome trial. However, the ILLUMINATE trial in 15,000 patients with preexisting cardiovascular disease was stopped prematurely in 2007 because of an excess risk of death among patients allocated to receive torcetrapib.^13^ Torcetrapib was found to increase systolic blood pressure by about 5 mm Hg as well as causing increases in blood sodium, bicarbonate, and aldosterone concentrations.13., 14., 15.

Cardiovascular outcome trials with 2 other CETP inhibitors were also terminated early because of a lack of efficacy. In 2012, the Dal-OUTCOMES trial of dalcetrapib in 16,000 patients with a recent acute coronary syndrome was stopped prematurely because of a lack of clinically meaningful reductions in cardiovascular events but with no significant safety concerns.^16^ Dalcetrapib has only modest effects on blood lipids, increasing HDL cholesterol by about 30% with no effect on LDL cholesterol, and also increasing systolic blood pressure by 0.6 mm Hg in Dal-OUTCOMES. In 2015, the ACCELERATE trial of evacetrapib, which doubles HDL cholesterol and lowers LDL cholesterol by about one quarter,17., 18. was stopped early because of insufficient evidence of efficacy.19., 20. There was an increased risk of investigator-reported hypertension and a 1 mm Hg increase in systolic blood pressure.

Anacetrapib is an orally active CETP inhibitor that was well tolerated in early-phase studies. In the placebo-controlled DEFINE trial among 1,600 patients followed up for 18 months, anacetrapib 100 mg daily produced substantial increases in HDL cholesterol and apolipoprotein A1 (138% and 45%, respectively) and reductions in LDL cholesterol, apolipoprotein B, and lipoprotein(a) (40%, 21%, and 36%, respectively).^21^ However, subsequent investigations suggest that the reduction in LDL cholesterol may be somewhat smaller (around 25%) if measured by β-quantification rather than direct methods.^22^ There have been no discernible effects on resting or ambulatory blood pressure or on plasma aldosterone concentrations.^23^

The REVEAL trial was designed to assess the clinical efficacy and safety of anacetrapib 100 mg once daily among patients with preexisting atherosclerotic vascular disease who are also receiving effective LDL-lowering therapy using atorvastatin. In this article, we present the REVEAL trial design, prerandomization recruitment and run-in period, baseline participant characteristics, and data analysis plan.

## Methods

### Aims

**Figure 1:**
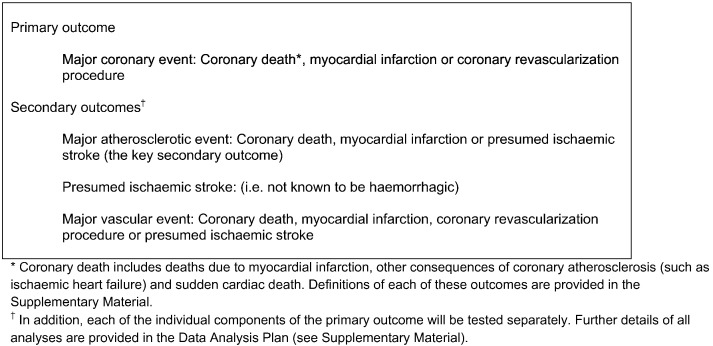
Primary and secondary outcomes.

The REVEAL trial aimed to randomize at least 30,000 participants 50 years or older with preexisting atherosclerotic vascular disease between anacetrapib 100 mg daily and matching placebo for a median of at least 4 years (Supplementary Figure 1). All participants were to receive effective LDL-lowering treatment using atorvastatin. The primary outcome for the assessment of efficacy is *major coronary event*, which is defined as coronary death, MI, or coronary revascularization (Figure 1).

### Study organization

The study was designed by the independent investigators at the Clinical Trial Service Unit at the University of Oxford (the regulatory trial sponsor) in collaboration with the TIMI Study Group based at Brigham and Women's Hospital, Boston, and Merck & Co, Inc (Merck), which manufactures anacetrapib and provides funding for the study. The independent Steering Committee is responsible for drafting the main reports from the study. Although Merck has the opportunity to comment on drafts of manuscripts, it otherwise has no role in the analysis of the data presented, the preparation and approval of this manuscript, or the decision to submit it for publication. The study protocol (see Supplementary Material) was approved by the relevant institutional review board and drug regulatory authority for each participating center.

### Eligibility

**Figure 2:**
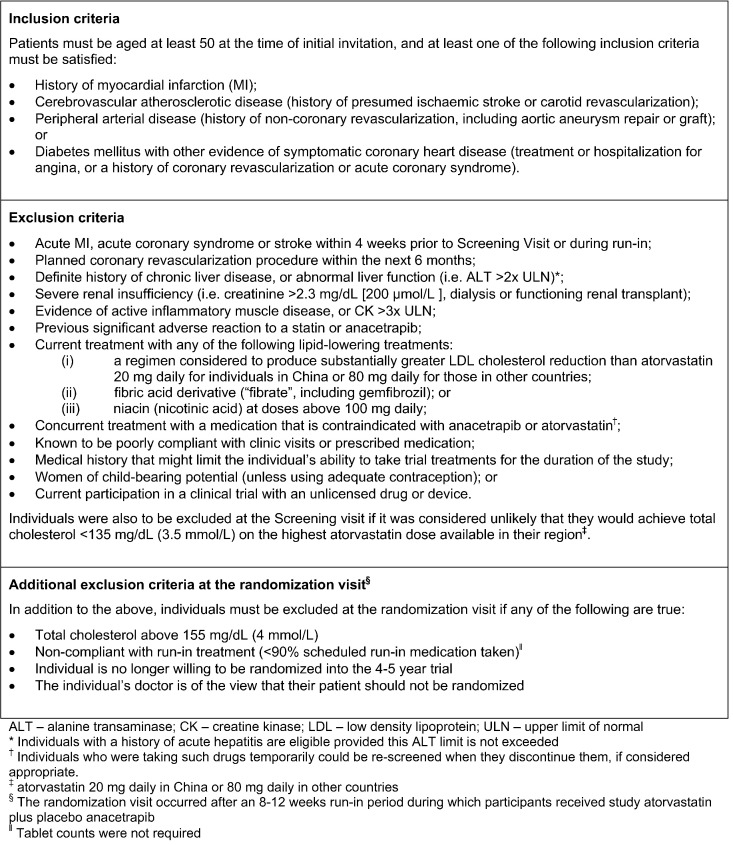
Inclusion and exclusion criteria for entry into the REVEAL trial.

The REVEAL trial aimed to enroll a broad range of participants at high risk for future cardiovascular events (see Figure 2). Men and women older than 50 years were considered eligible if they met at least one of the inclusion criteria (history of MI, cerebrovascular atherosclerotic disease, peripheral arterial disease, or diabetes mellitus with other evidence of symptomatic coronary heart disease), had none of the exclusion criteria, and if their own doctor did not consider there to be a definite contraindication to either anacetrapib or atorvastatin (the protocol-specified background statin treatment for all participants).

### Invitation, screening, and run-in phase

Extensive prescreening efforts were made to identify large numbers of potential participants at each recruiting site. Potentially eligible patients were identified from clinical records (including electronic health care records) and, in many places, were contacted to seek their provisional agreement to attend a screening visit. The exact methods varied by center and by country, and in all cases were subject to appropriate institutional review board approval and compliance with data privacy regulations. Potential participants were given information about the study and invited to attend a screening visit.

At the screening visit, relevant medical history, current medication, and other factors pertinent to eligibility were recorded directly on to the screening visit form on the study IT system (which was designed to ensure that complete information was obtained and to prompt actions in line with the protocol). Potentially eligible individuals had the study explained to them by the clinic staff and were asked to provide written informed consent. In addition, they were asked to indicate on the consent form which, if any, of the samples of plasma, serum, cells, or urine could be stored long term for future unspecified research analyses. Blood pressure was measured and a non–fasting blood sample was taken for immediate measurement of total cholesterol, alanine transaminase (ALT), creatine kinase (CK), and creatinine using a Reflotron Plus (Roche Diagnostics International, Rotkreuz, Switzerland) dry chemistry analyzer. These results were recorded directly on the study clinic IT system, which provided an assessment of eligibility and determined the atorvastatin dose to be used for that individual (see below). At the end of the screening visit, eligible participants were issued with a 12-week supply of active atorvastatin (at an appropriate dose) and placebo anacetrapib, with 1 tablet of each to be taken daily. All other lipid-modifying treatments were stopped. An appointment was made for a randomization visit in 8 to 12 weeks.

The prerandomization, single-blind, run-in phase was intended to help identify and exclude before randomization those individuals who would be unlikely to comply with long-term study treatment and follow-up. During run-in, the local investigator (and, where appropriate, each individual's primary care physician or hospital specialist) was given a description of their patient's medical history, previous lipid-modifying treatment and screening visit total cholesterol result, and advised of the atorvastatin dose that would be provided during the trial. The doctor was asked to indicate whether, in their view, their patient was unsuitable for entry into the randomized phase of the study (for whatever reason). The run-in period also provided screened participants with the opportunity to consider whether they remained willing to take part in the randomized comparison, take study medication, and attend follow-up visits for at least 4 to 5 years.

### Determination of atorvastatin dose

All participants who entered the run-in phase were issued with an atorvastatin regimen that was intended to reduce their LDL cholesterol to less than 77 mg/dL (2 mmol/L) and which was at least as intensive as their current LDL-lowering treatment (see Supplementary Material). At the screening visit, a computerized algorithm used the dry chemistry total cholesterol measurement and the type and dose of their current (prestudy) LDL-lowering therapy (statin and/or ezetimibe) to determine eligibility and the minimum dose of study atorvastatin (either 10 or 20 mg in China, 20 or 80 mg in other countries) likely to be required to lower total cholesterol to less than 135 mg/dL (3.5 mmol/L). Individuals were excluded if they were already receiving LDL-lowering treatment that was more intensive than the maximum atorvastatin dose available in the trial or if it was unlikely that they would achieve the target total cholesterol on that dose of atorvastatin.

### Randomization visit

At the randomization visit, eligibility was rechecked and information on SAEs and any other significant problems during the run-in period were recorded. Nonstudy medication, compliance with run-in treatment, and consent information were checked. Information about smoking history and alcohol intake was sought and an assessment of quality of life was made using the EQ5D questionnaire. Blood pressure (measured once with patient sitting using Omron BP760 (Omron Healthcare UK, Milton Keynes, UK) or equivalent local model), height, weight, and hip, waist, and neck circumference were measured. All details were recorded directly on the Randomization Form on the clinic IT system. A blood sample was taken for immediate dry chemistry measurement of total cholesterol, ALT, and CK. Individuals could only remain eligible if total cholesterol was ≤155 mg/dL (4 mmol/L), ALT ≤2× upper limit of normal, and CK ≤3× upper limit of normal. Additional non–fasting blood samples and a urine sample were taken for central laboratory assays of lipid profile, glycosylated hemoglobin, plasma creatinine, and urinary albumin/creatinine ratio (see Supplementary Material). Samples of genetic material, plasma, serum, and urine are being stored long term for future analysis, subject to appropriate consents.

Eligible and consenting individuals were allocated anacetrapib or placebo using a minimized randomization program embedded within the clinic IT system that helps maximize balance between the treatment groups with respect to prognostically important variables (see Supplementary Material).^24^ Randomized participants were issued with a 7-month supply of study treatment consisting of (*a*) anacetrapib 100 mg or matching placebo, and (*b*) active atorvastatin (at the same dose issued at the screening visit). An appointment for the first postrandomization follow-up visit was made and the participant's doctor(s) was notified that his/her patient had been randomized.

### Post‐randomization follow-up

After randomization, it is intended that all participants remain on study treatment and that follow-up information is collected for the full duration of the study. Clinic follow-up visits are scheduled at 2 and 6 months initially (with an additional visit at 4 months in China, at the request of the Chinese Food and Drug Administration), and then 6 monthly until the end of the study. Additional “early recall” visits are arranged for any participants who require review outside their planned visit schedule. Details of the follow-up visit assessments are provided in the Supplementary Material. Wherever possible, extended follow-up (off study treatment) of all surviving randomized participants will continue for at least 2 years beyond the final study visit to provide information on the longer-term safety and efficacy of anacetrapib.

### Adverse event recording and adjudication of potential study outcomes

At each follow-up visit, study staff seek details of all SAEs (including study outcomes and reasons for hospitalization), any non-serious adverse events attributed to or resulting in discontinuation of study treatment, and any symptoms of muscle pain or weakness or suggestive of hepatitis (loss of appetite, nausea, jaundice, lethargy, or malaise). At the request of the US Food and Drug Administration, information on all other non-serious adverse events is sought for participants in North America. Potential study outcomes are adjudicated by clinicians based at or overseen by the Central Coordinating Office, blind to study treatment allocation or blood lipid values and using standard definitions (see Supplementary Material).

### Statistical considerations

#### Sample size and predicted number of events

Based on a major coronary event rate of 1.8% per annum and median follow-up of 4 years, a trial of 30,000 participants would have 88% power at 2-tailed *P* < .01 to detect a 15% relative risk reduction (see Supplementary Material). Follow-up of all randomized participants is planned to continue for a median of at least 4 years, and until at least 1900 participants have had an unrefuted major coronary event (primary end point) plus at least 950 participants have had a coronary death or MI.

#### Data analysis plan

All participants randomized to anacetrapib will be compared with all participants randomized to placebo, regardless of whether a participant received all, some or none of the allocated treatment (ie, intention-to-treat analyses). For each outcome, survival analytic methods will be used to evaluate the time to the first event during the entire study period. The log-rank method will be used to estimate the average event rate ratio comparing all those allocated active anacetrapib with all those allocated placebo. A 2-tailed *P* value <.05 will be considered statistically significant. Further details (including the approach to control for multiplicity) are provided in the protocol, Data Analysis Plan, and other online Supplementary Material, which were finalized by the trial Steering Committee blind to any information on the effect of study treatment on study outcomes.

#### Role of the independent Data Monitoring Committee

An independent Data Monitoring Committee (DMC) is responsible for reviewing interim unblinded analyses of the effect of randomized treatment allocation on all SAEs and other study outcomes. The DMC is to advise the Steering Committee if, in their view, any modification to the protocol is required. Such assessments are carried out at a frequency relevant to the stage of the study (typically at 6-12 monthly intervals with a Chairman's review every 3-6 months). Further details, including guidelines for once only consideration of stopping for benefit or futility at 3 years' median follow-up, are provided in the DMC Charter (see Supplementary Material).

## Results

### Screening and run-in

**Figure 3:**
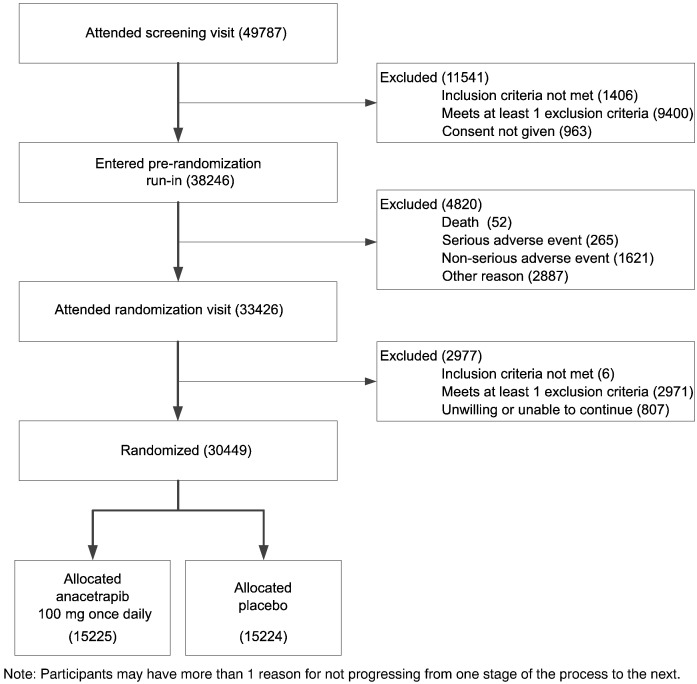
Trial profile — flow of participants through the REVEAL trial.

A total of 49,787 patients attended a screening visit (see Figure 3): 29,318 in Europe, 8,249 in North America, and 12,220 in China (see Supplementary Table I). Data from one additional site in the United States have been excluded from this report because of evidence of significant breaches of Good Clinical Practice and the study protocol (see Supplementary Material).

Of the 49,787 people screened, 11,541 (23.2%) were excluded at the screening visit, 4,820 (9.7%) withdrew during the run-in period, and 2,977 (6.0%) were not eligible at the randomization visit. Of those screened, 1,406 (2.8%) did not meet any of the inclusion criteria, 1,441 (2.9%) were excluded on the basis of medical history, 1,392 (2.8%) were excluded because of concerns about long-term compliance with study medication and/or attendance at clinic visits, and 963 (1.9%) did not give consent (see Supplementary Table II). A total of 1,550 (3.1%) were excluded on the basis of medication history, with the chief reasons being a participant report of previous adverse reaction to a statin (735 [1.5%]) and the use of an LDL-lowering treatment regimen (statin and/or ezetimibe) that was considered to be more potent than the maximum dose of study atorvastatin (621 [1.2%]). A total of 5,437 (10.9%) individuals were excluded on the basis of their blood assay results, with 4,814 (9.7%) being due to a total cholesterol result that, together with knowledge of their current LDL-lowering treatment, gave a predicted total cholesterol result that was unlikely to be less than 135 mg/dL (3.5 mmol/L) on the maximum dose of study atorvastatin for their region. Overall, of the 11,541 individuals who were excluded at the screening visit, 5,435 (47%) were excluded either because their current LDL-lowering treatment was considered to be more potent than the maximal dose of study atorvastatin available or because it was considered unlikely that their total cholesterol would be less than 135 mg/dL (3.5 mmol/L) on study atorvastatin.

Of the 38,246 individuals who entered the run-in phase, 4,820 (12.6%) withdrew before attending a randomization visit (see Supplementary Table III). A total of 52 (0.1%) died of causes unrelated to study medication, 265 (0.7%) withdrew because of a nonfatal SAE (of which 4 were believed to be caused by the study atorvastatin), and 1,621 (4.2%) withdrew because of a non-serious adverse event (with musculoskeletal and gastrointestinal disorders being the most common). A total of 2887 (7.5%) had other reasons for withdrawing from run-in, principally concerns about or difficulty taking tablets, difficulty in traveling or attending clinic, or advice from their doctor.

Of the 33,426 individuals who attended a randomization visit, 2,977 (8.9%) were not eligible for randomization (see Supplementary Table IV). Of these, 6 (0.0%) no longer met the inclusion criteria, 1,063 (3.2%) were excluded on the basis of medical history (including 1 reported serious adverse reaction to study atorvastatin), 448 (1.3%) were on a contraindicated medication or poorly compliant with the run-in treatment (atorvastatin plus placebo anacetrapib), there were concerns about long-term compliance with study medication in 580 (1.7%), and 807 (2.4%) were unwilling or unable to attend regular study visits. A total of 1,606 (4.8%) individuals were excluded on the basis of the blood results, including 1,097 (3.3%) with a total cholesterol result greater than 155 mg/dL (4.0 mmol/L).

### Baseline characteristics of randomized participants

Between August 2011 and October 2013, 30,449 people (61.2% of those screened) were randomized: 15,738 from Europe, 6,082 from North America, and 8,629 from China (see Table and Supplementary Table V). Mean (SD) age was 67 (8) years and 25,534 (84%) were men. Overall, 26,679 (88%) had a history of coronary heart disease, 6,781 (22%) had a history of cerebrovascular disease, 2,435 (8%) had a history of peripheral arterial disease, and 11,320 (37%) had diabetes mellitus. The mean total cholesterol was 132 (21) mg/dL, LDL cholesterol was 61 (15) mg/dL (4,297 [14%] had LDL cholesterol ≥77 mg/dL [2.0 mmol/L]; see Supplementary Table VI), HDL cholesterol was 40 (10) mg/dL, and median (inter-quartile range) plasma triglycerides were 124 (89-174) mg/dL.

### Emerging information on adherence to study treatment and event rate

At a median of 3 years of follow-up, the overall adherence to randomized study treatment was 88% and the observed blinded major coronary event rate was 2.7% per year. In November 2015, the DMC considered the guidelines for premature stopping (see Supplementary Material) and recommended that the study continue as planned. At a median follow-up of 4 years, REVEAL is anticipated to have 98% power at 2-tailed *P* < .01 to detect a 15% relative risk reduction in the primary outcome.

## Discussion

The REVEAL trial seeks to assess the efficacy and safety of adding anacetrapib 100 mg daily to effective LDL-lowering treatment among 30,449 patients at high risk for cardiovascular events. The prerandomization screening and run-in phase successfully ensured that all participants were receiving appropriate doses of atorvastatin and the resulting mean baseline LDL cholesterol concentration (61 mg/dL) is in line with that anticipated in the study protocol (67 mg/dL).

Extended follow-up of a subset of DEFINE participants showed that anacetrapib has a long terminal half-life such that low levels of anacetrapib (about 8% of apparent steady-state on-treatment trough exposures and 2% of apparent steady-state peak concentrations) were detected in blood 2.5 to 4 years after cessation of therapy.^25^ Preliminary data demonstrate that anacetrapib accumulates in adipose tissue and appears to have a prolonged elimination phase from fat.^26^ Although drug concentrations appear to reach a plateau in plasma by 4 weeks of dosing, adipose tissue concentration of anacetrapib continues to increase for at least 16 weeks of dosing. Up to 1 year after cessation of therapy, only minimal declines in adipose levels of anacetrapib are seen despite a substantial decline in plasma concentration. Preclinical toxicology and exploratory studies performed to date have not indicated any deleterious effects of anacetrapib on adipose tissue structure or function. To explore this issue further, serial adipose tissue biopsies and plasma samples are being taken from several hundred REVEAL participants after at least 18 months of anacetrapib dosing. The planned extended follow-up of all surviving randomized participants (off treatment) will also provide valuable information on the longer-term effects of the study treatment (as has been done for a number of previous lipid treatment trials).27., 28., 29.

By contrast with the ACCELERATE trial of evacetrapib,18., 20. the REVEAL trial of anacetrapib (which has similar effects on lipids) has recruited more participants (30,449 vs 12,092), included a lower proportion with recent acute coronary syndrome (15% vs 31%), has longer duration of follow-up (about 4 years vs around 2.5 years), and involves more primary cardiovascular events (anticipated over 3,000 vs about 1,500). Thus, the REVEAL trial will provide a robust assessment of the efficacy and safety of anacetrapib among individuals at high risk for cardiovascular events who are receiving effective LDL-lowering treatment. The main study results are anticipated later in 2017.

## The REVEAL Collaborative Group

**Steering Committee:** M.J. Landray, L. Bowman (Principal Investigators); R. Collins (Chair); E. Braunwald (Deputy Chair); J.C. Hopewell (Trial Statistician); L. Jiang, C.P. Cannon, S. Wiviott, J. Armitage, R. Haynes, A.P. Maggioni, G. Ertl, C.E. Angermann, T. Pedersen, S. Goto, T. Teramoto (Regional Representatives); A. Gray, B. Mihaylova, C. Baigent, P. Barter, Y. Chen, Z. Chen, J. Tobert, P. Sleight; R. Blaustein^+^, P. DeLucca^+^, Y. Mitchel^+^, G. van Leijenhorst^+^ (^+^ nonvoting Merck representatives)
**Data Monitoring Committee:** P. Sandercock (Chair), D. DeMets, J. Kjekshus, J. Neuberger, A. Tonkin; J. Emberson^+^ (^+^ nonvoting DMC statistician)
**Lipid Monitoring Committee:** C. Granger (Chair), H. Colhoun; K. Wallendszus^+^ (^+^ nonvoting statistical programmer)

## Disclosures

The Clinical Trial Service Unit at the University of Oxford (the regulatory sponsor of REVEAL) has a staff policy of not accepting honoraria or consultancy fees (see https://www.ctsu.ox.ac.uk/about/ctsu_honoraria_25june14-1.pdf).

## Figures and Tables

**Table:** Baseline characteristics of randomized participants.

	Europe	North America	China	Total
Number randomized	15,738	6082	8629	30,449
Age (y)	68 ± 8	67 ± 8	64 ± 8	67 ± 8
Male	13,652 (87%)	5058 (83%)	6824 (79%)	25,534 (84%)
Atorvastatin dose⁎				
Low	7969 (51%)	2621 (43%)	4519 (52%)	15,109 (50%)
High	7769 (49%)	3461 (57%)	4110 (48%)	15,340 (50%)
Prior disease†				
Coronary heart disease	13,306 (85%)	5697 (94%)	7676 (89%)	26,679 (88%)
Cerebrovascular disease	3348 (21%)	1131 (19%)	2302 (27%)	6781 (22%)
Peripheral arterial disease	1645 (10%)	714 (12%)	76 (1%)	2435 (8%)
Diabetes	4678 (30%)	2682 (44%)	3960 (46%)	11,320 (37%)
Heart failure	785 (5%)	343 (6%)	643 (7%)	1771 (6%)
<12 mo since qualifying event	2420 (15%)	1060 (17%)	1933 (22%)	5413 (18%)
Physical measurements				
Systolic blood pressure (mm Hg)	131 ± 18	129 ± 18	133 ± 20	131 ± 19
Diastolic blood pressure (mm Hg)	78 ± 11	76 ± 11	79 ± 12	78 ± 11
Body mass index (kg/m^2^)	29 ± 5	31 ± 6	26 ± 3	29 ± 5
Waist-hip ratio‡	0.97 ± 0.08	0.98 ± 0.08	0.92 ± 0.05	0.96 ± 0.07
Medication				
ACE inhibitor or ARB	11,879 (75%)	4400 (72%)	4050 (47%)	20,329 (67%)
Antiplatelet therapy	14,261 (91%)	5654 (93%)	7992 (93%)	27,907 (92%)
Diuretic	3965 (25%)	1831 (30%)	728 (8%)	6524 (21%)
Calcium-channel blocker	4045 (26%)	1461 (24%)	2679 (31%)	8185 (27%)
β-Blocker	10,897 (69%)	4665 (77%)	5112 (59%)	20,674 (68%)
Biochemistry				
Total cholesterol (mg/dL)	136 ± 21	126 ± 21	129 ± 22	132 ± 21
LDL cholesterol (mg/dL)	63 ± 15	58 ± 14	59 ± 15	61 ± 15
HDL cholesterol (mg/dL)	43 ± 10	37 ± 9	37 ± 8	40 ± 10
Non–HDL cholesterol (mg/dL)	93 ± 19	89 ± 19	91 ± 20	92 ± 19
Triglycerides (mg/dL)	119 (87-167)	122 (87-172)	135 (97-192)	124 (89-174)
Apolipoprotein A1 (mg/dL)	124 ± 19	115 ± 18	116 ± 15	120 ± 18
Apolipoprotein B (mg/dL)	67 ± 13	64 ± 12	61 ± 12	65 ± 13
Lipoprotein(a) (nmol/L)	22 (8-104)	26 (9-111)	22 (10-61)	23 (9-88)
Glomerular filtration rate (ml/min per 1.73 m^2^)‡	81 ± 17	80 ± 18	90 ± 15	83 ± 17
Urinary albumin/creatinine ratio (mg/g)	8 (4-21)	8 (4-29)	8 (4-23)	8 (4-23)

Results are count (%), mean ± SD, or median (interquartile range).

Abbreviations: *ACE*, Angiotensin-converting enzyme; *ARB*, angiotensin receptor blocker.

⁎ Low-dose atorvastatin = 10 mg in China and 20 mg elsewhere; high-dose atorvastatin = 20 mg in China and 80 mg elsewhere.

† Participants may have a history of more than 1 of these conditions.

‡ Estimated using the CKD EPI formula.^30^

## Appendix Supplementary data

Supplementary Methods, Tables and Figures, and Details of the REVEAL Collaborative Group. Image 1

REVEAL Data Monitoring Committee Charter. Image 2

REVEAL Study Protocol. Image 3

REVEAL Data Analysis Plan. Image 4

## References

[1] Cholesterol Treatment Trialists' (CTT) Collaboration (2005). Efficacy and safety of cholesterol-lowering treatment: prospective meta-analysis of data from 90 056 participants in 14 randomised trials of statins. Lancet.

[2] Cannon C.P., Blazing M.A., Giugliano R.P. (2015). Ezetimibe added to statin therapy after acute coronary syndromes. N Engl J Med.

[3] Cholesterol Treatment Trialists' (CTT) Collaboration (2010). Efficacy and safety of more intensive lowering of LDL cholesterol: a meta-analysis of data from 170 000 participants in 26 randomised trials. Lancet.

[4] Prospective Studies Collaboration (2007). Blood cholesterol and vascular mortality by age, sex, and blood pressure: a meta-analysis of individual data from 61 prospective studies with 55,000 vascular deaths. Lancet.

[5] Di Angelantonio E., Sarwar N., Perry P. (2009). Major lipids, apolipoproteins, and risk of vascular disease. JAMA.

[6] Jun M., Foote C., Lv J. (2010). Effects of fibrates on cardiovascular outcomes: a systematic review and meta-analysis. Lancet.

[7] Boden W.E., Probstfield J.L., Anderson T. (2011). Niacin in patients with low HDL cholesterol levels receiving intensive statin therapy. N Engl J Med.

[8] HPS2-THRIVE Collaborative Group (2014). Effects of extended-release niacin with laropiprant in high-risk patients. N Engl J Med.

[9] Thompson A., Di Angelantonio E., Sarwar N. (2008). Association of cholesteryl ester transfer protein genotypes with CETP mass and activity, lipid levels, and coronary risk. JAMA.

[10] Ridker P.M., Pare G., Parker A.N. (2009). Polymorphism in the CETP gene region, HDL cholesterol, and risk of future myocardial infarction: genomewide analysis among 18 245 initially healthy women from the Women's Genome Health Study. Circ Cardiovasc Genet.

[11] Voight B.F., Peloso G.M., Orho-Melander M. (2012). Plasma HDL cholesterol and risk of myocardial infarction: a mendelian randomisation study. Lancet.

[12] Barter P.J., Brewer H.B., Chapman M.J. (2003). Cholesteryl ester transfer protein: a novel target for raising HDL and inhibiting atherosclerosis. Arterioscler Thromb Vasc Biol.

[13] Barter P.J., Caulfield M., Eriksson M. (2007). Effects of torcetrapib in patients at high risk for coronary events. N Engl J Med.

[14] Barter P. (2009). Lessons learned from the Investigation of Lipid Level Management to Understand its Impact in Atherosclerotic Events (ILLUMINATE) trial. Am J Cardiol.

[15] Forrest M.J., Bloomfield D., Briscoe R.J. (2008). Torcetrapib-induced blood pressure elevation is independent of CETP inhibition and is accompanied by increased circulating levels of aldosterone. Br J Pharmacol.

[16] Schwartz G.G., Olsson A.G., Abt M. (2012). Effects of dalcetrapib in patients with a recent acute coronary syndrome. N Engl J Med.

[17] Nicholls S.J., Brewer H.B., Kastelein J.J. (2011). Effects of the CETP inhibitor evacetrapib administered as monotherapy or in combination with statins on HDL and LDL cholesterol: a randomized controlled trial. JAMA.

[18] Nicholls S.J., Lincoff A.M., Barter P.J. (2015). Assessment of the clinical effects of cholesteryl ester transfer protein inhibition with evacetrapib in patients at high-risk for vascular outcomes: rationale and design of the ACCELERATE trial. Am Heart J.

[19] ACCELERATE trial press release. Lilly to Discontinue Development of Evacetrapib for High-Risk Atherosclerotic Cardiovascular Disease. https://investor.lilly.com/releasedetail.cfm?ReleaseID=936130.

[20] Nicholls S.J. (2016). The ACCELERATE trial. Impact of the cholesteryl ester transfer protein inhibitor evacetrapib on cardiovascular outcome. http://www.clinicaltrialresults.org/Slides/ACC%202016/Nicholls_ACCELERATE.pdf.

[21] Cannon C.P., Shah S., Dansky H.M. (2010). Safety of anacetrapib in patients with or at high risk for coronary heart disease. N Engl J Med.

[22] Davidson M., Liu S.X., Barter P. (2013). Measurement of LDL-C after treatment with the CETP inhibitor anacetrapib. J Lipid Res.

[23] Bloomfield D., Carlson G.L., Sapre A. (2009). Efficacy and safety of the cholesteryl ester transfer protein inhibitor anacetrapib as monotherapy and coadministered with atorvastatin in dyslipidemic patients. Am Heart J.

[24] Pocock S.J., Simon R. (1975). Sequential treatment assignment with balancing for prognostic factors in the controlled clinical trial. Biometrics.

[25] Gotto A.M., Cannon C.P., Li X.S. (2014). Evaluation of lipids, drug concentration, and safety parameters following cessation of treatment with the cholesteryl ester transfer protein inhibitor anacetrapib in patients with or at high risk for coronary heart disease. Am J Cardiol.

[26] Gutstein D., Krishna R., Johns D. (2015). Observed long plasma terminal half life of anacetrapib (ANA) is associated with adipose deposition: plasma and adipose pharmacokinetics (PK) in mice and humans. Clin Pharmacol Ther.

[27] Bulbulia R., Bowman L., Wallendszus K. (2011). Effects on 11-year mortality and morbidity of lowering LDL cholesterol with simvastatin for about 5 years in 20,536 high-risk individuals: a randomised controlled trial. Lancet.

[28] Sever P.S., Chang C.L., Gupta A.K. (2011). The Anglo-Scandinavian Cardiac Outcomes Trial: 11-year mortality follow-up of the lipid-lowering arm in the U.K. Eur Heart J.

[29] Ford I., Murray H., McCowan C. (2016). Long-term safety and efficacy of lowering low-density lipoprotein cholesterol with statin therapy: 20-year follow-up of West of Scotland Coronary Prevention Study. Circulation.

[30] Levey A.S., Stevens L.A., Schmid C.H. (2009). A new equation to estimate glomerular filtration rate. Ann Intern Med.

