# Supplementary material for: Randomized Evaluation of the Effects of Anacetrapib through Lipid-modification (REVEAL)—A large-scale, randomized, placebo-controlled trial of the clinical effects of anacetrapib among people with established vascular disease: Trial design, recruitment, and baseline characteristics
Source: Am Heart J. 2017 May;187:182–90. doi: 10.1016/j.ahj.2017.02.021 (PMC5419667; doi:10.1016/j.ahj.2017.02.021)
Supplement: Supplementary file 1 — Supplementary Methods, Tables and Figures, and Details of the REVEAL Collaborative Group. [file mmc1.docx]

**Randomized Evaluation of the Effects of Anacetrapib through Lipid-modification (REVEAL) – A large-scale, randomized, placebo-controlled trial of the clinical effects of anacetrapib among people with established vascular disease: trial design, recruitment and baseline characteristics**

**Supplementary Material**

Table of Contents

[Supplementary Methods 3](#_Toc472857412)

[Study organization and funding 3](#_Toc472857413)

[Management of LDL cholesterol and determination of atorvastatin dose 3](#_Toc472857414)

[Post-randomization follow-up and safety monitoring 4](#_Toc472857415)

[Central laboratory analyses and storage 5](#_Toc472857416)

[Adverse event reporting and outcome adjudication 6](#_Toc472857417)

[Myocardial infarction 6](#_Toc472857418)

[Coronary revascularization procedures 6](#_Toc472857419)

[Stroke 6](#_Toc472857420)

[Non-coronary revascularization procedures 7](#_Toc472857421)

[Myopathy and rhabdomyolysis 7](#_Toc472857422)

[Liver-related outcomes 8](#_Toc472857423)

[Death 8](#_Toc472857424)

[Sample size and anticipated number of events 8](#_Toc472857425)

[Randomization 9](#_Toc472857426)

[Data analysis plan 9](#_Toc472857427)

[Exclusion of data from one site due to significant protocol breaches 10](#_Toc472857428)

[Interim analyses: role of the Data Monitoring Committee 10](#_Toc472857429)

[Details of the REVEAL Collaborative Group 11](#_Toc472857430)

[Writing Committee 11](#_Toc472857431)

[Steering Committee 11](#_Toc472857432)

[Data Monitoring Committee 11](#_Toc472857433)

[Lipid Monitoring Committee 11](#_Toc472857434)

[Coordinating Centres 11](#_Toc472857435)

[Local Clinical Centres 12](#_Toc472857436)

[Supplementary References 24](#_Toc472857437)

[Supplementary Tables and Figures 25](#_Toc472857438)

[Supplementary Table I: Recruitment by country and region 26](#_Toc472857439)

[Supplementary Table II: Reasons screened patients did not enter run-in 27](#_Toc472857440)

[Supplementary Table III: Reasons for withdrawing from the run-in period 28](#_Toc472857441)

[Supplementary Table IV: Reasons for exclusion at randomization visit 29](#_Toc472857442)

[Supplementary Table V: Baseline characteristics of randomized participants 30](#_Toc472857443)

[Supplementary Table VI: Baseline LDL cholesterol among randomized participants 33](#_Toc472857444)

[Supplementary Table VII: Schedule of central laboratory procedures 34](#_Toc472857445)

[Supplementary Figure 1: REVEAL trial design 35](#_Toc472857446)

[Appendix A: Determination of atorvastatin dose at screening visit 36](#_Toc472857447)

[Table A: Anticipated effect of pre-study lipid treatment on total cholesterol 37](#_Toc472857448)

# Supplementary Methods

Further details about the study design and procedures are described in the Protocol, the Data Monitoring Committee (DMC) Charter, and the Data Analysis Plan, all of which are published with this paper.

## Study organization and funding

The study was designed by the independent investigators at the Clinical Trial Service Unit (CTSU) at the University of Oxford in collaboration with the Thrombolysis in Myocardial Infarction (TIMI) Study Group based at Brigham and Women’s Hospital, Boston, and Merck & Co., Inc. (Merck), which manufactures anacetrapib. The University of Oxford acts as the regulatory sponsor and retains the study database for all published analyses (including regulatory submissions).

The study is funded by a grant to the University of Oxford from Merck to cover the costs of central coordination and all costs in the UK, Germany, Italy and China; a grant from Merck to the TIMI Study Group to cover the costs in the USA and Canada; and with direct funding provided by Merck to its subsidiaries in the Scandinavian countries to cover the costs within those countries. Packaged anacetrapib and matching placebo tablets, and atorvastatin tablets, are provided by Merck direct to the local sites.

The study is conducted by an international collaboration between the CTSU at the University of Oxford; the TIMI Study Group at Brigham and Women's Hospital in Boston, MA; the University Hospital and University of Würzburg in Würzburg, Germany; the Associazione Nazionale dei Medici Cardiologi Ospedalieri (ANMCO) in Florence, Italy; the China-Oxford Centre for International Health Research in Beijing, China; and investigators in Denmark, Finland, Norway and Sweden.

The independent Steering Committee is responsible for drafting the main reports from the study and for review of any other reports. The study Steering Committee includes representatives from the central and regional coordinating centres, cardiologists, clinical trialists, statisticians and non-voting observers from the funder. Merck has non-voting membership on the Steering Committee, and provides trial coordination within Scandinavia through its subsidiaries (under the direction of and monitored by the University of Oxford). Although Merck has the opportunity to comment on drafts of manuscripts, it otherwise has no role in the collection and analysis of the data presented, the preparation and approval of this manuscript, or the decision to submit it for publication.

## Management of LDL cholesterol and determination of atorvastatin dose

All participants who entered the run-in phase were issued with an atorvastatin regimen that was intended to reduce their LDL cholesterol to below 77 mg/dL (2 mmol/L) and which was at least as intensive as their current LDL-lowering treatment.

The Reflotron Plus (Roche) dry chemistry analyser provides an immediate measurement of total cholesterol (not LDL cholesterol) in mmol/L. At the Screening Visit, individuals were excluded if they were already receiving LDL-lowering treatment that was more intensive than the maximum atorvastatin dose available in the trial. A computer algorithm (see [Appendix A](#_Appendix_A:_Determination)) used the dry chemistry total cholesterol measurement and the type and dose of their current LDL-lowering therapy (statin and/or ezetimibe) to determine eligibility and the minimum dose of study atorvastatin (either 10 or 20 mg in China, 20 or 80 mg in other countries) likely to be required to lower total cholesterol to less than 135 mg/dL (3.5 mmol/L). Individuals were excluded if they were already receiving LDL-lowering treatment that was more intensive than the maximum atorvastatin dose available in the trial or if it was unlikely that they would achieve the target total cholesterol on that dose of atorvastatin. In addition, at the end of the run-in phase on study atorvastatin, individuals were excluded at the Randomization Visit if their total cholesterol (measured using the Reflotron Plus) was greater than 4 mmol/L (155 mg/dL),

Estimates based on the lipid profiles of similar patient populations in the HPS2-THRIVE study^1^ anticipated that this approach would result in a mean baseline LDL cholesterol of about 67 mg/dL (1.7 mmol/L) among randomized participants and that around 15% of the randomized participants would have LDL cholesterol ≥77 mg/dL (2.0 mmol/L).

As a result of using this algorithm, the mean LDL cholesterol at the Randomization visit for those participants who were randomized into the study was 61 mg/dL (SD 15 mg/dL; Table I), including 3874 (13%) randomized participants with LDL cholesterol ≥77 and <97 mg/dL (≥2.0 and <2.5 mmol/L) and 423 (1%) participants with LDL cholesterol ≥97 mg/dL (≥2.5 mmol/L; see [Supplementary Table VI](#_Supplementary_Table_VI:)).

## Post-randomization follow-up and safety monitoring

Following randomization, it is intended that follow-up information is collected from all participants for the full duration of the study, irrespective of whether they continue to take study treatment or suffer a study outcome. Clinic Follow-up Visits are scheduled at 2 and 6 months initially (with an additional visit at 4 months in China, at the request of the Chinese Food and Drug Administration), and then 6-monthly until the end of the study. Additional “Early Recall” visits are arranged for any participant who requires review outside their planned visit schedule (e.g. for further evaluation of abnormal safety blood results or review of symptoms believed to be related to study treatment).

At each Follow-up or Early Recall Visit, study staff seek details of all SAEs (including study outcomes and reasons for hospitalization), any non-serious adverse events attributed to or resulting in discontinuation of study treatment, and any symptoms of muscle pain or weakness or suggestive of hepatitis (loss of appetite, nausea, jaundice, lethargy or malaise). At the request of the US Food and Drug Administration, information on all other non-serious adverse events is sought for participants in North America. Changes to non-study treatment, compliance with and reasons for discontinuation of study medication, and blood pressure are recorded at each visit. At the final visit, weight and hip, waist and neck circumference are to be measured, and quality of life and cognitive function are to be assessed. A non-fasting blood sample is taken for immediate dry chemistry analysis of ALT. Depending on the extent of any elevation in ALT and the presence of symptoms suggestive of hepatitis or myopathy, additional measurements of alkaline phosphatase, bilirubin, CK and creatinine may be required as described in the protocol. A further 7-month supply of study treatment is issued to those participants who are able to continue on study treatment. If a participant becomes unwilling or unable to attend study visits in person, then follow-up information can be collected by telephone, direct correspondence with participant’s own doctors, or review of routine healthcare systems and registries.

All reported adverse events, reasons for stopping randomized treatment and locally measured blood results are reviewed daily by clinicians at the Central Coordinating Office. Modifications to study treatment and arrangements for additional clinic visits and investigations are made according to pre-defined study procedures (see Protocol). If safety monitoring of blood is no longer possible (e.g. because the participant no longer attends clinic visits), then the participant is asked to discontinue all study treatment and advised to see their own doctor so that alternative LDL-lowering therapy can be considered. Study treatment can also be modified in the event of an SAE likely to be due to one or more of the study treatments, current use of a contraindicated medication (as described in the exclusion criteria; see Figure 2), at the request of the participant or their doctors (for any reason), or in any other situation where continuing study treatment is not considered to be in the participant’s best interests. The dose of study atorvastatin can be modified or stopped (and an alternative LDL-lowering regimen prescribed if considered appropriate) at the discretion of a participant’s managing doctor.

Wherever possible, extended follow-up (off study treatment) of all surviving randomized participants will continue for at least 2 years beyond the final study visit in order to provide information on the longer-term safety and efficacy of anacetrapib. Information on serious adverse events (including cause-specific mortality, cancers, and cardiovascular events) will be sought for all surviving and consenting REVEAL participants. Follow-up will be by 6-monthly telephone calls with additional information collected by other means (e.g. linkage to routine data sources) depending on local circumstances (e.g. availability of national health registry data).

## Central laboratory analyses and storage

Non-fasting blood samples are scheduled to be taken for central laboratory assays and storage from all participants at the randomization and 2 month visits, at a Follow-up Visit about 2 years after the median participant was randomized, and at the final visit, and from around 5% of participants annually. A urine sample is taken for central analysis and storage at the Randomization and final Follow-up Visits. Lipid and lipoprotein analyses are performed on all of these samples while glycosylated haemoglobin, plasma creatinine, and urinary albumin:creatinine ratio are measured on the randomization and final follow-up samples only (see [Supplementary Table VII](#_Supplementary_Table_VII:_1)). Samples of genetic material, plasma, serum and urine are being stored long-term for future analysis, subject to appropriate consents.

Samples are processed at the local sites and stored at -20°C or below before frozen transfer to the central laboratory at the University of Oxford. Creatinine and lipids (total cholesterol, LDL cholesterol, HDL cholesterol, triglycerides, apolipoproteins B and A1, and lipoprotein(a)) in EDTA plasma are assayed using Beckman-Coulter AU680 clinical chemistry analysers (with LDL cholesterol also measured by beta quantification in a sub-study of around 2000 participants). Creatinine and albumin in urine are assayed using Beckman-Coulter DxC800 clinical chemistry analysers. Plasma and urine assays use manufacturers’ reagents, calibrators and settings (Beckman-Coulter, UK), except lipoprotein(a) which is performed using the Denka Seiken method with reagents and calibrators supplied by Randox Laboratories Ltd, UK. Lipoprotein(a) measures are certified for accuracy by the Northwest Lipid Metabolism And Diabetes Research Laboratories, Seattle, USA.^2^ Glomerular filtration rate is estimated using the CKD-EPI formula.^3^ HbA1c analysis is performed by HPLC using EDTA whole blood on an Arkray HA8160 analyser and reagents with a calibrator supplied by Menarini Diagnostics UK traceable to International Federation of Clinical Chemistry (IFCC) reference standards. The central laboratory is a UKAS accredited testing laboratory, No. 2799. All assays use methods accredited by the UK Accreditation Service except Lipoprotein(a) and HbA1c analysis for Chinese samples, which are to be done by a commercial laboratory in Beijing with quality oversight by the central laboratory.

## Adverse event reporting and outcome adjudication

Adverse events are recorded directly into the study IT system by study staff at local clinical sites or trained clinical staff at the Central Coordinating Office by selecting from around 2000 MedDRA Preferred Terms (version 14.0). For about 9% of adverse events, the initial report is made as free-text. In such cases, a suitable code is assigned, blind to study treatment allocation, by study clinicians at the Central Coordinating Office. For the purposes of analysis, Preferred Terms are grouped by primary MedDRA System Organ Class, except that priority is given to “Neoplasms benign malignant and unspecified (inc cysts and polyps)” and “Infections and infestations” in that order.

Additional supporting clinical information is sought for any reports of SAEs that might be importantly relevant to the assessment of the efficacy or safety of the study treatment (e.g. events reported as MI, angina, stroke, revascularization procedure, cancer, myopathy, rhabdomyolysis, non-viral hepatitis, or death). Potential study outcomes are adjudicated by clinicians based at or overseen by the Central Coordinating Office, blind to study treatment allocation or blood lipid values and using standard definitions outlined below. For events with supporting documentation that is not in the native language of the adjudicator (typically any language other than English or Chinese), a clinically qualified adjudication assistant provides simultaneous translation and interpretation of the relevant sections of supporting documents for the adjudicator (who remains responsible for all adjudication decisions).

### Myocardial infarction

The term myocardial infarction (MI) is to be used when there was evidence of myocardial necrosis consistent with myocardial ischaemia in an appropriate clinical context.^4^ This requires evidence of cardiac necrosis provided by either cardiac biomarker results or autopsy with supporting evidence of an appropriate clinical context (e.g. ischaemic symptoms, electrocardiographic changes of new ischaemia, or imaging showing a new myocardial defect or evidence of acute coronary occlusions); and no other likely diagnosis. Silent MI is not to be included in the analysis.

### Coronary revascularization procedures

Coronary revascularization includes coronary artery grafting or angioplasty (with or without endovascular stenting) as well as other percutaneous coronary interventions designed to treat coronary artery lesions (e.g. atherectomy, embolectomy). Failed procedures (e.g. placement of a coronary guide wire for angioplasty but no balloon inflated) are to be coded as a coronary revascularization procedure. Angiographic studies without definite evidence of intervention are not to be included. Wherever possible, coronary revascularization procedures are to be adjudicated as urgent (primary), urgent (other) or non-urgent. The analysis of urgent and non-urgent coronary revascularization is a tertiary assessment – all coronary revascularization procedures are to be included in the primary and secondary outcomes.

### Stroke

Stroke is defined as an acute symptomatic episode of focal or global neurological dysfunction caused by brain, spinal, or retinal vascular injury as a result of haemorrhage or infarction. Stroke subtype is assessed based on the available neuro-imaging or autopsy results as follows:

- Haemorrhagic stroke: Stroke caused by a non-traumatic intraparenchymal, intraventricular or subarachnoid haemorrhage
- Ischaemic stroke: Stroke caused by infarction of central nervous system tissue. This includes cases in which imaging and/or pathological examination identifies a definite infarct, identifies an infarct with haemorrhagic transformation, or shows no lesion.
- Underdetermined stroke: Stroke of unknown/unconfirmed pathological type, i.e. a stroke for which it is unclear whether there is an ischaemic or haemorrhagic cause because imaging was not performed or the result is not available.

Transient ischaemic attack (TIA) is defined as a transient episode (i.e. full recovery within 24 hours) of neurological dysfunction caused by brain, spinal cord, or retinal ischaemia, without clear evidence of acute infarction, haemorrhage or trauma. For the purposes of analysis (including secondary outcomes), presumed ischaemic stroke includes both ischaemic stroke and undetermined stroke. TIA is not a pre-defined endpoint and is not to be included in analyses of stroke outcomes.

### Non-coronary revascularization procedures

Non-coronary revascularization includes percutaneous/endovascular interventions (e.g. angioplasty, atherectomy, thrombectomy, embolectomy, catheter directed thrombolysis, or endovascular aneurysm repair), surgical procedures (e.g. arterial bypass surgery, open angioplasty, endarterectomy, thrombectomy, embolectomy, or aneurysm surgery), or major amputation (i.e. proximal to the ankle or wrist) for vascular disease. Angiographic studies without definite evidence of intervention are not to be included.

*Diabetes mellitus*

Diabetes mellitus does not require formal adjudication at either baseline or during follow-up.

- Diabetes mellitus at baseline is defined as self-reported diabetes on the Screening or Randomization Visit form; a diabetes-related adverse event recorded on or before the date of randomization; or the use of hypoglycaemic medication (insulin or oral treatment) on the Randomization Visit form.
- New onset diabetes is defined as a post-randomization diabetes-related adverse event (that is a MedDRA Preferred Term that maps to either the High Level Term “Diabetes mellitus (including subtypes)” or the High Level Group Term “Diabetic complications”, excluding “Microalbuminuria”) or the use of hypoglycaemic medication (insulin or oral treatment) recorded on at least one Follow-up Visit form for a person without evidence of diabetes mellitus at baseline.

Although glycosylated haemoglobin A1c (HbA1c) is to be measured at baseline and at final follow-up visit for all participants, it does not form part of the pre-specified definition of diabetes mellitus.

### Myopathy and rhabdomyolysis

Muscle-related outcomes are chiefly classified on the basis of elevations in creatine kinase (CK) and the presence/absence of muscle symptoms. Biochemical results and muscle symptoms do not require adjudication unless CK>10x upper limit of normal (ULN). Rhabdomyolysis is defined as CK>40x ULN with muscle symptoms or CK>10x ULN with evidence of end-organ damage such as doubling of serum creatinine. For all cases of rhabdomyolysis or myopathy, the aetiology is to be assessed, in particular to discriminate cases that may be related to study atorvastatin and/or anacetrapib from other causes (e.g. fall/trauma, hypothyroidism, or a non-study medication).

### Liver-related outcomes

Liver injury is defined based on biochemical results or adverse events (ALT >3x ULN plus bilirubin >2x ULN with CK ≤5x ULN; or ALT >10x ULN; liver transplantation; or death due to liver disease). For all cases of liver injury, the aetiology is to be assessed, in particular to discriminate cases that may be related to randomized treatment from other causes such as infections (e.g. hepatitis viruses), non-infectious processes (e.g. non-alcoholic fatty liver disease, autoimmune hepatitis), secondary processes (e.g. heart failure), toxicity (e.g. alcohol), and pharmacological causes due to study atorvastatin or non-study medication.

Cancer

Confirmation of cancer site and date of occurrence (in particular, pre-/post-randomization) is to be sought for a participant’s first post-randomization report of cancer of a particular site and for all fatal cancer events. Non-melanoma skin cancer is not adjudicated.

### Death

The fact, date and cause of death are confirmed using the available information. If the death is due to an event in one of the above categories, the relevant criteria are to be applied. Coronary death includes deaths due to MI (see definition above), other consequences of coronary atherosclerosis (e.g. ischaemic heart failure) and sudden cardiac death. Other cardiac death includes deaths thought to be due to other cardiac disease (e.g. valvular heart disease). Deaths are only classified as being of unknown cause if a cause cannot be established (e.g. a death for which there is no information beyond “patient died”, despite best efforts to obtain further details).

## Sample size and anticipated number of events

Using blinded data from the then on-going HPS2-THRIVE trial, the rate of non-fatal MI or coronary death was anticipated to be about 0.9% per annum (pa) in both China and in Europe.^1^ When coronary revascularization procedures were added, event rates were estimated to be approximately twice as large: 1.8% pa in China and 1.7% pa in Western Europe.

Data from the HPS2-THRIVE trial were used to model the effects on lipids of adding anacetrapib 100 mg daily to the background atorvastatin therapy. It was estimated that full compliance with anacetrapib 100 mg daily would increase HDL cholesterol by 50 mg/dL (1.3 mmol/L) from 39 mg/dL to 89 mg/dL (1.0 mmol/L to 2.3 mmol/L) and decrease LDL cholesterol by 27 mg/dL (0.7 mmol/L) from 67 mg/dL to 40 mg/dL (1.7 mmol/L to 1.0 mmol/L). In estimating the likely effects on clinical outcomes, it was assumed that only about three-quarters of the participants would be taking their allocated study treatment at the midpoint of study follow-up.

There is good evidence from randomized trials that, for every 39 mg/dL (1 mmol/L) reduction in LDL cholesterol achieved with a statin, the risk of major coronary events is reduced by about one-fifth over a 4-5 year period (with a smaller effect in the first year compared with each subsequent year).^5,6^ Anacetrapib reduces LDL cholesterol by a different mechanism to that of statins. But, if the relationship between LDL cholesterol reduction and risk reduction is similar, the anticipated 19 mg/dL (0.5 mmol/L) reduction in LDL cholesterol (assuming three-quarters compliance at the study midpoint) would be expected to translate into a 10-15% relative reduction in major coronary events. There are insufficient data from randomized trials to make reliable estimates of the effects of increasing HDL cholesterol on the risks of such events. In the Emerging Risk Factor Consortium meta-analysis of prospective observational studies, 30 mg/dL (0.8 mmol/L) higher HDL cholesterol was associated with about two-fifths lower risk of non-fatal MI or coronary death.^7^ If, as with LDL cholesterol, only about half of the effect associated epidemiologically emerges within about 4-5 years of raising HDL cholesterol, then the anticipated 39 mg/dL (1 mmol/L) increase (assuming three-quarters compliance) would be expected to translate into a 20-25% relative reduction in major coronary events.

These assumptions were re-evaluated prior to finalization of version 2.0 of the Protocol. In particular, based on an observed blinded major coronary event rate of 2.7% per annum and median follow-up of 4 years, a trial of 30 449 participants (i.e. the number randomized) would have 98% power at two-tailed P<0.01 to detect a 15% relative risk reduction.

## Randomization

Eligible and consenting individuals were allocated anacetrapib or placebo using a minimized randomization program embedded within the clinic IT system that helps maximize balance between the treatment groups with respect to prognostically important baseline variables (age, gender, history of prior vascular disease, diabetes mellitus, smoking status, dry chemistry total cholesterol, systolic blood pressure, race, years of prior statin use, and study atorvastatin dose).^8^ The algorithm included a stochastic element (treatment was assigned to the arm determined by the minimization algorithm with a probability of 0.9).

## Data analysis plan

The data analysis plan was pre-specified in the original protocol. The primary outcome for the assessment of efficacy is Major Coronary Event, which is defined as coronary death, MI or coronary revascularization. The secondary outcomes were revised by the Steering Committee in November 2015 and the final data analysis plan was published on the study website ([www.revealtrial.org](http://www.revealtrial.org/)) in June 2016 prior to any unblinded analyses being available to any members of the Steering Committee. A copy of that Data Analysis Plan is included with this supplementary material. In addition to the analyses specified there-in, the original study protocol included pre-specified sub-group analyses of efficacy. To this end, REVEAL participants will be divided into 3 risk subgroups using the previously published 9-factor TIMI Risk Score for Secondary Prevention (TRS 2^o^P), as follows: low risk (0-1 risk indicators), intermediate risk (2 risk indicators), and high risk (≥3 risk indicators).^9^ In addition, a new risk score will be derived from the REVEAL baseline clinical data (blinded to treatment allocation) and participants will be divided into low, intermediate and high risk groups based on tertile of risk score. The discriminatory performance of these two scores will be compared. Unless, the *de novo* score materially out-performs the TRS 2^o^P score, the latter will be used for subgroup analyses of the effect of anacetrapib vs placebo on the primary outcome of major coronary event and the secondary outcome of major vascular event. As indicated in the Data Analysis Plan, if there is directional consistency in the effect of the treatment on the primary outcome and on presumed ischaemic stroke, emphasis will be placed on the subgroup analyses for the secondary outcome of major vascular event (which incorporates both outcomes). In due course, additional analyses of efficacy by vascular risk will be conducted using scores that include information provided by subsequent genetic and/or biochemical assays.

The impact of outcome adjudication on study conclusions will be explored in subsidiary analyses. The estimates of treatment effect derived from unadjudicated data on the primary and secondary outcomes, and additional assessments requested by regulatory agencies will be compared with those derived from fully adjudicated data (the main analyses).

### Exclusion of data from one site due to significant protocol breaches

In May 2013, concerns were raised relating to whether the correct procedures had been followed for informed consent, ascertainment and confirmation of study safety and efficacy outcomes, and the validity of locally recorded blood results at a single US study site which had purportedly screened 259 participants and randomized 174 of them. Following detailed investigation, it was felt that additional monitoring or other measures were unlikely to be able to improve site performance. The site was closed in an orderly fashion and, to the extent reasonably possible, all participants were recalled for a final study visit in order to retrieve all study treatment and to provide them with study contact information should they have further concerns. The site was closed in July 2013. Notification of the closure and a detailed report of the investigation and its findings were provided to the Steering Committee, the DMC, the study sponsor (University of Oxford), the funder (Merck), ethics committees responsible for the site and the sponsor, the US Food and Drug Administration and the UK Medicines and Healthcare products Regulatory Agency. In view of this serious breach of Good Clinical Practice, the Steering Committee determined (without knowledge of any unblinded results) at its meeting in February 2014 that all data from this site should be excluded from all analyses. Thus the data analyses (including all tables and figures) in the main paper and this supplementary material do not include data from this site.

## Interim analyses: role of the Data Monitoring Committee

During the study, interim analyses of all serious adverse events and other study outcomes (both overall and in key subgroups, including by region and by baseline lipid levels) were supplied in strict confidence to the independent DMC. The DMC was able to request such analyses at a frequency relevant to the stage of the study (typically at 6-12 monthly intervals, with a Chairman’s review every 3-6 months) or in response to emerging data from other trials. Unless advised by the DMC in response to clear evidence of benefit or hazard, the Steering Committee, collaborators, participants, representatives of Merck, and all study staff (except those who provide the confidential analyses to the DMC) were to remain blind to the interim results on mortality and major morbidity until the end of the study. The responsibilities of the independent DMC, including guidelines for consideration of stopping for benefit, safety or futility are described in the DMC Charter.

# Details of the REVEAL Collaborative Group

## Writing Committee

L Bowman, F Chen, E Sammons, JC Hopewell, K Wallendszus, W Stevens, E Valdes-Marquez, S Wiviott, CP Cannon, E Braunwald, R Collins, MJ Landray

## Steering Committee

MJ Landray, L Bowman (Principal Investigators); R Collins (Chair); E Braunwald (Deputy Chair); JC Hopewell (Trial Statistician); L Jiang, CP Cannon, S Wiviott, J Armitage, R Haynes, AP Maggioni, G Ertl, CE Angermann, T Pedersen, S Goto, T Teramoto (Regional representatives); A Gray, B Mihaylova, C Baigent, P Barter, Y Chen, Z Chen, J Tobert, P Sleight; R Blaustein^+^, P DeLucca^+^, Y Mitchel^+^, G van Leijenhorst^+^ (^+^ non-voting Merck representatives)

## Data Monitoring Committee

P Sandercock (Chair), D DeMets, J Kjekshus, J Neuberger, A Tonkin; J Emberson^+^ (^+^ non-voting DMC statistician)

## Lipid Monitoring Committee

C Granger (Chair), H Colhoun; K Wallendszus^+^ (^+^ non-voting statistical programmer)

## Coordinating Centres

Central Coordinating Office and UK (Clinical Trial Service Unit, University of Oxford):

*Management Committee:* M Landray, L Bowman (principal investigators), J Barton, C Bray, R Dayanandan, C Knott, M Lay, K Murphy, E Wincott; *Administration and support:* J Barton, C Bray, R Dayanandan, K Murphy, E Wincott (coordinators), P Achiri, S Barry, S Bateman, A Brewster, S Briggs, R Brown, A Burke, E Butler, L Cobb, A Collet, J Crowther, L Cureton, S Danesh-Pour, S Fathers, L Fletcher, K Frederick, T Gordon, M Gray, J Heineman, S Howard, D Jackson, N Lam, R Lee, O Machin, Z Madgwick, M Matthewson, J Nolan, M Nunn, A Panicker, L Pank, E Pearson-Burton, S Pickworth, Y Qiao, A Radley, K Roby, J Sayer, S Shah, K Taylor, H Thorne, A Timadjer, K Vandenberg, M Wickman, M Willett, J Woods, H Yu; *Clinical support and adjudication:* J Armitage, T Aung, L Bowman, R Bulbulia, F Chen, R Clarke, R Haynes, W Herrington, P Judge, MJ Landray, D Lewis, R Llewellyn-Bennett, M Mafham, D Preiss, C Reith, E Sammons, B Storey, J Tomson, E Waters; *Computing & validation:* A Baxter, M Lay, R Goodenough (coordinators); R Ait-Sadi, M Arnold, I Barton, C Berry, G Blower, J Booth, E Brown, Y Bu, P Cleverley, G Coates, J Cox, M Craig, G Cui, P Dalton, L Danel, C Daniels, C Dawe, A Field, S Gilbert, P Harding, K Jayne, R Kurien, G Lancaster, A Maskill, A McDougall, Y Mostefai, S Mulay, A Munday, A Murawska, N Prajapati, S Ramesh, R Reid, S Syed, H Todd, A Young, A Young, W Zhu; *Statistical analysis:* JC Hopewell (study statistician), K Wallendszus (statistical programming coordinator), M Arnold, S Parish, W Stevens, E Valdes-Marquez; *NDPH Wolfson Laboratory:* M Hill (laboratory director), S Clark, K Emmens, G McClean, M Radley, J Wintour (laboratory coordinators); M Allworth, A-M Beneat, L Boggs, T Chavagnon, K Chung, R Chung, R Cox, L Finnegan, H French, N Goodwin, A Gordon, J Gordon, C Guest, S Hazim, J Hill, R Hrusecka, M Lacey, N Luker, S Mulligan, N Plunkett, L Sansom, R Shellard, J Taylor, P Taylor, J Tyler, L Weaving, J Wheeler, T Williams, M Yeung; *Monitors and trainers:* C Knott (coordinator); S Beebe, K Bowsher-Brown, J Dabrowski, J Henderson, J James, H Lochhead, V Toghill, L Wright, L Young;

China (China-Oxford Centre for International Health Research, Fuwai Hospital, Beijing):

*Regional coordinator:* L Jiang; *Clinical support and adjudication:* W Hundei, J Liu, J Qu, H Zhang; *Administration and support:* H Dai, F Feng, L Hou, J Li, L Ma, S Niu, R Tang, S Wang, X Wei, M Xie, X Yan, M Yang, Y Zhang, L Zhang, A Zhang, S Zhang, L Zhao, H Zhong; *Monitors and Trainers:* L Chen, Y Gao, L Li, H Yang; *IT Support:* J Zhang;

Germany (Comprehensive Heart Failure Center [CHFC] and Department of Internal Medicine I, University Hospital and University of Würzburg, Würzburg):

*Regional coordinators:* CE Angermann, G Ertl, C Wanner; *Clinical support and adjudication:* S Brenner, M Heldmann, B Kraus, B Meyer; *Administration and support:* M Fajardo-Moser, C Hartner; *Monitors and Trainers:* A Knoppe, D Pop-Marschall, U Saemann;

Italy (Associazione Nazionale dei Medici Cardiologi Ospedalieri [ANMCO], Florence):

*Regional coordinator:* AP Maggioni; *Clinical support and adjudication:* G Fabbri; *Administration and support:* A Lorimer, D Lucci, B Bartolomei Mecatti; *Monitors and Trainers:* M Ceseri, E Baldini, S Benoni, F Bianchini, P Ferruzzi, M Miccoli, S Musio, F Ramani; *IT Support:* M Gorini, G Orsini;

Japan (School of Medicine Multiregional Study Office, Tokai University):

*Regional coordinators:* S Goto, T Teramoto; *Clinical support and adjudication:* E Kato, K Tawara, A Tomita; *Administration and support:* S Kitamura, Y Saitoh, M Shimizu, S Shiozaki, K Soeda, A Tanaka; *Monitors and Trainers:* E Kato; K Tawara; A Tomita;

North America (TIMI Study Group, Boston):

*Regional coordinators:* E Braunwald, C Cannon, S Wiviott; *Clinical support and adjudication:* A Eisen, E Kato, D Steen; *Administration and support:* P Fish (director of operations), S MacDonnell, J Kent, A McCagg (lead coordinators), E Greene, D Klements, K Washington; *Monitoring and training (Covance, Princeton):* M Goeres, J Koen (lead monitors), J Colicchia, A Davis, J Domercant, V Foster, C Fox, C Gennusa, R Hollis, Y Kassa, A Kelley, V Magloire, C Owens, N Yeh;

Scandinavia (MSD, Copenhagen):

*Regional coordinator:* T Pedersen (*Ullevål Universitetssykehus HF, Oslo*); *Coordinators:* K Arnesson, S Mosegaard; *Clinical support and adjudication:* K Andersen, S Haywood, A Osmanagic, C Pilgaard Madsen, E Rebnord, K Serup-Hansen, M Tarras Wahlberg; *Administration and support:* Denmark: K Hannibal, T Johansen, L Rasmussen, A Sloth; Finland: P Kiuru, M Lauronen, A-S Leinonen, T Mononen, M Vuola, S Wiik; Norway: H Hovdal, C Lien, S Svingen, P Singh, I Thorsby, E Westerheim; Sweden: P Bergsten, L Bergvall, H Castedal, A-C Cederholm, L Froberg, A Johansson, L Jonsson, P Martin, T Rasmusson, S Wiik-Karu; *Monitoring and training:* Denmark: H Diget, O Moll, S Snejbjerg, G Sørensen; Finland: S Eronen, S Roine, T Vaine; Norway: V Bjørhovde, L Edvardsen, S Saether; Sweden: Å Blechert, I Ek, L Hedlöf, J Levin, D Vlaheli;

## Local Clinical Centres

Canada

*National coordinator:* J Genest; *Collaborators: Brampton Research Associates, Brampton, ON:* M Gupta, A Burgess, C Dela Cruz, S Harnden, S Hirjikaka, E Mallari, Y Thevakumaran; *Cambridge Cardiac Care Inc., Cambridge, ON:* A Pandey, J Lake, M Pandey, C Wang; *Centre de Dépistage et Recherche Cardiovasculaire Rive-Sud, Longueuil, QC:* E Sabbah, I Chausse, F Deslongchamp, J Lavoie; *Centre de Recherche Medialpha, St. Lachine, QC:* G Sabe-Affaki, S Fontaine; *Centre intégré de santé et de services sociaux de Lanaudière - Hôpital Pierre-Le Gardeur, Terrebonne, QC:* G Gosselin, M David, K Drouin, N Lachance, C Masson, M Pashko, C Tremblay; *Clinique Sante Cardio MC, Montreal, QC:* C Constance, M Gauthier; *CSSSNL/CHRDL, Saint-Charles-Borromee, QC:* S Kouz, C Fleury, V Lemay-Chretien, N Roberge, M Roy; *Discovery Clinical Services LTD, Victoria, BC:* G Hoag, R Standring, L Warke; *Dr. Stephen Pearce, Inc, Surrey, BC:* S Pearce, L Breakwell, T Cleveland, D Kastanis; *Heart Care Research, Oshawa, ON:* R Bhargava, C Stafford, C Stata; *Heritage Medical Research Clinic, Calgary, AB:* T Anderson, D Brown, B Madden, M Pajevic, D Ramadan, B Smith; *James Cha MD, Oshawa, ON:* J Cha, J Otis; *Newmarket Cardiology Research Group, Newmarket, ON:* R Zadra, A Harwood, C McPherson, C Rackham; *Oshawa Clinic, Oshawa, ON:* A Bakbak, S Baghiana, K Gibney, L Swailes; *Q&T Research Outaouais Incorporated, Gatineau, QC:* P Nault, K Audet, C Roy, E St-Amour, I Tremblay; *Viacar Recherche Clinique Inc., Greenfield Park, QC:* R Chehayeb, C Lepage; Vizel Cardiac Research, Cambridge, ON: S Vizel, B Fox;

China

*National coordinator:* L Jiang; *Collaborators: Affiliated Zhongshan Hospital of Dalian University, Department of Cardiology:* Q Yu, L Chi, F Liu; *Baotou Central Hospital, Department of Cardiology:* R Zhao, X Li, Y Qian, J Wang; *Beijing Chao-Yang Hospital, Capital Medical University, Department of Cardiology:* X Yang, M Chen, X Lin, F Zhang; *Beijing Liangxiang Hospital of Fangshan District, Department of Cardiology:* X Fang, Q Yu, W Su, X Zhu; *Changsha Central Hospital, Department of Cardiology:* H Dai, L Huang, G Ye, Y Zhao; *China-Japan Union Hospital of Jilin University, Department of Cardiology:* P Yang, M He, B Li; *Dalian Municipal Central Hospital, Division of Cardiology:* Y Zhi, L Sun, L Xiao, Y Yuan; *Fenyang Hospital of Shanxi Province, Department of Cardiology:* R Guo, Q Wang, Y Wang; *Fuwai Hospital, 25th Ward:* J Li, Y Gao, Y Guo, L Li, M Zhai, L Zhang; *Fuwai Hospital, Department of Cardiology:* Y Yang, J Song; *Fuwai Hospital, Department of Heart Surgery:* Z Zheng, X Wang, Y Zhao, C Zhao; *Houma City People Hospital, Department of Cardiology:* Z Wang, C Li; *Hulun Buir People's Hospital, Department of Geriatrics:* Z Cui, X Zhang, L Zhao*; Inner Mongolia Autonomous Region of Traditional Chinese-Mongolian Medicine Hospital, ICU:* H Su, X Huang, R Zhang; *Inner Mongolia Baogang Hospital, Department of Cardiology:* Z Ge, D Liu, Q Liu; *Inner Mongolia People's Hospital, Department of Cardiology:* Y Han, W He, Y Zhang; *Jilin City Central Hospital, Department of Cardiology:* D Qian, L Liu, X Yao; *Jilin Province People's Hospital, Department of Endocrinology:* Y Du, L Song; *Liaoning Shenyang Sujiatun District Central Hospital, Department of Cardiology:* H Che, D Li, C Sun; *Peking Union Medical College Hospital, Department of Cardiology:* S Zhang, H Bai, W Chen, Y Han, Z Liu, J Yang; *Peking University Third Hospital, Department of Cardiology:* Z Li, J Bai, F Wang; *Qingdao Municipal Hospital, Department of Cardiology:* X Wang, C Xing, Y Yao; *Qingdao Fuwai Hospital, Chinese Academy of Medical Science, Department of Cardiology:* X Jiang, Y Yang, Y Dong, G Wu, B Zhang; *Shanghai Changzheng Hospital, Department of Cardiology:* Z Wu, W Chen, Y Chu, X Gu; *Shanxi Cardiovascular Hospital, Department of Cardiology:* B Li, J Wang, X Chai, H Zhang; *Shanxi Provincial People's Hospital, Department of Cardiology:* H Zhang, S Sun, L Tong; *Shengjing Hospital of China Medical University, Department of Cardiology:* X Li, S Ma, H Li, J Liu, X Liu, J Shi; *Shuangshan Hospital of Anshan, Department of Cardiology:* R Xiao, X Li, R Wu; *Suzhou Kowloon Hospital，Shanghai Jiaotong University Medical School, Department of Cardiology:* F Liu, X Meng, B Shao, T Zhang; *Tai Yuan City Centre Hospital, Department of Cardiology:* X Chen, T Feng, L Huo, X Shang; *The Affiliated Hospital of Medical College Qingdao University, Department of Emergency Cardiology:* C Zhou, M Guo, P Li; *The Affiliated Hospital of Medical College Qingdao University, Department of Emergency Neurology:* H Pei, D Han, H Li; *The Affiliated Hospital of Xuzhou Medical College, Department of Cardiology:* D Li, C Cheng, M Huang, W Wu, T Xu; *The Affiliated People's Hospital of Inner Mongolia Medical College, Cadre Ward:* J Liu, J Xia; *The Central Hospital of Wuhan, Department of Endocrinology:* S Zhao, C Cheng, S Ding, L Guo, N Li; *The Fifth People's Hospital of Shenyang, Department of Cardiology:* Q Diao, Z Liu, H Wang; *The First Affiliated Hospital of China Medical University, Department of Cardiology:* G Qi, Z Jia, Y Meng, C Wu; *The First Affiliated Hospital of China Medical University, Department of Cardiology, Anshan Hospital:* B Liu, X Bian; *The First Affiliated Hospital of Harbin Medical University, Department of Cardiology:* W Li, M He, J Jing, B Liu; *The First Affiliated Hospital of Harbin Medical University, Department of Neurology:* L Zhang, Y Sun, X Wang, S Wu, Y Xu; *The First Affiliated Hospital of Xinxiang Medical University, Department of Cardiology:* F Lv, C Guo, J Long, Y Wang; *The First Affiliated Hospital of Zhengzhou University, Department of Cardiology:* Z Huang, X Fu, H Yao, L Zhang; *The First Hospital of Jilin University, Department of Cardiology:* Y Zheng, X Li, C Liu, Q Tong; *The First Hospital of Jilin University, Department of Endocrinology:* Q Liu, G Wang, Y Cheng, X Gang, W Guo, G He; *The First Hospital of Shanxi Medical University, Department of Cardiology:* Q Han, H Bian, L Duan, C Jin, X Wei; *The First People's Hospital of Shenyang, Department of Cardiology:* F Feng, W Xing, J Xu; *The Fourth Affiliated Hospital of China Medical University, Department of Cardiology:* Y Jin, Y Lin, X Zhou; *The Fourth People's Hospital of Shenyang, Department of Cardiology:* Y Li, X Guan, X Zhou; *The General Hospital Of AISCO, Department of Cardiology:* X Liu, H Liu, L Liu; *The General Hospital of FAW, Department of Cardiology:* H Pan, X Wang, S Zhang; *The General Hospital of Shenyang Military Command, Department of Cardiology:* Y Han, P Fan, J Li, R Ma, G Wang, P Wang; *The General Hospital of Xuzhou Mining Group, Department of Cardiology:* W Wu, L Li; *The People's Hospital of Liaoning Province, Department of Cardiology:* Z Li, R Cui; *The Second Affiliated Hospital of Baotou Medical College, Department of Cardiology:* G Sun, F Wang, R Xie; *The Second Affiliated Hospital of Dalian Medical University, Cardiovascular Department:* P Qu, G Li, J Mei, L Wang, Q Yang, C Zhang; *The Second Affiliated Hospital of Harbin Medical University, Department of Cardiology:* B Yu, W Cao, W Du, Y Luan; *The Second Affiliated Hospital of Harbin Medical University, Department of Neurology:* W Wang, Y Zhu, H Jiao, Y Qu, Y Zhu; *The Second Hospital of Shanxi Medical University, Department of Cardiology:* Z Yang, N Du, J Li, B Liang, W Wu, H Yang; *The Second Hospital of Tianjin Medical University, Department of Neurology:* X Li, D Wang, P Zhao; *The Third People's Hospital of Dalian, Department of Cardiology:* N Li, X Liu, B Xu, D Zong; *The Third People’s Hospital of Xuzhou, Department of Cardiology:* L Wang, X Tang, C Zong; *The Third Xiangya Hospital of Central South University, Department of Endocrinology:* Z Mo, P Jin, J Xiong; *Tianjin Chest Hospital, Department of Cardiology:* H Cong, X Guo, R Liang, J Zhou; *Tianjin Fourth Center Hospital, Department of Cardiology:* H Zhang, Y Liu, Z Sun; *Tianjin Medical University General Hospital, Department of Cardiology:* Y Sun, Z Wan, B Bian, Y Li, W Zhang; *Tianjin Union Medicine Centre, Department of Cardiology:* Z Yao, Y Liu, R Wang; *Tianjin Union Medicine Centre, Department of Neurology:* C Zhang, G Chen, C Ma; *Tongji Hospital, Department of Cardiology, Tongji Medical College:* D Wang, J Jiang, L Ni, H Yan; *Wuhan Asia Heart Hospital, Department of Cardiology:* X Su, J Cai, J Ma, R Zhong; *Wuhan Puai Hospital, Department of Cardiology:* Y Gu, L Hu, X Wu; *Wuxi People's Hospital, Department of Cardiology:* X Wu, Z Yang, M Chen, J Feng; *Xiangtan City Central Hospital, Department of Cardiology:* H Huang, F Ouyang, Z Sun, Y Zhou; *Xiangya Hospital Central-South University, Department of Cardiology:* T Yang, X Deng, L Peng, Y Zhao; *Xinxiang Central Hospital, Department of Cardiology:* L Liu, S Su; *Xuzhou Central Hospital, Department of Cardiology:* Q Fu, L Wang, X Zong; *Xuzhou N^o^ 1 People's Hospital, Department of Cardiology:* H Zhang, L Li, X Liu, Y Shang; *Zhengzhou Central Hospital, Cardiovascular Medicine Department:* L Zhang, H Li, H Sun; *Zhu Zhou N^o^ 1 Hospital, Department of Cardiology:* S Guo, Y He, L Cha, Y Lu;

Denmark

*National coordinator***:** K Egstrup; *Collaborators: Aalborg Hospital:* E Berg Schmidt, P Dinesen, A Gammelmark, M Nielsen, T Rix, H Vadmann, K Andersen, B Christensen, L Hessing Kobbelgaard, B Mikkelsen, M Storgaard; *Esbjerg Hospital:* O Nyvad, A Rohold, K Thomsen, J Hummelshoj, A Svenningsen, L Tanggaard; *Gentofte Sygehus, Hellerup:* G Gislason, C Torp Pedersen, G von Jessen, J Larsen, J Sandberg Madsen; *Glostrup Hospital:* H Iversen, C Nielsen, J Obionu, S Simonsen, M Kjaergaard Danö, P Hornslet, T Veng-Olsen; *Herning Hospital:* O May, L Madsen, M Engbjerg Andersen, L Jensen, V Lynggaard, S Søndergaard, S Vester; *Holbæk Hospital:* N Roseva-Nielsen, V Sørensen, K Skjødeberg Christensen, M Bang Hansen, L Jensen, H Mollerup, S Voigt; *Kolding Hospital:* J Jepsen, J Gesla, L Johansen, E Zeuthen, B Bjerge Kaspersen, B Felthaus, M Løkke, L Holm Pedersen, A Schrader, L Schmidt Thomsen; *Kolesterollaboratoriet, Gentofte:* S Stender, T Brink-Kjaer, H Jonsson; *Næstved Hospital:* R Sykulski, J Thorsen, P Osterby Elin, B Stage Jensen; *Nordsjællands Hospital, Helsingor:* N Ralfkier, H Gottschalck, S Bloksgaard Nilesen; *Odense Universitets Hospital:* M Lytken Larsen, H Mickley, S Hosbond, L Saaby, M Ronn, I Rosenlund; *Rigshospitalet, Copenhagen:* P Clemmensen, P Grande, L Køber, H Andersson, S Wiberg, K Graversen, L Hedgaard, M Tarras Wahlberg; *Roskilde Hospital:* T Melchior, C Larsen, S Heinsvig, I Larsen, V Perret-Gentil; *Silkeborg Hospital:* L Frost, A Christensen, H Arp, M Mortensen, A Odgaard; *Skejby Hospital, Århus:* H Wiggers, S Poulsen, K Serup-Hansen, G Udsen; *Slagelse Hospital:* J Lomholt, H Mollerup, K Niemann; *Steno Diabetes Center, Gentofte:* M Ridderstråle, L Tarnow, T Boesgaard, T Hansen, N Safai, M Andersen, S Hansen, M Pedersen; *Svendborg Hospital:* K Egstrup, S Auscher, A Osmanagic, H Sheta, K Vinter, L Hindsgaul, M Lundgaard, L Moltrup; *Viborg Hospital:* I Klausen, B Haastrup, B Hedegaard, S Gudmundsdottir;

Finland

*National coordinator***:** A Kesäniemi; *Collaborators: Etelä-Karjalan Keskussairaala, Lappeenranta:* E Hussi, S Valpas; *FinnMedi Oy, Tampere:* J Taurio, A Airaksinen, S Luukkonen, S Uusitalo; *Geri-Med Oy, Helsinki:* T Strandberg, E Ronkainen, C Sarti, R Tilvis, M Aaltonen, E Landström, A Punkka; *Kuopion Liikuntalääketieteen Tutkimuslaitos, Kuopio:* T Lakka, K Savonen, H Kastarinen, N Koskinen; *Kuusankosken Terveysasema, Kuusankoski:* M-L Tuominen, A Haaraoja; *Lapin Keskussairaala, Rovaniemi:* J Laukkanen, A Hadjikov, S Hellsten, P Hiltunen; *Menoa Oy, Kinkomaa:* M Perhonen, M Valtonen, M Moilanen, M Varakas; *OYS, Oulo:* A Kesäniemi, E Eloranta, O Ukkola, P, Ojala, L Ukkola; *Pohjois-Karjala Projektisäätiö, Joensuu:* S Pihlman, M Mononen, K Hyttinen, S Lipponen; *Seinäjoen Lääkäritalo, Seinäjoki:* M Kotila, A Pöllänen, A Rajala; *Turun Yliopistollinen Sairaala, Turku:* I Kantola, T Kiviniemi, M Strandberg, J Raali, E Roine;

Germany

*National Coordinator:* C Wanner; *Collaborators: Ambulantes Herzzentrum, Kassel:* K-F Appel, S Appel, A Utech, P Becker, S Chmilewski, J Kuehnert, I Pietsch, A Reinemann, S Werner; *Cardiopraxis, Mainz:* G Mentz, M Drexler, I Müller-Wittlich, A Drexler, S Hobrack, K Tajouaout; *Charité Campus Virchow-Klinikum, Berlin:* H-D Düngen, T Bekfani, G Cherian, M Fritschka, L Musial-Bright, T Trippel, V Tscholl, A Baltic, S Inkrot, A Maiwald, A Pinta, M Sacirovic, Y Saewe, R Stolz; *Comprehensive Heart Failure Center [CHFC] and Department of Internal Medicine I, University Hospital and University of Würzburg, Würzburg:* CE Angermann, G Ertl, S Brenner, K Boelmans, M Breunig, F Hammer, U Hofmann, P Jung, D Menhofer, B Meyer, C Reichert, C Wanner, J Judex, A Knoppe, C Richter; *DRK Kliniken, Berlin:* H Voehringer, E Lianopoulos, C Opitz, M Buchholz, S Gebhardt, S Helms; *Forschungszentrum Ruhr, Witten:* T Horacek, G Kahrmann, O Stobbe, P Fink, A Günesli, J Richtstein, K Wilke; *Gefäß Zentrum Universitaetsklinik, Dresden:* N Weiss, N Jabs, A Mahlmann, S Werth, S Brilloff, M Dechert, E Festerling, M Leistner, B Sehr, I Weise; *Gemeinschaftspraxis Dr. Haggenmiller und Dr. Jeserich, Nuernberg:* M Jeserich, S Haggenmiller, S Kimmel, H-O Schoengart, M Cakir, G Eichinger, M Rupprecht; *Juedisches Krankenhaus, Berlin:* K Graf, R Thieme, E Tummos, J Ausner, L Fischer; *Kardiologie Universitaet, Magdeburg:* R Braun-Dullaeus, H Bönigk, S Meißler, A Schmeißer, H Schulz, S Uslar, D Weigt, R Gebauer, S Roeder, K Schäfer; *Kardiologische Geimeinschaftspraxis, München:* S Silber, M Basler, C Matt, P Styllou, B Bosnjak, M Huth, A Schmid, C Senger; *Kardiologische Gemeinschaftspraxis, Würzburg:* M Camerer, H Drösch, H Strömer, J Heid, R Wilsch; *Klinik am See, Ruedersdorf:* H Völler, A Jawari, S Stiehl, A Salzwedel, K Stolze; *Kerkhoff Klinik, Bad Nauheim:* V Mitrovic, L Gaede, A Peil, M Shaker; *Klinikum Bielefeld:* C Stellbrink, C Drephal, B Elberg, J Junge, E Stellbrink, T Weber, B Brettschneider, C Gruhne, M Iselt, J Kube, U Lehmann, C Potthast, S Watson; *Klinikum Coburg:* J Brachmann, M Held, C Mahnkopf, A Saleh, A Sallam, B Schertel-Gruenler, S Schnupp, U Goebel, S Rube, K Truthan; *Klinikum Frankfurt Hoechst:* C Kadel, K Lahiri, H Moellinger, M Pagitz, J Reusch, A Stadler, N Zulauf, V Anuschek, S Beißner, M Buerger, M Hagmanns, E Hickmann, C Klinger, G Rahn, J Schemann, E Tu, C Wölfl; *Klinikum Fulda:* V Schaechinger, T Pluecker, G Strupp, T Trepels, W Wahl, C Roemmelt, S Fritsch; *Klinikum Leverkusen:* P Schwimmbeck, A Fahrig, M Hautmann, A Öner, B Weidmann, I Wenzel; *Klinikum Universität München - Campus Innenstadt:* U Hofmann, M Czihal, K Hausleiter, K Kress, R Kreuzpointner, P Kuhlencordt, S Rieber, J Nuerbchen, S Roth-Zetzsche; *Klinikum Universität München - Grosshadern:* S Kääb, W Franz, C Feldmann, U Grabmaier, M Sinner, A Bongartz, C Gross, B Halter, J Sakic; *Medizinische Hochschule, Hannover:* J Bauersachs, U Bavendiek, J Pirr, K Sonnenschein, K Hohenleitner-Lührßen, A Juergens, N Schaefer; *Praxis fuer Kardiologie Dr. Bosiljanoff, München:* P Bosiljanoff, G Betzl, E Bosiljanoff, J Feger, A Kinateder; *Robert Bosch Krankenhaus, Stuttgart:* U Sechtem, S Egenrieder, A Karagianni, T Schäufele, M Voehringer, S Gruensfelder, L Hoffmann, I Wenzelburger; *SLK-Kliniken Heilbronn, Bad Friedrichshall:* T Dengler, C Loges, C Neatu, C Lindner, C Pfau; *St. Johannes Hospital, Dortmund:* H Heuer, H Bourhaial, I Dulea, B Elberg, O Guerocak, S Halberstadt, E Kemala, J Peterek, H Philips, U Dieckheuer, K Euler, B Laschewski, M Maas, J Peda; *Studienzentrum Prof. Hanefeld, Dresden:* F Schaper, E Henkel, M Teige, C Schrapel, K Waetzold; *Universität Magdeburg Lipidambulanz:* B Isermann, S Westphal, K Borucki, M Schulze, K West; *Universitäts-Herzzentrum Freiburg-Bad Krozingen:* D Trenk, W Hochholzer, S Leggewie, C Stratz, A Schiebeling-Romer, M Siefer; *Universitätsklinikum, Bonn:* N Werner, R Schueler, A Sedaghat, J-M Sinning, K Twelker, U Jones, M Lauterborn, M Lennarz, S Lubnau, A Meier, T Streuber-Bouhksas; *Universitätsklinikum, Hamburg:* S Blankenberg, M Adam, C Alternburg, M Huemmelgen, A Jagodzinski, M Karaks, K Koopmann, S Schäfer, H Schüler, K Sydow, C Thomas, E Tigges, I De Boer, M Hermes, J Nebel, C Schlesner, J Schlüter, D Sprechert, S Zbik; *Universitätsklinikum Münster Kardiologie:* J Waltenberger, D Fisscher, C Schulz; *Universitätsklinikum Münster Lipidambulanz:* B Otte, L Centofante, R Kremerskothen, J Beilker, S Müller, E Schlosser; *Universitätsklinikum Regensburg:* C Birner, A Luchner, J Egresits, C Jungbauer, M Resch, P Schmid, M Buesing, C Liebl, S Sülflow; *Universitätsklinikum Schleswig-Holstein, Luebeck:* J Weil, B Brueggemann, T Graf, C Moeller, M Miodek; *Vivantes Klinikum am Urban, Berlin:* H Ince, D Andresen, A Seidel, C Sprenger, Y Stoeckicht, S Ziefle, S Forster, J Ort, S Szczesnak;

Italy

*National coordinator:* AP Maggioni; Collaborators: *Albano Laziale, Ospedali Riuniti Albano-Genzano:* P Midi, A Felici, F Caranzetti, M Miccoli, L Tomassini; *Aosta, Ospedale Generale Regionale-PO U.* Parini: M Sicuro, C Aillon, C Gianonatti, C Baré, P Donà; Bari, Ospedale San Paolo: P Caldarola, M Resta, M Ruggiero, M Galietti; *Bologna, Ospedale Maggiore:* G Di Pasquale, E Filippini, L Riva, S Zagnoni; *Casarano, Presidio Ospedaliero F. Ferrari:* G Piccinni, C Perrone, A Aloisi; *Cortona, Ospedale Valdichiana Santa Margherita:* F Cosmi, B Mariottoni, B Tarquini; *Cremona, Ospedale di Cremona:* S Frattini, S Pirelli, G Paradiso, S Signore; *Firenze, Ospedale San Giovanni di Dio:* C Baggiore, C Crescenti, A Leopardi, S Benoni, P Ferruzzi, P Pini; *Foggia, Ospedali Riuniti:* M Di Biase, C D'Antuono, R Ieva, I Monaco, D Montrone, S Musio; *Gubbio, Ospedale Gubbio-Gualdo* *Tadino:* S Mandorla, M Buccolieri, E Capponi, S Martinelli, N Piccioni, O Regni, A Iaquaniello, A Malvestiti, S Pieroni Minciaroli; *Isernia, Ospedale F. Veneziale:* C Olivieri, R Chiodi, A Masciotra; *Legnano, Ospedale Civile:* F Poletti, S De Servi, S Affinito, A Di Donato, S Messina, C Stefanin; *Lido di Camaiore, Nuovo Ospedale Versilia:* G Casolo, L Robiglio, F Vivaldi, A Buono, C Urbani; *Milano, Ospedale Niguarda:* A Alberti, E Giagnoni, T Pupilella, A Biondi, A Di Donato, A Lazzari, S Messina; *Orbassano, Ospedale San Luigi Gonzaga:* L Montagna, C Chirio, I Salvetti, M Perrelli; *Palmanova, Ospedale di Palmanova:* MG Baldin, R Cesanelli, S Boccati, G Durì; *Pavia, IRCCS -Fondazione Salvatore Maugeri:* S Priori, M Ceresa, M Zambelli, A Biondi, A Di Donato, S Messina, G Savino; *Rimini, Ospedale Infermi:* G Piovaccari, D Grosseto, P Testa, P Gaviani, A Girardi, I Manzo, G Serroni; *San Felice A Cancello, Ospedale Ave Gratia Plena:* C De Matteis, U Campidonico, C Crisci, M Falco, C Di Matteo, I Manzo; *Santa Maria Capua Vetere, Ospedale San Giuseppe e Melorio:* L Fattore, G Morello, C Nave, C Di Matteo, I Manzo; *Sarzana, Ospedale San Bartolomeo:* R Petacchi, D Bertoli, G Filorizzo, A Buono, C Urbani; *Scorrano, Ospedale Ignazio Veris Delli Ponti:* O De Donno, E De Lorenzi, L Urso, A Aloisi, A Lecci; *Sondrio, Ospedale Civile:* G Cucchi, E Gianatti; *Terni, Azienda USL Umbria 2:* G Proietti, M Bernardinangeli, G Proietti, S Serani; *Udine, AOU Santa Maria Della Misericordia:* G Morocutti, T Bisceglia, C Fresco, V Andrioli, V Biundo; *Veruno, Fondazione Salvatore Maugeri:* P Giannuzzi (deceased), M Gattone, V Bolzani, M Di Ruocco, A Biondi, A Di Donato, S Messina, B Temporelli;

Japan

*National coordinators****:*** S Goto, T Teramoto; Collaborators: *Institute of Brain and Blood Vessels Mihara Hospital:* B Mihara; *National Hospital Organization Kyushu Medical Center:* Y Okada;

Norway

*National coordinator****:*** O Nygard; *Collaborators: Førde sentralsjukehus, Førde:* T Vingsnes, H Sirnes, K Solheim, R Tilseth, M Vestre, A Bjorkas, I Vassbotten; *Haugesund Hjertepraksis, Haugesund:* R Rød, R Stodle; *Haukeland Universitetssykehus, Bergen:* O Nygård, C Berge, H Schartum Hansen, A Kask, K Løland, G Svingen, N Tuseth, V Vavik, E Wilberg Rebnord, B Gjellefall, S Hovland, S Nordgaard Thorsen; *MEDI3 Ålesund, Ålesund:* T Kjærnli, O Erstad, K Grödal, S Nybø, C Royset, S Stadsnes; *Nordland Hjertesenter AS, Bodø:* A Hovland, K Lappegard, J Sandvik, H Carlsen, T Enebakk, H Thunhaug; *Norsk Helseklinikk, Lierskogen:* L Solnør, P Holmstrom; *Skedsmo Medisinske Senter AS, Skedsmokorset:* K Risberg, H Hansen; *St. Olavs Hospital, Trondheim:* B Kulseng, K Lauglo, H Tevik Bjøru, T Langeng, S Salater; *Stavanger Helseforskning AS, Stavanger:* P Scott Munk, E Singsaas, A-G Larsen, S Moen, J Nilsen; *Sykehuset Innlandet HF, Hamar:* K Andersen, T Larsen, E Turkerud Söby; *Sykehuset Innlandet HF, Kongsvinger:* J Sparby, E Werenskjold; *Sykehuset Innlandet HF, Lillehammer:* M Grundtvig, M German, G Szacinski; *Sykehuset Telemark, Skien:* J Hysing, J Thalamus, E Flagstad, H Rosland; *Ullevål Universitetssykehus HF, Oslo;* T Pedersen, T Klemsdal, L Bergengen, R Kleve; *UNN Hjerte/Kar Poliklinikk, Tromsö:* A Skogsholm, K Larsby, I Holde, R Jonassen, M Nilsen; *Vestfold Hjertesenter AS, Sandefjord:* J Berg-Johansen, H Tisthammer Antonsen;

Sweden

*National coordinator***:** L Jonasson; *Collaborators: Capio Lund, Lund:* C Lindholm, J Thulin, E Assarsson, M Broberg; *Centralsjukhuset Kristianstad, Kristianstad*: I Torstensson, I Lager, K Hårsmar, A Knutsson; *City Heart, Stockholm:* L Hjelmaeus, R Zlatewa, E Lindemann, I McLain; *Dalecarlia Clinical RC, Rättvik:* H Larnefeldt, M Eld, M Bjorkman-Larnefeldt: *Halmstads Sjukhus, Halmstad:* P Hårdhammar, P Johansson, A Karlsson, M Lingman, M Löfgren, A Tabandeh, L Andersson, C Palm; *Hässleholms Sjukhus, Hässleholm:* I Timberg, M Stjernberg, P Wikström; *Karolinska Universitetssjukhuset, Stockholm:* C Bergmark, O Thott, U Hedin, C Montan, O Nilsson, M Lenquist; *Linköpings Universitetssjukhus, Linköping:* L Jonasson, L Nilsson, P Wodlin, M Borjesson, A Raschberger; *Ljungby Lasarett, Ljungby:* L Ekholm, K-A Svensson, A Ågårdh, L Algotsson, M-L Bergstrom;*Mölndals Sjukhus, Mölndal:* F Randers, L Klintberg, U Axelsson, P-Å Moström, G Mourtzinis, P Paren, B Persson, M Risenfors, J Moodh, M Mossmark, L Ohrtgren; *Motala Lasarett, Motala:* P Ahlström, Å Törnqvist, U Rosenqvist, M Grandas, G Karlsson; *Norrlands Universitetssjukhus, Umeå:* P Ottander, A Eriksson, M Backlund, M Johansson, C Sundholm; *Öbackakliniken, Härnösand:* A Kempe, S Salomonsson, J Larsson, H Andersson, K Forsberg, A Sjodin; *Oskarshamns Sjukhus, Oskarshamn:* U Mathiesen, M Carlsson, E Keppel, K Fehling, U Robertsson; *Prim Site, Örebro:* A Weiderman, E Jasinska, M Lundvall, K Eriksson, J Kjellgerg Eriksson, U Larsson; *Skellefteå Lasarett, Skellefteå:* J-H Jansson, K Boman, M Johansson, L Lundmark, B Norrfors; *Stockholm Heart Center, Stockholm:* A Ohlsson, L Bastani, T Delgado, S Gunvarsdotter, P Lof, L Persson, K Skoglund; *Växjö Sjukhus, Växjö:* P Vasko, G Anderson, O Bergström, T Nyström, I Uggeldahl;

United Kingdom

*National coordinator:* L Bowman; *Collaborators: Aberdeen Royal Infirmary, Aberdeen:* J Webster, J Henderson, V Herd, E Wilson; *Addenbrooke's Hospital, Cambridge:* F Mir, S Blackwood (monitor), M Watts; *Barnsley District General Hospital, Barnsley:* W Khan, N Tahir, K Elliot, J Lichfield, H Marsh, M Reid; *Bedford Hospital, Bedford:* I Cooper, A Gallivan; *Birmingham Heartlands Hospital, Birmingham:* A Jones, L Andrews, C Jewkes; *Bradford Royal Infirmary, Bradford:* S Lindsay, K Rees, A Wilson (monitor); *Bristol Royal Infirmary, Bristol:* G Bayly, J Chambers (monitor), S George, M Halestrap; *Colwyn Bay Community Hospital, Colwyn Bay:* C Bellamy, S Evans, J James, E Pritchard, J Stockport, S Wynne; *Derriford Hospital, Plymouth:* J Fulton, J Simmonds, C Stewart, C West; *Dewsbury District Hospital, West Yorkshire:* H Chidambara, T Msimanga, B Moore, G Roberts; *Dorset County Hospital, Dorchester:* T Edwards, S Breakspear, N Fleming; *Edinburgh Royal Infirmary, Edinburgh:* D Newby, E Fraser, L Marshall, H Nailon; *Guy's Hospital, London:* J Chambers, D Parkin; *Hillingdon Hospital, Uxbridge:* M Edwards, N Mahabir; *Huddersfield Royal Infirmary, Huddersfield:* H Griffiths, K Mitchell, D Appleyard, S Farr; *King's Mill Hospital, Sutton-in-Ashfield:* R Lloyd-Mostyn, S Hardingham, T Sewell; *Lister Hospital, Stevenage:* M Lynch, W Burog, M Dhaliwal, C Mfuko; *Luton and Dunstable Hospital, Luton:* C Travill, S Gent, B Norris; *Macclesfield Hospital, Macclesfield:* R Edgell, T Lake, A Taylor Bennett (monitor); *Manor Hospital, Walsall:* A Hartland, E Walton (monitor); *Memorial Hospital, Darlington:* J Murphy, G Brennan, P Cawley, L Dixon, E Rees; *Musgrove Park Hospital, Taunton:* R Andrews, T Brownlow, S Crouch, H Mills, M Nixon, N Salter; *Ninewells Hospital, Dundee:* S Pringle, H Waldie; *North Manchester General Hospital, Manchester:* J Swan, D McSorland; *North Tyneside General Hospital, North Shields:* R Curless, M Armstrong, C Ashbrook-Raby, D Bunn, R Gour, C Herriott, J James, C Robson, C Tanney, A Taylor Bennett (monitor); *Northampton General Hospital, Northampton:* P Davey, L Campey, K Smith, E Tanqueray; *Pinderfields Hospital, Wakefield:* A Munir, O Pereira, M Khalifa, B Moore; *Princess Royal Hospital, Telford:* N Capps, D Donaldson (monitor), C Miller, L Tonks; *Queen's Hospital, Burton-upon-Trent:* T Reynolds, P Basvi, J Reynolds, L Wilcox; *Queen's Medical Centre, Nottingham:* P Mansell, G Babington, E Barnes, S Beck, S Craig, L Patterson, A Selby, C Woodford; *Richard Doll Building, Oxford:* L Bowman, M Landray, J Armitage, H Watkins, S Beebe, K Bowsher Brown, J James, H Lochhead, J Robertson, V Toghill, L Wright, L Young; *Rotherham District General Hospital, Rotherham:* R Muthusamy, M Lawan, C Weston; *Royal Berkshire Hospital, Reading:* W Orr, J Foxton, S Hallett, P Hilltout, L Jones, J King; *Royal Blackburn Hospital, Blackburn:* S Ramtoola, Y Grimes; *Royal Bolton Hospital, Bolton:* A Hutchesson, J Cummings, K Morris; *Royal Cornwall Hospital, Truro:* S Fleming, K Ludlow, M Parrett, S Pellow, L Quinn; *Royal Devon & Exeter Hospital, Exeter:* M James, E Green, S Keenan; *Royal United Hospital, Bath:* J Reckless, A Robinson, G Andrews, A McLenaghan; *Royal Victoria Hospital, Newcastle upon Tyne:* I ul Haq, C Albers; *Russells Hall Hospital, Dudley:* M Labib, E Higginson; *Salford Royal Hospital, Salford;* A Fitchet, E Darrel-Asherel, J Green, M Healey, K Morris, D Sexton; *Sandwell General Hospital, West Bromwich:* E Hughes, J Chackathayil, S Willetts; *Southampton General Hospital, Southampton:* C Shearman, A Lewis, M Pasinabo, C Trevithick, D Tyler, B Watkins; *Southmead Hospital, Bristol:* M Papouchado, G Andrews, W Bertram, E Binley, S Hierons, S Kandola, C Mann, K Whitney; *St Helier Hospital, Carshalton:* H Wilcox, A Bibi, J Fuller, S Jackson; *St Mary's Hospital, Portsmouth:* P Kalra, S Howe, K Hudson, A Suttling, C Turner; *St Richard's Hospital, Chichester:* Y Wong, L Clayton-Evans, S Moore, S Stearn; *Stepping Hill Hospital, Stockport:* P Lewis, H Cochrane, J Curtis, M Holland; *Sunderland Royal Hospital, Sunderland:* S Junejo, E Dungca, T Robson, A Smith, A Taylor Bennett (monitor); *Torbay Hospital, Torquay:* C Carey, L Felmeden, A Summerhayes, J Sutton; *University Hospital of North Staffordshire, Stoke-on-Trent:* R Butler, J Creamer, J Bellaby, K Castro-Foskett, M Griffiths, J Machin, I Massey, E Sellars, J Wain*; University Hospital of Wales, Cardiff:* I McDowell, L Davies, M Davies, H Dyer, M Odam, A Waters; *University Hospital, Coventry:* M Been, V Ansell, A Campbell, D Davies, B De Burca, J Jones, A Musa; *Victoria Hospital, Blackpool:* D Roberts, R Brady, C Dickinson, L Lane, S Pickervance; *Victoria Hospital, Kirkcaldy:* M Francis, V Bryson; *Watford General Hospital, Watford:* M Clements, L Ashton, A George, K Markwell, E Walker; *West Cumberland Hospital, Whitehaven:* O Orugun, U Poultney; *Whipps Cross University Hospital, London:* F Lie, A Taneja, B Badal, V Conteh, M Jones, M Montemayor; *Worcestershire Royal Hospital, Worcester:* J Trevelyan, E Byng-Hollander, A Doughty; *Worthing Hospital, Worthing:* M Signy, A Dunne, H Fox, S Moore, S Stearn, K Wheatley; *Wycombe General Hospital, Wycombe:* S Price, N Mahabir; *Wythenshawe Hospital, Manchester:* S Ray, S Golledge, M Holland, M Murmu, A Nicholas; *Yeovil District Hospital, Yeovil:* G Brigden, J Board, C Buckley, C Vickers;

United States of America

*National coordinator:* CP Cannon; *Collaborators: Acadia Clinical Research LLC, Bangor, ME:* M Albert, G Baillargeon, D Harman; *Advanced Heart Care, LLC, Bridgewater, NJ:* S Mahal, J Kaur, S Padkowsky, M Walker, S Yandamuri; *Advanced Neurology Specialists, Great Falls, MT:* D Dietrich, L Armstrong, R Brown, M Casey, V Schaefer; *Albuquerque Clinical Trials, Inc., Albuquerque NM:* E Bretton, D Hsi, J Kovach, J Troy; *Asheville Cardiology Associates, Asheville, NC:* B Asbill, L Brown, T Cauthren, A Hull, O Lim, J Tompkins, J Vaughn; *Associated Cardiovascular Consultants, Voorhees, NJ:* R Perlman, D Connors, D Hoopes, D Palazzo, A Prosser, M Serrano-Rawls; *Associated Research Partners LLC, Jonesboro, AR:* B Tedder, E Johnson, T Pearson, K Rubino, P Williams; *Atlanta Heart Specialists, LLC, Cumming, GA:* N Singh, M Brown, S Dubal, E Hall, D Logwood, U Mazahir, K Raynes; *Austin Heart, PLLC, Austin, TX:* R Gammon, J Hatch, P Mock, N Tilton; *Austin Heart, PLLC, Jonesboro, AR:* W Abide, Jr., D Gudeman, S Minor, T Shipwash; *Aventura Heart Center, Aventura, FL:* D Korn, A Korn; *Awasty Research Network, LLC, Marion, OH:* V Awasty, E Baldwin, G Hunt, V Kaiser, C McMurray; *Bay Area Cardiology Associates P.A, Brandon, FL:* T Khan, J Al-Jumaily, T Foster, V Holbrook; *Baylor College of Medicine, Houston, TX:* V Nambi, C Ballantyne, M Jackson, P Jones, B Morris, M Techmanski, A Tran; *Beverly Hills Cardiology, Los Angeles, CA:* S Eshaghian, H Mirshkarlo; *Black Hills Cardiovascular Research, Rapid City, SD:* A Zineldine, J Bies, D Hockett, L Kimball; *Boice Willis Clinic, Rocky Mount, NC:* S Varma, S Barkley-Daughtry, S Collins, S Evans-Gay, L Martin, A McKinley, L Murray, L Noel, S Prasada, R Robinson; *Brigham and Women's Hospital, Boston, MA:* CP Cannon, J Andreo, S Bansilal, E Bohula May, M Cavender, J Cyr, N Desai, C Fanola, N Fantony, R Giugliano, J Gutierrez, P Kazanjian, J Marti-Bernier, J Mega, R Mesa, M O'Donoghue, B Scirica, M Silverman, D Steen, L Williams; *Bryan Heart, Lincoln, NE:* C Meckel, C Orosco, R Saalfeld, N Thompson, C Wiechert; *Buffalo Medical Group,* *P.C., Williamsville, NY:* L Kozlowski, B Cooke, J Corbelli, A Galla, R Stock; *Capital Cardiology Associates, Troy, NY:* R Benton, A Carroll, C Leeper, E Orvis; *Capital Cardiology Associates, Albany, NY:* P Shah, A Kasson, J Lieberman, A O'Malley, E Orvis; *Cardiology Associates of Bellin Health, Green Bay, WI:* J Rider, B Loomis, M Schantz; *Cardiology Associates of Fairfield County, P.C., Stamford, CT:* M Heiman, K Sadowski, L Scierka, J Sclafani, D Mania, E Del Mastro; *Cardiology Associates of Fairfield County, P.C., Trumbull, CT:* R Jumper, K Bukoski, P Eiben, R Keegan, E Kelley, E Sekerak, J Weisberger, A Serra; *Cardiology Associates of Fairfield County, P.C., Norwalk, CT:* C Augenbraun, S Jumper, A Stuart, A Archer, T Malak, J Velky; *Cardiology Consultants, Pensacola, FL:* R Spencer, B Lane, J Lehmann; *Cardiology Research Associates, Daytona Beach, FL:* D Henderson, L Crandall, A Easterling, A Lizama, D Millard; *Cardiovascular Associates of the Delaware Valley, PA, Elmer, NJ:* M Gelernt, C Billings, D Cockrell, E Anderson; *Cardiovascular Associates of the Delaware Valley, PA, Haddon Heights, NJ:* A Pavlides, M Davis; *Cardiovascular Associates of the Delaware Valley, PA, Sewell, NJ:* D Viswanath, M Kinder, H Jeffers, S Manga, P Shaw; *Cardiovascular Associates of the Southeast, Birmingham, AL:* S Jones, T Stover; *Cardiovascular Associates of the Southeast, Birmingham, AL:* R Reeves, S Frew; *Cardiovascular Institute of the South, Opelousas, LA:* K Veerina, N Domingue, L Huffman, Y Leach, T Rideaux, J Smith, L Soileau; *Cardiovascular Research Foundation of Southern California, Beverly Hills, CA:* R Karlsberg, Bhatia, A Gomez, L Levi, D Lopez; *Cardiovascular Research of Knoxville, Knoxville, TN:* C Treasure II, L Michaelis, M Parker, C Robertson, L Treasure; *CentraCare Heart and Vascular Center at St. Cloud Hospital, St. Cloud, MN:* B Erickson, A Amundson, J Humbert, H Madden; *Charles River Medical Associates, Natick, MA:* V Desai, K Lemmertz, J Zoghbi; *Charlotte Heart Group Research Center, Port Charlotte, FL:* M Malone, K Mullinax, R Schenks; *Chesapeake Cardiovascular Associates, Baltimore, MD:* D Goldscher, M Fisher, J Latteri; *Chesapeake Cardiovascular Associates, Towson, MD:* M Goldstein, H Lutz; *Chesapeake Cardiovascular Associates, Baltimore, MD:* D Peichert, E Haskel, J Powell, C Yashinski; *Clearwater Cardiovascular and Interventional Consultants, Clearwater, FL:* J Amin, D Bashton, S Burns, A Davidson, C DeSousa, C Humberger, R McGee; *Clearwater Cardiovascular and Interventional Consultants, Safety Harbor, FL:* J Zelenka, D Ferguson, C Manuel, J Quinn, J Zelik; *Cleveland Clinic Fairview Cardiovascular Medicine, Fairview Park, OH:* E Nukta, B Bittel, M Dettmer, C Palmer; *Clinical Research Associates, Florence, SC:* W Boulware, L Cooper, R Freeman; *Clinical Trials of America, Shreveport, LA:* W Zhang, K Banks, L Hall, C Hall, K Riser, S Vaz, J Winstead, L Womack; *Clinical Trials of America, Inc, Lenoir, NC:* J Dy, L Fox, E Landers, B Raby, T Whisnant; *Clinical Trials of America, INC, Hickory, NC:* S Isserman, T Annas, K Kirby, J Lail, C Moore, A Waters; *Cohen Medical Associates, Delray Beach, FL:* R Cohen, J Bossaers, L Heaney, A Hislop, L Moreiras, M Ocampo; *Community Clinical Research Center, Anderson, IN:* P Jetty, T Allen, C Custer, S Howard, A Key, S Lipps; *Comprehensive Cardiovascular Medical Group, Bakersfield, CA:* S Banerjee, S Carlos, A Garza, R Sutton; *Dayton Heart Center, Dayton, OH:* J Tobiansky, J Gluck, C Tofstad; *Doylestown Health Cardiology, a Division of Doylestown Health Physicians, Doylestown, PA:* J Kmetzo, J Brown, L Carter, R Riley, P Seger, D Taylor, D Wood; *East Texas Cardiology, PA, Houston, TX:* A Ahmad, M Ahmed, H Ayub, S Contreras, S Iqbal, S Martinez, M Martinez; *Eastern Suffolk Cardiology, Southampton, NY:* S Donahoe, P Dalal, C Defraia, R DeStefano, S Lederman, D Lorme, M Ruhani; *Escondido Cardiology Associates, Inc, Escondido, CA:* R Acheatel, J Biggers; *Florida Hospital, Orlando, FL:* C Kim, D Barnes, K Behm, A Dziekonski, H Karunaratne, C Stastny; *Gemini Scientific, LLC, Madison, WI:* N Bittar, S Lehmann, M Spatola, P Wilson; *Gotham Cardiovascular Research, New York, NY:* C Staniloae, E Homberg-Pinassi; *Grand View-Lehigh Valley Health Services, Buxmont Cardiology Division, Sellersville, PA:* P Hermany, K Batchlett, A Gibson, S Meissner-Dengler; *Green and Seidner Family Practice Associates, Lansdale, PA:* J Rosenfeld, B Madden, M Seidner, K Sosonkin; *Harrisonburg Medical Associates, Harrisonburg, VA:* S Pollock, S Johnson; *Health First Medical Group, Melbourne, FL:* J Salazar, R Hovland, J Jordan, S Karas, T Peacock, N Schechtmann, G Tischner, R Vicari, K Warren; *Heart and Health Institute Westside, Plantation, FL:* A Ghitis, H Cusner, M Klaus Clark; *Heart Center at St. Mark's Hospital, Salt Lake City, UT:* J Zebrack, S Christensen, C Evenson, D Fullerton; *Heart Center Research LLC, Huntsville, AL:* J Hartley, K Broadway, L Eskridge, D Raymond; *HeartCare Midwest, Peoria, IL:* T Kizhakekuttu, S Hillis, R Klundt, D McElroy; *Heritage Valley Medical Group, Inc, Beaver, PA.:* R Begg, J Acon, A Flores, J Hobbs-Williams, E Schidemantle; *HOPE Research Institute, Phoenix, AZ:* M Cooper, E Campbell, B Corcoran, S Hughes, N Miller, S Steingard; *HOPE Research Institute, Chandler, AZ:* D Einhorn, M Berry, S Dawkins Hughes, L Gilbert, E Lasala, A Loeck, N Mills, J Oppenheim; *Hudson Valley Cardiovascular Practice, P.C., Poughkeepsie, NY:* D O'Dea, S Brian, G Gerber, T Landi, J Ling, S Rimmey; *Imperial Health, LLP, Lake Charles, LA:* R Gilmore, C Bruney, E Gabbert, R Hays, L Stawecki, J Trahan, D Winey-Ward; *Indian River Medical Center – Cardiology, Vero Beach, FL:* S Baker, B Gervasio, J Labodin; *Inova Cardiology Ambulatory Research, Manassas, VA:* H Taheri, J Brooks, A Delozier, J Jayashekaramurthy, S Khachab, P Machineni, K Morgan; *Intermed, PA, Portland, ME:* C Cathcart, E Ciampanelli, W Ervin, K Soule, J Stinson; *Iowa Diabetes and Endocrinology Research Center, Des Moines, IA:* A Bhargava, L Borg, A Carver; *Jacksonville Center for Clinical Research, Jacksonville, FL:* M Koren, A West; *Kootenai Heart Clinics, LLC, Coeur d’Alene, ID:* R Jenkins, S Barnett, H Caro, J Mooney; *Kootenai Heart Clinics, LLC, Spokane, WA:* M Janout, J Bjergo, E Kelley, L Passey, K Sather; *Kore CV Research, Jackson, TN:* E Hage-Korban, M Carrington, A Childs, A Harrington, D Manns, T Phelan; *LeBauer Cardiovascular Research Foundation, Greensboro, NC:* T Stuckey, S Lord, S Milks; *Louisville Metabolic and Atherosclerosis Research Center, Louisville, KY:* H Bays, D Bushong, S Keiran, M Moore, K Weiter; *Lutherville Personal Physicians, Lutherville, MD:* F Morris, C Dignon, J Downing, D Lowry, A Metcalf; *Maine Research Associates, Auburn, ME:* R Weiss, S Dumais; *Marin Endocrine Care & Research, Inc, Greenbrae, CA:* R Bernstein, C Singh; *McLaren Northern Michigan, Petoskey, MI:* A Teklinski, D Antonishen, M Antonishen, M Ronquist, C Shaw; *Medicor Cardiology, Bridgewater, NJ:* J Hall, C Hanzich; Meriter Hospital, Inc., Meriter, Madison, WI: D Lewis, A Gessler, L Skatrud; *Michigan Cardiovascular Institute, Saginaw, MI:* J Collins, V Bitzer, A Fruge, T Gauthier, M Hernandez, K Kayner, C Michon, L Naessens; *MidMichigan Medical Center Midland, Midland, MI:* W Felten, A Cryderman, M Lagalo, K Mostek, C Cluley, J Prior; *MidValley Cardiology, Kingston, NY:* E Lader, M Meyer; *Mobile Heart Specialists, PC, Mobile, AL:* C Alford, S Bryan, M Craig, J Gilley; *MODEL Clinical Research, Baltimore, MD:* P Levin, L Bromberger, D Lowry; *MultiCare Institute for Research & Innovation, Tacoma, WA:* D Guerra, P Brandon, C Burton, J Ebert, K Garrison, C Goetz, S Harris, C Lumsden, D Quinn; *MultiCare Research Institute, Tacoma, WA:* R Graf, K Garrison, S Harris, D Quinn; *National Clinical Research-Richmond, Inc, Richmond, VA:* J Scott, S Ayers, T Beasley, Finney, R Gordon, J Hoekstra, W Jeter, C Young; *Nebraska Heart Institute, Lincoln, NE:* P Dionisopoulos, C Godfrey, R Holcomb, S Krenk; *NJ Heart, Linden, NJ:* P Randhawa, S Agarwal, E Almond, E Capstraw, A Geraldo-Abache, S Kuchipudi, L Pasupuleti, C Sangiovanni, H Sheena, B Vargas; *North Alabama Research Center, LLC, Athens, AL:* E Hendrix, C Crews, J McNeese; *North Ohio Heart Center, Sandusky, OH:* M Traboulssi, A Bohn, K Humphrey, A Walton; *Northwest Heart Clinical Research, LLC, Arlington Heights, IL:* S Lupovitch, S Bellini, L Clemens, M Galindo, V Piszkiewicz, A Soni; *Norton Heart Specialists, Louisville, KY:* J Lash, T Abell, V Flanery, J Hanrahan, D Mudd; *Novant Health Heart and Vascular Institute, Charlotte, NC:* J Pasquini, V Morton, J Nikitin, P Richards, C Sander, M Voelkers; *NYU Hudson Valley Cardiology, Cortlandt Manor, NY:* G Hamroff, L Bentivenga, K Fuerst-Carter, C Hametz, L Hollenweger, C Pankovic, A Solomon; *Ocala Research Institute, INC, Ocala, FL:* R Prashad, T Colacone, M Green, P Lightcap, C McDonough, E Metivier, D Miller; *OhioHealth Research Institute, Mansfield, OH:* M Alton, D Grimwood-Fidler, G Heins, A Looney, L Orr, A Smith; *Oregon Health & Science University, Portland, OR:* W Clark, M Dolan, B Dugan, K Feest, J Foley; *Overlake Medical Clinics Cardiology, Bellevue, WA:* N Perlmutter, R Aviles, W Doucette, T Fortney, J Garceau, J Heywood, K Kanegae, C Kozlowski, D LeDoux, J Leggett, A Mahan, E McKinney, S Ostergard, J Smith, S Wagoner, S Yedinak, N Zilz; *Overlea Personal Physicians, Baltimore, MD:* B Kahn, A Campbell, V Coombs, J Phelps, E Sheridan, M Steinberg; *Palmetto Research Center, LLC, Spartanburg, SC:* R Littlefield, J Baty, A Clark, J Cooper, E Hames; *Parkview Research Center, Fort Wayne, IN:* W Collis, C Moeller, J Needham; *Pentucket Medical Associates, Haverhill, MA:* S Srivastava, S Bilazarian, C Ketis, K Roach; *Permian Research Foundation, Odessa, TX:* F Boccalandro, A Bryan; *Pottstown Medical Specialists, Inc, Pottstown, PA:* J Krantzler, N McClelland, T Muhlenberg, S Pickett; *Premier Healthcare, LLC, Bloomington, IN:* L Rink, E Anderson, A Brooks-Wolfe, B Litz, D Mobley; *Prevea Clinic, Inc., Green Bay, WI:* T Knutson, B Belanger, P Hermans, C Quinnell; *Primary Care Cardiology Research, Inc, Ayer, MA:* T Hack, E Fisher, L Morelli, S Sullivan; *PriMed Physicians, A Member of Northeast Medical Group, Yale New Haven Health, Trumbull, CT:* C Landau, D Ferguson, T Hilts; *Providence Saint Joseph Medical Center, Burbank, CA:* D Eisenberg, G Babar, D Fernando, D Gallegos, G Kenegos, K Reed; *Regions Hospital-Heart Center, St. Paul, MN:* M Danish Rizvi, G Erie, C Eubanks, B Foster, J Kline, W Nelson; *Research Physicians Network Alliance, Hollywood, FL:* L Tami, M Abdur Rahman, J Viera Moreno; *Research Physicians Network Alliance, Pembroke Pines, FL:* P Krichmar, J Ferreira, D Marquez, H Sanchez-Lacayo, R Yunes; *Santa Rosa Cardiology Medical Group, Inc, Santa Rosa, CA:* J Hunter, E Battistelli, T Cook, R Iverson, M Suarez; *Saratoga Cardiology Assoc., PC/Saratoga Clinical Research, LLC, Saratoga Springs, NY:* D Kandath, S Frank, G Kostedt, J Nelson; *South Florida Research Group LLC, Miami, FL:* C Hamburg, L Diaz, E Hernandez, J Roberts, K Shatsky, E Torres; *South Oklahoma Heart Research, LLC, Oklahoma City, OK:* N Tahirkheli, T Adams, K Springer, W Springer; *Southwest Florida Research, LLC, Naples, FL:* J Talano, R Ficarra, L Leo, J Nolen, M Perez, G Rappley, N Szalanski; *Southwest Heart, Tucson, AZ:* B Peart, M Ford-Tarlton, K Peart, J Stephens; *St. Johns Center for Clinical Research, Ponte Vedra, FL:* D Schlager, E Schramm, M Rabalais, C Williamson; *St. Peter's Health Partners Medical Associates - Albany Associates in Cardiology, Albany, NY:* J DeSantis, K Benedetto, E Bursey, T Harting, R Muller, R Phang, E Roccario, P Schaummann-Boyle, A Zuchelkowski; *Tallahassee Research Institute Inc, Tallahassee, FL:* J Katopodis, K Gearld, P Knap, S Liebrich; *TCR Institute, LLC, Norwalk, CT:* I Lieber, L Ferree, F Stowe, M Sutton, D Wiseman; *Tenet Florida Physician Services, Jupiter, FL:* C Vogel, R Aggarwal, C Baroni, P Beck, J Blake, E Dagher, A Gryl, M Johnson, M Smith; *The Carl & Edyth Lindner Center for Research & Education at The Christ Hospital, Cincinnati, OH:* D Kereiakes, C DeFosse, J Schwartz; *The Center for Pharmaceutical Research, P.C., Kansas City, MO:* J Ervin, S Edwards, C Gorman, A Gorsuch, S Pomeroy; *The Polyclinic, Seattle, WA:* K Huehnergarth, K Davis, M Harder, M Lim, M Schrenker, S Yedinak; *The University of Iowa, College of Public Health, Preventive Intervention Center, Iowa City, IA:* J Robinson, J Cayler, M Cherrico, D Chun-Furlong, J De La Garza; *Trinity Medical Center, Rock Island, IL:* A Pothula, C Antonio-Drabek, A Bradley, R Buresh, T Hass, C Lopez*; UCH-MHS, Colorado Springs, CO:* J Strader, Jr., A Donlin, E Ensminger, H Garcia, A Gneiting, E Graf, D Greenberg; *University of Alabama Medical Center, Birmingham, AL:* W Rogers, P Arora, T Morgan, L Saag, S Thorington; *University of Maryland, Westminster, MD:* S Jerome, L Black, A Gupta; *University of Missouri Health System, Columbia, MO:* K Aggarwal, K Belew, V Burkhardt, S Collins, S Holland Clasby, A Lau-Sieckman; *Upstate Cardiology, Greenville, SC:* J Cebe, E Calhoun, C Kissam, L Major; *Verde Valley Medical Center, Cottonwood, AZ:* S Butman, K Bescak, D Bescak, A Bigelow, T Brown, S Davidson; *Virginia Heart, Falls Church, VA:* T Haddad, T Alexander, J Jain, T Myhera, D Overbeck, B Torre, L Wotorson; *Watson Clinic LLP, Lakeland, FL:* J Canto, C Corneal, B Donley, N McGowan, K Prisoc, M Sharrett; *Wenatchee Valley Hospital & Clinics, Wenatchee, WA:* S Kaster, J Akers, H Darlington, J Gault, J Horner, C Roozen; *Westlake Medical Research, Thousand Oaks, CA:* I Loh, R Anderson, T Call, J Esaki, P Patel, J Plocky, J Raymond, C Rideaux, L Sprafka; *Westside Center for Clinical Research, Jacksonville, FL:* M Stich, T Alexander, C Andres, C Brown, M Buda, S Ciuica, B Minker, S Perry; *York Hospital, York, PA:* K McCullum, B Doty, S Gates, K Hutcheson.

# Supplementary References

1. HPS2-THRIVE Collaborative Group. Effects of extended-release niacin with laropiprant in high-risk patients. *N Engl J Med* 2014; **371**(3): 203-12.

2. Marcovina SM, Albers JJ. Lipoprotein (a) measurements for clinical application. *J Lipid Res* 2016; **57**(4): 526-37.

3. Levey AS, Stevens LA, Schmid CH, et al. A new equation to estimate glomerular filtration rate. *Ann Intern Med* 2009; **150**(9): 604-12.

4. Thygesen K, Alpert JS, Jaffe AS, et al. Third universal definition of myocardial infarction. *Circulation* 2012; **126**(16): 2020-35.

5. Cholesterol Treatment Trialists' (CTT) Collaboration. Efficacy and safety of cholesterol-lowering treatment: prospective meta-analysis of data from 90 056 participants in 14 randomised trials of statins. *Lancet* 2005; **366**(9493): 1267-78.

6. Cholesterol Treatment Trialists' (CTT) Collaboration. Efficacy and safety of more intensive lowering of LDL cholesterol: a meta-analysis of data from 170 000 participants in 26 randomised trials. *Lancet* 2010; **376**(9753): 1670-81.

7. Di Angelantonio E, Sarwar N, Perry P, et al. Major lipids, apolipoproteins, and risk of vascular disease. *JAMA* 2009; **302**(18): 1993-2000.

8. Pocock SJ, Simon R. Sequential treatment assignment with balancing for prognostic factors in the controlled clinical trial. *Biometrics* 1975; **31**(1): 103-15.

9. Bohula EA, Bonaca MP, Braunwald E, et al. Atherothrombotic Risk Stratification and the Efficacy and Safety of Vorapaxar in Patients With Stable Ischemic Heart Disease and Previous Myocardial Infarction. *Circulation* 2016; **134**(4): 304-13.

# Supplementary Tables and Figures

## Supplementary Table I: Recruitment by country and region

|  | **Centres*** | **Screened** | **Entered**  **run-in** | **Attended**  **Randomization** | **Randomized** |
| --- | --- | --- | --- | --- | --- |
| **Europe** | **191** | **29318** | **20666** | **17736** | **15738** |
| United Kingdom | 64 | 19125 | 11772 | 9784 | 8381 |
| Finland | 11 | 785 | 701 | 660 | 613 |
| Sweden | 18 | 1194 | 1014 | 931 | 861 |
| Norway | 17 | 1201 | 1022 | 905 | 844 |
| Denmark | 19 | 2290 | 2091 | 1946 | 1850 |
| Italy | 26 | 2273 | 2014 | 1770 | 1660 |
| Germany | 36 | 2450 | 2052 | 1740 | 1529 |
|  |  |  |  |  |  |
| **North America** | **174** | **8249** | **7318** | **6474** | **6082** |
| United States | 157 | 7270 | 6450 | 5694 | 5330 |
| Canada | 17 | 979 | 868 | 780 | 752 |
|  |  |  |  |  |  |
| **China** | **78** | **12220** | **10262** | **9216** | **8629** |
|  |  |  |  |  |  |
| **Total** | **443** | **49787** | **38246** | **33426** | **30449** |

* Count of centres that screened at least one person.

## Supplementary Table II: Reasons screened patients did not enter run-in

|  | **Europe** | | **North America** | | **China** | | **Total** | |
| --- | --- | --- | --- | --- | --- | --- | --- | --- |
| **Attended screening** | **29318** |  | **8249** |  | **12220** |  | **49787** |  |
|  |  |  |  |  |  |  |  |  |
| **Inclusion criteria not met** | 1321 | (4.5%) | 74 | (0.9%) | 11 | (0.1%) | 1406 | (2.8%) |
|  |  |  |  |  |  |  |  |  |
| **Fulfilled one or more exclusion criteria** |  |  |  |  |  |  |  |  |
| **Medical History** |  |  |  |  |  |  |  |  |
| Recent myocardial infarction | 8 | (0.0%) | 2 | (0.0%) | 0 | (0.0%) | 10 | (0.0%) |
| Recent acute coronary syndrome | 34 | (0.1%) | 3 | (0.0%) | 4 | (0.0%) | 41 | (0.1%) |
| Recent stroke | 2 | (0.0%) | 0 | (0.0%) | 3 | (0.0%) | 5 | (0.0%) |
| On waiting-list for coronary revascularization | 79 | (0.3%) | 2 | (0.0%) | 3 | (0.0%) | 84 | (0.2%) |
| Chronic liver disease | 83 | (0.3%) | 5 | (0.1%) | 23 | (0.2%) | 111 | (0.2%) |
| Severe renal disease | 143 | (0.5%) | 3 | (0.0%) | 6 | (0.0%) | 152 | (0.3%) |
| Inflammatory muscle disease | 117 | (0.4%) | 4 | (0.0%) | 0 | (0.0%) | 121 | (0.2%) |
| Severe respiratory disease | 554 | (1.9%) | 8 | (0.1%) | 6 | (0.0%) | 568 | (1.1%) |
| Cancer | 231 | (0.8%) | 54 | (0.7%) | 23 | (0.2%) | 308 | (0.6%) |
| Alcohol or substance misuse | 156 | (0.5%) | 3 | (0.0%) | 0 | (0.0%) | 159 | (0.3%) |
| Child-bearing potential | 4 | (0.0%) | 1 | (0.0%) | 0 | (0.0%) | 5 | (0.0%) |
| Any of the above | 1295 | (4.4%) | 81 | (1.0%) | 65 | (0.5%) | 1441 | (2.9%) |
| **Medication History** |  |  |  |  |  |  |  |  |
| Previous adverse reaction to a statin | 715 | (2.4%) | 12 | (0.1%) | 8 | (0.1%) | 735 | (1.5%) |
| Previous adverse reaction to a CETP inhibitor | 2 | (0.0%) | 2 | (0.0%) | 0 | (0.0%) | 4 | (0.0%) |
| Contra-indicated medication | 95 | (0.3%) | 25 | (0.3%) | 15 | (0.1%) | 135 | (0.3%) |
| In a clinical trial with an unlicensed medication or device | 65 | (0.2%) | 5 | (0.1%) | 3 | (0.0%) | 73 | (0.1%) |
| Already on potent LDL-lowering treatment* | 505 | (1.7%) | 84 | (1.0%) | 32 | (0.3%) | 621 | (1.2%) |
| Any of the above | 1366 | (4.7%) | 126 | (1.5%) | 58 | (0.5%) | 1550 | (3.1%) |
|  |  |  |  |  |  |  |  |  |
| **Concerns about long-term compliance and/or attendance** | 1312 | (4.5%) | 13 | (0.2%) | 67 | (0.5%) | 1392 | (2.8%) |
|  |  |  |  |  |  |  |  |  |
| **Consent not given** | 919 | (3.1%) | 8 | (0.1%) | 36 | (0.3%) | 963 | (1.9%) |
|  |  |  |  |  |  |  |  |  |
| **Blood results** |  |  |  |  |  |  |  |  |
| Blood results incomplete | 210 | (0.7%) | 97 | (1.2%) | 81 | (0.7%) | 388 | (0.8%) |
| Creatine kinase greater than 3xULN | 99 | (0.3%) | 17 | (0.2%) | 16 | (0.1%) | 132 | (0.3%) |
| Alanine transaminase greater than 2xULN | 111 | (0.4%) | 48 | (0.6%) | 40 | (0.3%) | 199 | (0.4%) |
| Creatinine >2.3 mg/dL | 69 | (0.2%) | 35 | (0.4%) | 38 | (0.3%) | 142 | (0.3%) |
| Predicted total cholesterol on study statin ≥135 mg/dL | 2649 | (9.0%) | 533 | (6.5%) | 1632 | (13.4%) | 4814 | (9.7%) |
| Any of the above | 3070 | (10.5%) | 641 | (7.8%) | 1726 | (14.1%) | 5437 | (10.9%) |
|  |  |  |  |  |  |  |  |  |
| **Attended screening but did not enter run-in^†^** | 8652 | (29.5%) | 931 | (11.3%) | 1958 | (16.0%) | 11541 | (23.2%) |
|  |  |  |  |  |  |  |  |  |
| **Entered run-in** | **20666** | **(70.5%)** | **7318** | **(88.7%)** | **10262** | **(84.0%)** | **38246** | **(76.8%)** |
| CETP – cholesteryl ester transfer protein; ULN – upper limit of normal.  * The LDL-lowering treatment that the participant was taking at screening was more potent than the highest study atorvastatin dose available in that region.  **^†^** Participants may have more than one reason for not entering run-in. | | | | | | | | |

## Supplementary Table III: Reasons for withdrawing from the run-in period

|  | **Europe** | | **North America** | | **China** | | **Total** | |
| --- | --- | --- | --- | --- | --- | --- | --- | --- |
| **Entered run-in** | **20666** |  | **7318** |  | **10262** |  | **38246** |  |
|  |  |  |  |  |  |  |  |  |
| **Death** |  |  |  |  |  |  |  |  |
| Cardiovascular death | 15 | (0.1%) | 4 | (0.1%) | 13 | (0.1%) | 32 | (0.1%) |
| Non-cardiovascular death | 7 | (0.0%) | 7 | (0.1%) | 6 | (0.1%) | 20 | (0.1%) |
| Any death | 22 | (0.1%) | 11 | (0.2%) | 19 | (0.2%) | 52 | (0.1%) |
|  |  |  |  |  |  |  |  |  |
| **Serious adverse event*** |  |  |  |  |  |  |  |  |
| Cardiac disorders | 47 | (0.2%) | 23 | (0.3%) | 20 | (0.2%) | 90 | (0.2%) |
| Neoplasms | 15 | (0.1%) | 9 | (0.1%) | 13 | (0.1%) | 37 | (0.1%) |
| Nervous system disorders | 26 | (0.1%) | 11 | (0.2%) | 7 | (0.1%) | 44 | (0.1%) |
| Surgical and medical procedures | 11 | (0.1%) | 9 | (0.1%) | 4 | (0.0%) | 24 | (0.1%) |
| Other serious adverse event* | 44 | (0.2%) | 22 | (0.3%) | 9 | (0.1%) | 75 | (0.2%) |
| Any serious adverse event* | 140 | (0.7%) | 72 | (1.0%) | 53 | (0.5%) | 265 | (0.7%) |
|  |  |  |  |  |  |  |  |  |
| **Non-serious adverse event** |  |  |  |  |  |  |  |  |
| Gastrointestinal disorders | 366 | (1.8%) | 51 | (0.7%) | 40 | (0.4%) | 457 | (1.2%) |
| General disorders and administration site conditions | 44 | (0.2%) | 9 | (0.1%) | 9 | (0.1%) | 62 | (0.2%) |
| Infections and infestations | 20 | (0.1%) | 5 | (0.1%) | 3 | (0.0%) | 28 | (0.1%) |
| Investigations | 20 | (0.1%) | 7 | (0.1%) | 1 | (0.0%) | 28 | (0.1%) |
| Musculoskeletal and connective tissue disorders | 481 | (2.3%) | 172 | (2.4%) | 24 | (0.2%) | 677 | (1.8%) |
| Nervous system disorders | 134 | (0.6%) | 17 | (0.2%) | 12 | (0.1%) | 163 | (0.4%) |
| Psychiatric disorders | 36 | (0.2%) | 10 | (0.1%) | 1 | (0.0%) | 47 | (0.1%) |
| Respiratory, thoracic and mediastinal disorders | 27 | (0.1%) | 4 | (0.1%) | 2 | (0.0%) | 33 | (0.1%) |
| Skin and subcutaneous tissue disorders | 64 | (0.3%) | 8 | (0.1%) | 10 | (0.1%) | 82 | (0.2%) |
| Other non-serious adverse event | 59 | (0.3%) | 27 | (0.4%) | 18 | (0.2%) | 104 | (0.3%) |
| Any non-serious adverse event | 1208 | (5.8%) | 293 | (4.0%) | 120 | (1.2%) | 1621 | (4.2%) |
|  |  |  |  |  |  |  |  |  |
| **Other reason** |  |  |  |  |  |  |  |  |
| Concerns about tablets | 633 | (3.1%) | 143 | (2.0%) | 577 | (5.6%) | 1353 | (3.5%) |
| Contraindicated drug started | 15 | (0.1%) | 4 | (0.1%) | 1 | (0.0%) | 20 | (0.1%) |
| Difficulty taking tablets | 97 | (0.5%) | 37 | (0.5%) | 23 | (0.2%) | 157 | (0.4%) |
| Doctor advice | 223 | (1.1%) | 31 | (0.4%) | 22 | (0.2%) | 276 | (0.7%) |
| Family circumstances | 34 | (0.2%) | 16 | (0.2%) | 29 | (0.3%) | 79 | (0.2%) |
| Travel problem | 111 | (0.5%) | 14 | (0.2%) | 10 | (0.1%) | 135 | (0.4%) |
| Trial administration problem | 126 | (0.6%) | 108 | (1.5%) | 32 | (0.3%) | 266 | (0.7%) |
| Unable to attend clinic | 221 | (1.1%) | 106 | (1.4%) | 154 | (1.5%) | 481 | (1.3%) |
| Undergoing investigations | 64 | (0.3%) | 3 | (0.0%) | 3 | (0.0%) | 70 | (0.2%) |
| Other reason not listed above | 46 | (0.2%) | 6 | (0.1%) | 5 | (0.0%) | 57 | (0.1%) |
| Any other reason for stopping treatment | 1565 | (7.6%) | 468 | (6.4%) | 854 | (8.3%) | 2887 | (7.5%) |
|  |  |  |  |  |  |  |  |  |
| **Dropped out of run-in^†^** | 2930 | (14.2%) | 844 | (11.5%) | 1046 | (10.2%) | 4820 | (12.6%) |
|  |  |  |  |  |  |  |  |  |
| **Attended randomization visit** | **17736** | **(85.8%)** | **6474** | **(88.5%)** | **9216** | **(89.8%)** | **33426** | **(87.4%)** |
| * Includes 4 suspected serious adverse reactions to run-in medication (atorvastatin 80 mg daily plus placebo): 1 case of myopathy with muscle symptoms, creatine kinase (CK) >10x upper limit of normal (ULN) but no evidence of end organ damage; 1 case of increased alanine transaminase >4x ULN with alkaline phosphatase >2x ULN, but normal bilirubin, liver and biliary tract imaging; 1 case of life-threatening erythema multiforme starting 7 days after changing from previous simvastatin to study run-in medication); and 1 case of abdominal and chest wall pain with normal electrocardiogram and CK >3 ≤5x upper limit of normal, starting 3 days after changing from previous pravastatin to study run-in medication. All recovered following cessation of study run-in treatment.  **^†^** Participants may have more than one reason for dropping out of run-in. | | | | | | | | |

## Supplementary Table IV: Reasons for exclusion at randomization visit

|  | **Europe** | | **North America** | | **China** | | **Total** | | |
| --- | --- | --- | --- | --- | --- | --- | --- | --- | --- |
| **Attended randomization visit** | **17736** |  | **6474** |  | **9216** |  | **33426** |  | |
|  |  |  |  |  |  |  |  |  | |
| **Inclusion criteria not met** | 5 | (0.0%) | 1 | (0.0%) | 0 | (0.0%) | 6 | (0.0%) | |
|  |  |  |  |  |  |  |  |  | |
| **Fulfilled one or more exclusion criteria** |  |  |  |  |  |  |  |  | |
| Medical History |  |  |  |  |  |  |  |  | |
| Recent myocardial infarction | 13 | (0.1%) | 6 | (0.1%) | 0 | (0.0%) | 19 | (0.1%) | |
| Recent acute coronary syndrome | 48 | (0.3%) | 13 | (0.2%) | 19 | (0.2%) | 80 | (0.2%) | |
| Recent stroke | 8 | (0.0%) | 2 | (0.0%) | 16 | (0.2%) | 26 | (0.1%) | |
| Recent percutaneous coronary intervention | 2 | (0.0%) | 0 | (0.0%) | 0 | (0.0%) | 2 | (0.0%) | |
| On waiting-list for coronary revascularization | 20 | (0.1%) | 3 | (0.0%) | 3 | (0.0%) | 26 | (0.1%) | |
| Cancer | 39 | (0.2%) | 17 | (0.3%) | 3 | (0.0%) | 59 | (0.2%) | |
| Muscle symptoms | 541 | (3.1%) | 91 | (1.4%) | 50 | (0.5%) | 682 | (2.0%) | |
| Symptoms suggestive of hepatitis | 254 | (1.4%) | 31 | (0.5%) | 16 | (0.2%) | 301 | (0.9%) | |
| Suspected serious adverse reaction* | 5 | (0.0%) | 1 | (0.0%) | 0 | (0.0%) | 6 | (0.0%) | |
| Any of the above | 816 | (4.6%) | 143 | (2.2%) | 104 | (1.1%) | 1063 | (3.2%) | |
|  |  |  |  |  |  |  |  |  | |
| **Medication History** |  |  |  |  |  |  |  |  | |
| Contra-indicated medication | 86 | (0.5%) | 8 | (0.1%) | 15 | (0.2%) | 109 | (0.3%) | |
| Poor compliance with run-in treatment | 204 | (1.2%) | 59 | (0.9%) | 134 | (1.5%) | 397 | (1.2%) | |
| Any of the above | 250 | (1.4%) | 61 | (0.9%) | 137 | (1.5%) | 448 | (1.3%) | |
|  |  |  |  |  |  |  |  |  | |
| **Concern about long-term compliance** | 441 | (2.5%) | 52 | (0.8%) | 87 | (0.9%) | 580 | (1.7%) | |
| Serious adverse event |  |  |  |  |  |  |  |  | |
| Any serious adverse event | 17 | (0.1%) | 0 | (0.0%) | 2 | (0.0%) | 19 | (0.1%) | |
| Non-serious adverse event |  |  |  |  |  |  |  |  | |
| Gastrointestinal disorders | 44 | (0.2%) | 2 | (0.0%) | 3 | (0.0%) | 49 | (0.1%) | |
| Musculoskeletal and connective tissue disorders | 73 | (0.4%) | 19 | (0.3%) | 5 | (0.1%) | 97 | (0.3%) | |
| Nervous system disorders | 24 | (0.1%) | 2 | (0.0%) | 1 | (0.0%) | 27 | (0.1%) | |
| Other | 31 | (0.2%) | 4 | (0.1%) | 3 | (0.0%) | 38 | (0.1%) | |
| Any non-serious adverse event | 171 | (1.0%) | 27 | (0.4%) | 12 | (0.1%) | 210 | (0.6%) | |
| Other reason |  |  |  |  |  |  |  |  | |
| Concerns about tablets | 81 | (0.5%) | 12 | (0.2%) | 46 | (0.5%) | 139 | (0.4%) | |
| Difficulty taking tablets | 41 | (0.2%) | 4 | (0.1%) | 7 | (0.1%) | 52 | (0.2%) | |
| Unable to attend clinic | 27 | (0.2%) | 3 | (0.0%) | 6 | (0.1%) | 36 | (0.1%) | |
| Undergoing investigations | 23 | (0.1%) | 0 | (0.0%) | 2 | (0.0%) | 25 | (0.1%) | |
| Other reason not listed above | 81 | (0.5%) | 6 | (0.1%) | 12 | (0.1%) | 99 | (0.3%) | |
| Any other reason | 253 | (1.4%) | 25 | (0.4%) | 73 | (0.8%) | 351 | (1.1%) | |
|  |  |  |  |  |  |  |  |  | |
| **Unwilling or unable to continue** | 617 | (3.5%) | 89 | (1.4%) | 101 | (1.1%) | 807 | (2.4%) | |
|  |  |  |  |  |  |  |  |  | |
| **Blood results** |  |  |  |  |  |  |  |  | |
| Blood results incomplete | 168 | (0.9%) | 47 | (0.7%) | 20 | (0.2%) | 235 | (0.7%) | |
| Creatine kinase greater than 3xULN | 60 | (0.3%) | 18 | (0.3%) | 12 | (0.1%) | 90 | (0.3%) | |
| Alanine transaminase greater than 2xULN | 142 | (0.8%) | 37 | (0.6%) | 42 | (0.5%) | 221 | (0.7%) | |
| Total cholesterol >155 mg/dL | 635 | (3.6%) | 140 | (2.2%) | 322 | (3.5%) | 1097 | (3.3%) | |
| Any of the above | 976 | (5.5%) | 239 | (3.7%) | 391 | (4.2%) | 1606 | (4.8%) | |
|  |  |  |  |  |  |  |  |  | |
| Attended randomization visit but was not randomized^†^ | 1998 | (11.3%) | 392 | (6.1%) | 587 | (6.4%) | 2977 | (8.9%) | |
|  |  |  |  |  |  |  |  |  | |
| **Randomized** | **15738** | **(88.7%)** | **6082** | **(93.9%)** | **8629** | **(93.6%)** | **30449** | **(91.1%)** | |
| ULN – upper limit of normal  ***** Of these 6 cases, 5 were subsequently determined not to meet the criteria for a serious adverse reaction to study medication. There was 1 participant who received atorvastatin 80 mg daily plus placebo during the run-in period and who developed myopathy with muscle symptoms and creatine kinase >10x upper limit of normal (but no evidence of end organ damage). The participant recovered following cessation of study run-in treatment.  **^†^** Participants may have more than one reason for not being randomized. | | | | | | | | |  |

## Supplementary Table V: Baseline characteristics of randomized participants

|  | **Europe** | | **North America** | | **China** | | **Total** | |
| --- | --- | --- | --- | --- | --- | --- | --- | --- |
| Number randomized | 15738 |  | 6082 |  | 8629 |  | 30449 |  |
|  |  |  |  |  |  |  |  |  |
| **Atorvastatin dose*** |  |  |  |  |  |  |  |  |
| Low | 7969 | (50.6%) | 2621 | (43.1%) | 4519 | (52.4%) | 15109 | (49.6%) |
| High | 7769 | (49.4%) | 3461 | (56.9%) | 4110 | (47.6%) | 15340 | (50.4%) |
|  |  |  |  |  |  |  |  |  |
| **Prior disease**^†^ |  |  |  |  |  |  |  |  |
| Coronary heart disease | 13306 | (84.5%) | 5697 | (93.7%) | 7676 | (89.0%) | 26679 | (87.6%) |
| Cerebrovascular disease | 3348 | (21.3%) | 1131 | (18.6%) | 2302 | (26.7%) | 6781 | (22.3%) |
| Peripheral arterial disease | 1645 | (10.5%) | 714 | (11.7%) | 76 | (0.9%) | 2435 | (8.0%) |
| Diabetes | 4678 | (29.7%) | 2682 | (44.1%) | 3960 | (45.9%) | 11320 | (37.2%) |
| Heart failure | 785 | (5.0%) | 343 | (5.6%) | 643 | (7.5%) | 1771 | (5.8%) |
|  |  |  |  |  |  |  |  |  |
| **Time since qualifying event** |  |  |  |  |  |  |  |  |
| < 12 Months | 2420 | (15.4%) | 1060 | (17.4%) | 1933 | (22.4%) | 5413 | (17.8%) |
| ≥ 12 Months | 13210 | (83.9%) | 4995 | (82.1%) | 6655 | (77.1%) | 24860 | (81.6%) |
| Unknown | 108 | (0.7%) | 27 | (0.4%) | 41 | (0.5%) | 176 | (0.6%) |
|  |  |  |  |  |  |  |  |  |
| **Age (years)** | 68 | ±8 | 67 | ±8 | 64 | ±8 | 67 | ±8 |
| < 65 | 5852 | (37.2%) | 2399 | (39.4%) | 5026 | (58.2%) | 13277 | (43.6%) |
| ≥ 65 < 70 | 3859 | (24.5%) | 1407 | (23.1%) | 1491 | (17.3%) | 6757 | (22.2%) |
| ≥ 70 | 6027 | (38.3%) | 2276 | (37.4%) | 2112 | (24.5%) | 10415 | (34.2%) |
|  |  |  |  |  |  |  |  |  |
| **Gender** |  |  |  |  |  |  |  |  |
| Male | 13652 | (86.7%) | 5058 | (83.2%) | 6824 | (79.1%) | 25534 | (83.9%) |
| Female | 2086 | (13.3%) | 1024 | (16.8%) | 1805 | (20.9%) | 4915 | (16.1%) |
|  |  |  |  |  |  |  |  |  |
| **Systolic blood pressure (mmHg)** | 131 | ±17.9 | 129 | ±17.7 | 133 | ±19.9 | 131 | ±18.5 |
| < 125 | 5919 | (37.6%) | 2563 | (42.1%) | 2956 | (34.3%) | 11438 | (37.6%) |
| ≥ 125 < 140 | 5041 | (32.0%) | 1913 | (31.5%) | 2605 | (30.2%) | 9559 | (31.4%) |
| ≥ 140 | 4778 | (30.4%) | 1606 | (26.4%) | 3068 | (35.6%) | 9452 | (31.0%) |
|  |  |  |  |  |  |  |  |  |
| **Diastolic blood pressure (mmHg)** | 78.1 | ±10.7 | 76.3 | ±10.8 | 79.2 | ±11.6 | 78.1 | ±11.0 |
| < 75 | 5741 | (36.5%) | 2658 | (43.7%) | 3047 | (35.3%) | 11446 | (37.6%) |
| ≥ 75 < 85 | 5657 | (35.9%) | 2089 | (34.3%) | 2939 | (34.1%) | 10685 | (35.1%) |
| ≥ 85 | 4340 | (27.6%) | 1335 | (22.0%) | 2643 | (30.6%) | 8318 | (27.3%) |
|  |  |  |  |  |  |  |  |  |
| **Body mass index (kg/m²)** | 29.0 | ±4.8 | 31.0 | ±6.0 | 26.1 | ±3.4 | 28.6 | ±5.1 |
| < 25 | 2826 | (18.0%) | 716 | (11.8%) | 3266 | (37.8%) | 6808 | (22.4%) |
| ≥ 25 < 30 | 7202 | (45.8%) | 2251 | (37.0%) | 4447 | (51.5%) | 13900 | (45.7%) |
| ≥ 30 | 5673 | (36.0%) | 3112 | (51.2%) | 912 | (10.6%) | 9697 | (31.8%) |
| Missing | 37 | (0.2%) | 3 | (0.0%) | 4 | (0.0%) | 44 | (0.1%) |
|  |  |  |  |  |  |  |  |  |
| **Waist:hip ratio^‡^** | 0.97 | ±0.08 | 0.98 | ±0.08 | 0.92 | ±0.05 | 0.96 | ±0.07 |
| Low | 3927 | (25.0%) | 1334 | (21.9%) | 4827 | (55.9%) | 10088 | (33.1%) |
| Medium | 5319 | (33.8%) | 2086 | (34.3%) | 2733 | (31.7%) | 10138 | (33.3%) |
| High | 6462 | (41.1%) | 2655 | (43.7%) | 1045 | (12.1%) | 10162 | (33.4%) |
| Missing | 30 | (0.2%) | 7 | (0.1%) | 24 | (0.3%) | 61 | (0.2%) |
|  |  |  |  |  |  |  |  |  |
| **Alcohol intake** |  |  |  |  |  |  |  |  |
| Current drinker | 7723 | (49.1%) | 1365 | (22.4%) | 969 | (11.2%) | 10057 | (33.0%) |
| Former/Non drinker | 8015 | (50.9%) | 4717 | (77.6%) | 7660 | (88.8%) | 20392 | (67.0%) |
|  |  |  |  |  |  |  |  |  |
| **Cigarette smoking** |  |  |  |  |  |  |  |  |
| Current smoker | 2004 | (12.7%) | 770 | (12.7%) | 2029 | (23.5%) | 4803 | (15.8%) |
| Former smoker | 7712 | (49.0%) | 2301 | (37.8%) | 2582 | (29.9%) | 12595 | (41.4%) |
| Non-smoker | 6022 | (38.3%) | 3011 | (49.5%) | 4018 | (46.6%) | 13051 | (42.9%) |
|  |  |  |  |  |  |  |  |  |
| **Medication** |  |  |  |  |  |  |  |  |
| ACE inhibitor or ARB | 11879 | (75.5%) | 4400 | (72.3%) | 4050 | (46.9%) | 20329 | (66.8%) |
| Antiplatelet therapy | 14261 | (90.6%) | 5654 | (93.0%) | 7992 | (92.6%) | 27907 | (91.7%) |
| Diuretic | 3965 | (25.2%) | 1831 | (30.1%) | 728 | (8.4%) | 6524 | (21.4%) |
| Calcium channel blocker | 4045 | (25.7%) | 1461 | (24.0%) | 2679 | (31.0%) | 8185 | (26.9%) |
| Beta-blocker | 10897 | (69.2%) | 4665 | (76.7%) | 5112 | (59.2%) | 20674 | (67.9%) |
|  |  |  |  |  |  |  |  |  |
| **Total cholesterol (mg/dL)** | 136 | ±20.7 | 126 | ±21.0 | 129 | ±21.8 | 132 | ±21.4 |
| <124 | 4381 | (27.8%) | 2654 | (43.6%) | 3599 | (41.7%) | 10634 | (34.9%) |
| ≥124 <143 | 5575 | (35.4%) | 1977 | (32.5%) | 2867 | (33.2%) | 10419 | (34.2%) |
| ≥143 | 5706 | (36.3%) | 1267 | (20.8%) | 2153 | (25.0%) | 9126 | (30.0%) |
| Missing | 76 | (0.5%) | 184 | (3.0%) | 10 | (0.1%) | 270 | (0.9%) |
|  |  |  |  |  |  |  |  |  |
| **LDL cholesterol (mg/dL)** | 63.3 | ±15.3 | 57.6 | ±14.3 | 59.0 | ±15.0 | 61.0 | ±15.2 |
| <54 | 4306 | (27.4%) | 2471 | (40.6%) | 3298 | (38.2%) | 10075 | (33.1%) |
| ≥54 <66 | 4653 | (29.6%) | 1811 | (29.8%) | 2642 | (30.6%) | 9106 | (29.9%) |
| ≥66 | 6703 | (42.6%) | 1616 | (26.6%) | 2679 | (31.0%) | 10998 | (36.1%) |
| Missing | 76 | (0.5%) | 184 | (3.0%) | 10 | (0.1%) | 270 | (0.9%) |
|  |  |  |  |  |  |  |  |  |
| **HDL cholesterol (mg/dL)** | 42.8 | ±10.1 | 37.4 | ±8.91 | 37.5 | ±7.71 | 40.2 | ±9.61 |
| <35 | 3269 | (20.8%) | 2516 | (41.4%) | 3383 | (39.2%) | 9168 | (30.1%) |
| ≥35 <43 | 5240 | (33.3%) | 1958 | (32.2%) | 3264 | (37.8%) | 10462 | (34.4%) |
| ≥43 | 7153 | (45.5%) | 1424 | (23.4%) | 1972 | (22.9%) | 10549 | (34.6%) |
| Missing | 76 | (0.5%) | 184 | (3.0%) | 10 | (0.1%) | 270 | (0.9%) |
|  |  |  |  |  |  |  |  |  |
| **Non-HDL cholesterol (mg/dL)** | 92.8 | ±18.7 | 89.1 | ±18.8 | 91.5 | ±19.8 | 91.7 | ±19.1 |
| <85 | 5451 | (34.6%) | 2535 | (41.7%) | 3344 | (38.8%) | 11330 | (37.2%) |
| ≥85 <101 | 5046 | (32.1%) | 1821 | (29.9%) | 2634 | (30.5%) | 9501 | (31.2%) |
| ≥101 | 5165 | (32.8%) | 1542 | (25.4%) | 2641 | (30.6%) | 9348 | (30.7%) |
| Missing | 76 | (0.5%) | 184 | (3.0%) | 10 | (0.1%) | 270 | (0.9%) |
|  |  |  |  |  |  |  |  |  |
| **Triglycerides (mg/dL)** | 119 | (87-167) | 122 | (87-172) | 135 | (97-192) | 124 | (90-175) |
| <106 | 6254 | (39.7%) | 2321 | (38.2%) | 2728 | (31.6%) | 11303 | (37.1%) |
| ≥106 <151 | 4365 | (27.7%) | 1580 | (26.0%) | 2242 | (26.0%) | 8187 | (26.9%) |
| ≥151 | 5043 | (32.0%) | 1997 | (32.8%) | 3649 | (42.3%) | 10689 | (35.1%) |
| Missing | 76 | (0.5%) | 184 | (3.0%) | 10 | (0.1%) | 270 | (0.9%) |
|  |  |  |  |  |  |  |  |  |
| **Apolipoprotein A1 (mg/dL)** | 124.4 | ±18.5 | 115.1 | ±17.9 | 116.4 | ±14.8 | 120.3 | ±17.9 |
| <110 | 3409 | (21.7%) | 2424 | (39.9%) | 2972 | (34.4%) | 8805 | (28.9%) |
| ≥110 <125 | 5258 | (33.4%) | 2008 | (33.0%) | 3531 | (40.9%) | 10797 | (35.5%) |
| ≥125 | 6995 | (44.4%) | 1467 | (24.1%) | 2116 | (24.5%) | 10578 | (34.7%) |
| Missing | 76 | (0.5%) | 183 | (3.0%) | 10 | (0.1%) | 269 | (0.9%) |
|  |  |  |  |  |  |  |  |  |
| **Apolipoprotein B (mg/dL)** | 67.0 | ±12.7 | 63.6 | ±12.2 | 60.9 | ±12.1 | 64.6 | ±12.7 |
| <60 | 4632 | (29.4%) | 2493 | (41.0%) | 4502 | (52.2%) | 11627 | (38.2%) |
| ≥60 <70 | 4818 | (30.6%) | 1711 | (28.1%) | 2246 | (26.0%) | 8775 | (28.8%) |
| ≥70 | 6212 | (39.5%) | 1693 | (27.8%) | 1861 | (21.6%) | 9766 | (32.1%) |
| Missing | 76 | (0.5%) | 185 | (3.0%) | 20 | (0.2%) | 281 | (0.9%) |
|  |  |  |  |  |  |  |  |  |
| **Lipoprotein(a) (nmol/L)** | 22.4 | (8.2-104) | 26.0 | (8.8-111) | 22.0 | (9.9-61.2) | 22.8 | (8.9-87.6) |
| <15 | 6285 | (39.9%) | 2209 | (36.3%) | 3257 | (37.7%) | 11751 | (38.6%) |
| ≥15 <55 | 4023 | (25.6%) | 1501 | (24.7%) | 3015 | (34.9%) | 8539 | (28.0%) |
| ≥55 | 5326 | (33.8%) | 2187 | (36.0%) | 2347 | (27.2%) | 9860 | (32.4%) |
| Missing | 104 | (0.7%) | 185 | (3.0%) | 10 | (0.1%) | 299 | (1.0%) |
|  |  |  |  |  |  |  |  |  |
| **Glomerular Filtration Rate (ml/min/1.73m²)^§^** | 80.8 | ±16.5 | 80.2 | ±18.0 | 90.1 | ±15.2 | 83.3 | ±17.0 |
| <60 | 2001 | (12.7%) | 883 | (14.5%) | 466 | (5.4%) | 3350 | (11.0%) |
| ≥60 | 13661 | (86.8%) | 5015 | (82.5%) | 8153 | (94.5%) | 26829 | (88.1%) |
| Missing | 76 | (0.5%) | 184 | (3.0%) | 10 | (0.1%) | 270 | (0.9%) |
|  |  |  |  |  |  |  |  |  |
| **Urinary albumin:creatinine ratio (mg/g)^‖^** | 7.6 | (3.7-21.2) | 7.9 | (3.6-28.9) | 8.1 | (3.9-23.1) | 7.8 | (3.8-22.9) |
| Normal | 12520 | (79.6%) | 4706 | (77.4%) | 7187 | (83.3%) | 24413 | (80.2%) |
| Micro-albuminuria | 2005 | (12.7%) | 806 | (13.3%) | 1160 | (13.4%) | 3971 | (13.0%) |
| Macro-albuminuria | 336 | (2.1%) | 201 | (3.3%) | 248 | (2.9%) | 785 | (2.6%) |
| Missing | 877 | (5.6%) | 369 | (6.1%) | 34 | (0.4%) | 1280 | (4.2%) |

Results are count (%), mean ±standard deviation or median (inter-quartile range).

ACE – angiotensin converting enzyme; ARB – angiotensin receptor blocker; LDL – low density lipoprotein; HDL – high density lipoprotein.

* Low dose atorvastatin = 10 mg in China and 20 mg elsewhere; and high dose atorvastatin = 20 mg in China and 80 mg elsewhere.

^†^ Participants may have a history of more than 1 of these conditions.

^‡^ Waist:hip ratio categorised as follows: low (<0.87 in women; <0.94 in men); medium (≥0.87 <0.93 in women; ≥ 0.94 < 1.00 in men); high (≥0.93 in women; ≥1.00 in men).

^§^ Estimated using the CKD EPI formula.^3^

^‖^ Urinary albumin:creatinine ratio categorised as follows: normo-albuminuria (<26.5 mg/g [<3 mg/mmol]); micro-albuminuria (≥26.5 <265 mg/g [≥3 <30 mg/mmol]); and macro-albuminuria (≥265 mg/g [≥30 mg/mmol]).

## Supplementary Table VI: Baseline LDL cholesterol among randomized participants

| **Region** | **Atorvastatin dose** | **LDL cholesterol (mmol/L)** | | | | | | |
| --- | --- | --- | --- | --- | --- | --- | --- | --- |
|  |  | **<0.5** | **≥0.5 <1.0** | **≥1.0 <1.5** | **≥1.5 <2.0** | **≥2.0 <2.5** | **≥2.5** | **Any** |
| **Europe** | Low | 8 | 376 | 2557 | 3600 | 1274 | 119 | 7934 |
|  | High | 6 | 340 | 2484 | 3463 | 1263 | 172 | 7728 |
|  | **Any** | **14** | **716** | **5041** | **7063** | **2537** | **291** | **15662** |
|  |  | (0%) | (5%) | (32%) | (45%) | (16%) | (2%) | (100%) |
| **North America** | Low | 4 | 209 | 1105 | 1010 | 209 | 13 | 2550 |
|  | High | 0 | 281 | 1509 | 1268 | 269 | 21 | 3348 |
|  | **Any** | **4** | **490** | **2614** | **2278** | **478** | **34** | **5898** |
|  |  | (0%) | (8%) | (44%) | (39%) | (8%) | (1%) | (100%) |
| **China** | Low | 4 | 305 | 1850 | 1822 | 489 | 43 | 4513 |
|  | High | 2 | 333 | 1728 | 1618 | 370 | 55 | 4106 |
|  | **Any** | **6** | **638** | **3578** | **3440** | **859** | **98** | **8619** |
|  |  | (0%) | (7%) | (42%) | (40%) | (10%) | (1%) | (100%) |
| **Overall** | Low | 16 | 890 | 5512 | 6432 | 1972 | 175 | 14997 |
|  | High | 8 | 954 | 5721 | 6349 | 1902 | 248 | 15182 |
|  | **Any** | **24** | **1844** | **11233** | **12781** | **3874** | **423** | **30179** |
|  |  | (0%) | (6%) | (37%) | (42%) | (13%) | (1%) | (100%) |

Central laboratory assays of LDL cholesterol are not available for 270 participants.

% = number of participants in cell / number with measured LDL cholesterol in the region.

## Supplementary Table VII: Schedule of central laboratory procedures

| **Sample Collection** |  | **Randomization**  **visit** | **2 month**  **visit** | **Annual sample 1*** | **Annual sample 2*** | **Annual sample 3^†^** | **Annual sample 4*** | **Final visit** |
| --- | --- | --- | --- | --- | --- | --- | --- | --- |
| Pre-specified analysis | Lipid and lipoprotein profile**^‡^** | All | All | 5% | 5% | All | 5% | All |
|  | Lipoprotein (a) | All |  | 5% | 5% | All | 5% | >5% |
|  | HbA1c | All |  |  |  |  |  | All |
|  | Creatinine | All |  |  |  |  |  | All |
|  | Urinary Albumin:creatinine ratio | All |  |  |  |  |  | All |
| Long-term storage | Genetic material | All |  |  |  |  |  |  |
|  | Plasma | All | All | 5% | 5% | All | 5% | All |
|  | Serum | All | All | 5% | 5% | All | 5% | All |
|  | Urine | All |  |  |  |  |  | All |
|  |  |  |  |  |  |  |  |  |
| * Annual samples to be collected from 5% randomized participants annually. | | | | | | | | |
| **^†^** Annual sample 3 to be collected from 100% randomized participants when median follow-up is ~2 years. | | | | | | | | |
| **^‡^** Total, LDL and HDL cholesterol, triglycerides, apolipoproteins A1 and B. | | | | | | |  |  |
|  | | | | | | |  |  |

## Supplementary Figure 1: REVEAL trial design


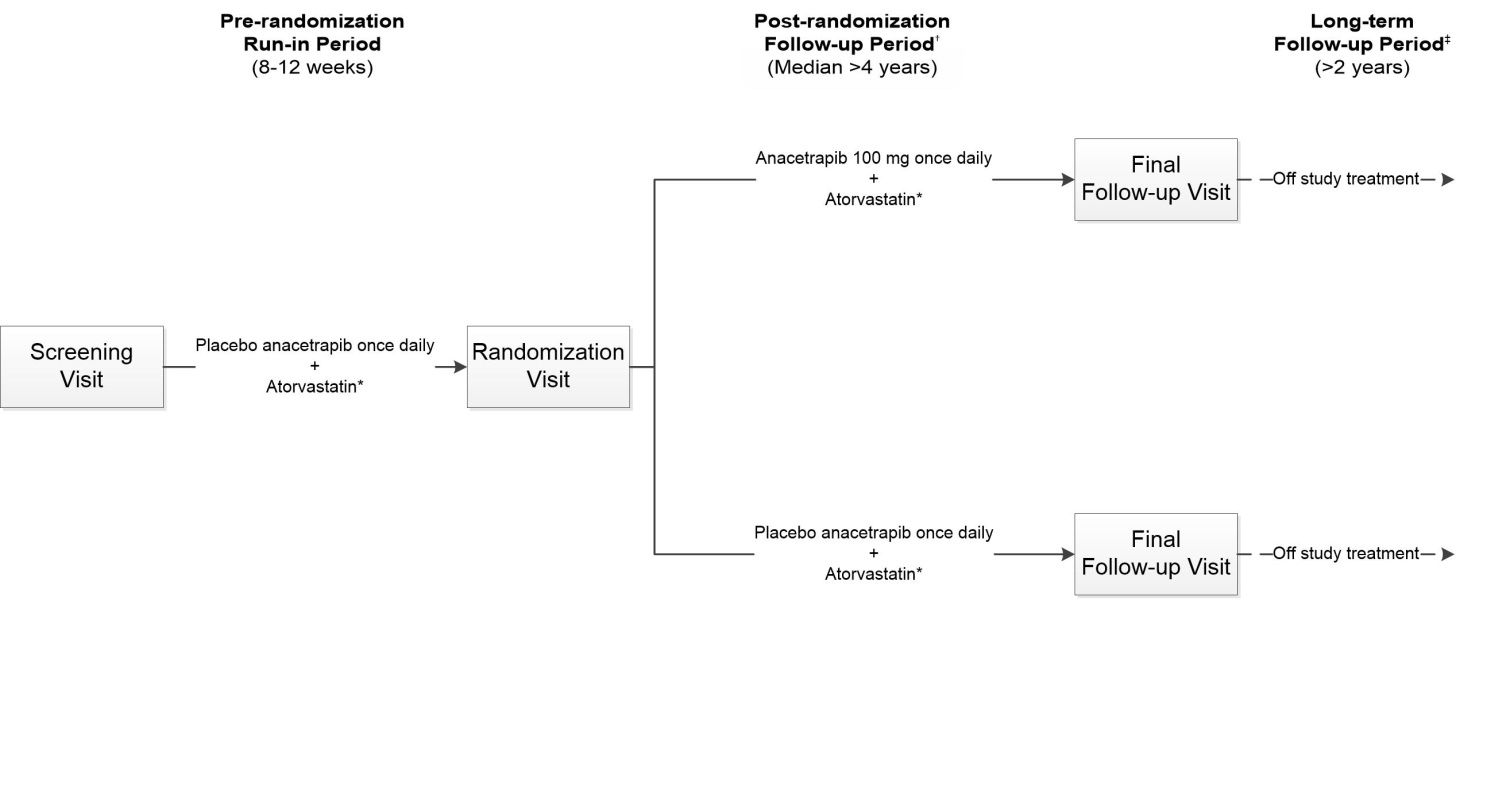


^*^ Atorvastatin dose: 10 mg or 20 mg once daily in China; 20 mg or 80 mg once daily in Rest of the World

^†^ Follow-up visits scheduled at 2 months, 4 months (China only), 6 months, and 6 monthly thereafter

^‡^ Long-term follow-up by 6 monthly telephone call, with linkage to routine medical records where possible

# Appendix A: Determination of atorvastatin dose at screening visit

1. Two doses of study atorvastatin available in each region:

High dose: 20 mg once daily in China, 80 mg once daily elsewhere

Low dose: 10 mg once daily in China, 20 mg once daily elsewhere

1. At the screening visit, the type and dose of current (pre-study) cholesterol-lowering treatment (statin and/or ezetimibe) is recorded and plasma total cholesterol is measured using a Reflotron Plus (Roche) dry chemistry analyser.
2. The extent to which each dose combination of statin and/or ezetimibe is anticipated to lower total cholesterol is shown in Table A.
3. Individuals are **NOT eligible** if the extent to which their current treatment lowers total cholesterol is greater than that for the high dose atovastatin in that region (see Table A):

- If CurrTreat > HighATV, then participant NOT eligible^[[1]](#footnote-1)^

[E.g., individuals taking rosuvastatin >20 mg as monotherapy, or ezetimibe in combination with simvastatin >20 mg, atorvastatin >20 mg, or rosuvastatin at any dose would not be eligible]

1. Individuals are **NOT eligible** if after changing from their current cholesterol treatment to high dose atorvastatin (for their region) total cholesterol would be unlikely to be <3.5 mmol/L:

- If TC x (1 – HighATV) / (1 – CurrTreat) ≥ 3.5, then participant NOT eligible^a^

[E.g., an individual in North America taking rosuvastatin 20 mg and with total cholesterol 3.9 mmol/L would not be eligible since: 3.9 x (1 – 0.45) / (1 – 0.40) = 3.58]

1. For the remaining **eligible** individuals, the **dose of study atorvastatin** is calculated as follows:

- If CurrTreat > LowATV, then participant to receive high dose atorvastatin^a^
- Else, if TC x (1 – LowATV) / (1 – CurrTreat) ≥ 3.5, then high dose atorvastatin^a^
- Else, low dose atorvastatin

[E.g., an individual in North America taking simvastatin 40 mg and with total cholesterol 3.5 mmol/L would receive low dose atorvastatin (20 mg) since: 3.5 x (1 – 0.33) / (1 – 0.31) = 3.40]

##

## Table A: Anticipated effect of pre-study lipid treatment on total cholesterol

| **Current statin** | **Current dose** | **Estimated reduction in total cholesterol*** | |
| --- | --- | --- | --- |
|  |  | **Statin only** | **Statin plus ezetimibe** |
| None | - | 0% | 13% |
| Pravastatin | ≤5mg | 8% | 19%^g^ |
|  | >5 ≤10 mg | 13% | 24% |
|  | >10 ≤20 mg | 18% | 27% |
|  | >20 ≤40 mg | 24% | *30%* |
|  | >40 ≤80 mg | *30%* | *33%* |
|  | >80 mg | **99%** | **99%** |
| Simvastatin | ≤5mg | 18% | *31%* |
|  | >5 ≤10 mg | 23% | 36% |
|  | >10 ≤20 mg | 28% | 41% |
|  | >20 ≤40 mg | *31%* | **99%** |
|  | >40 ≤80 mg | 36% | **99%** |
|  | >80 mg | **99%** | **99%** |
| Atorvastatin | ≤5mg | 25% | *34%* |
|  | >5 ≤10 mg | 29% | 38% |
|  | >10 ≤20 mg | *33%* | 39% |
|  | >20 ≤40 mg | *37%* | **99%** |
|  | >40 ≤80 | 45% | **99%** |
|  | >80 mg | **99%** | **99%** |
| Rosuvastatin | ≤2.5mg | *30%* | **99%** |
|  | >2.5 ≤5 mg | *33%* | **99%** |
|  | >5 ≤10 mg | 36% | **99%** |
|  | >10 ≤20 mg | 40% | **99%** |
|  | >20 ≤40 mg | **99%** | **99%** |
|  | >40 mg | **99%** | **99%** |
| Pitavastatin | ≤1mg | 23% | 36% |
|  | >1mg ≤2mg | 26% | 41% |
|  | >2mg ≤4mg | *33%* | **99%** |
|  | >4mg | **99%** | **99%** |
| Fluvastatin | ≤10mg | 15% | *31%* |
|  | >10mg ≤20mg | 17% | *31%* |
|  | >20mg ≤40mg | 19% | *31%* |
|  | >40mg ≤80mg | 25% | 36% |
|  | >80mg | **99%** | **99%** |

* Information is taken from Summary of Product Characteristics for Zetia, Zocor, Lipitor, Crestor, Lescol and Livazo. It is assumed that ezetimibe plus simvastatin ≥40 mg and ezetimibe plus rosuvastatin at any dose are more effective than atorvastatin 80 mg daily. Values of 99% (shown in **bold**) are used if the cholesterol-lowering effect is considered to be greater than that for atorvastatin 80 mg daily (rendering the participant ineligible worldwide); values underlined indicate estimated equivalent to atorvastatin >20 ≤80 mg (ineligible in China); and values in *italic* indicate equivalent to atorvastatin >10 ≤20 mg; other values (unmarked) are equivalent to atorvastatin <10 mg daily.

1. *TC = total cholesterol measured at Screening Visit using Reflotron Plus; HighATV = the expected reduction in total cholesterol with high dose atorvastatin for that region (compared with no treatment); LowATV = the expected reduction in total cholesterol with low dose atorvastatin for that region (compared with no treatment); and CurrTreat = the estimated reduction in total cholesterol achieved by their current cholesterol treatment; see Table A.* [↑](#footnote-ref-1)
