# Supplementary material for: Randomized Evaluation of the Effects of Anacetrapib through Lipid-modification (REVEAL)—A large-scale, randomized, placebo-controlled trial of the clinical effects of anacetrapib among people with established vascular disease: Trial design, recruitment, and baseline characteristics
Source: Am Heart J. 2017 May;187:182–90. doi: 10.1016/j.ahj.2017.02.021 (PMC5419667; doi:10.1016/j.ahj.2017.02.021)
Supplement: Supplementary file 2 — REVEAL Data Monitoring Committee Charter. [file mmc2.docx]

**Randomized EValuation of the Effects of Anacetrapib through Lipid-modification (HPS3/TIMI55 – REVEAL)**

**Data Monitoring Committee Charter**

**EDMS #2733**

Version 3.0: Revised Futility Criteria

(Effective: 3^rd^ November 2015)

**Table of Contents**

[1 Introduction 3](#_Toc432780720)

[1.1 Background 3](#_Toc432780721)

[2 Roles and Responsibilities 4](#_Toc432780722)

[3 Appointment and Membership 4](#_Toc432780723)

[3.1 DMC Members 4](#_Toc432780724)

[3.2 Ad Hoc DMC Advisors 4](#_Toc432780725)

[3.3 Conflicts of Interest 5](#_Toc432780726)

[3.4 Modifications to DMC Membership 5](#_Toc432780727)

[4 Specific Monitoring Responsibilities 5](#_Toc432780728)

[4.1 Periodic Review of Unblinded Data 5](#_Toc432780729)

[4.2 Ad Hoc Review of Suspected Serious Adverse Reactions 5](#_Toc432780730)

[4.3 Specific Safety Issues 5](#_Toc432780731)

[4.3.1 Liver effects 5](#_Toc432780732)

[4.3.2 Visit frequency in Asian participants 6](#_Toc432780733)

[4.3.3 Monitoring safety in patients with low baseline LDL cholesterol levels 6](#_Toc432780734)

[4.4 Review of Information from External Sources 6](#_Toc432780735)

[5 Early stopping rules 7](#_Toc432780736)

[5.1 For benefit 7](#_Toc432780737)

[5.1.1 Key subgroups 7](#_Toc432780738)

[5.2 For safety 8](#_Toc432780739)

[5.3 For futility 8](#_Toc432780740)

[5.4 Communication of early stopping recommendations 8](#_Toc432780741)

[6 Maintenance of Blinding 8](#_Toc432780742)

[7 Publications 8](#_Toc432780743)

[8 Indemnity 9](#_Toc432780744)

[9 Conduct of meetings 9](#_Toc432780745)

[9.1 Agenda 9](#_Toc432780746)

[9.2 Decision making 9](#_Toc432780747)

[9.3 Minutes and Archiving 9](#_Toc432780748)

[9.3.1 Minutes and meeting documents 9](#_Toc432780749)

[9.3.2 Secure disposal of meeting documents and closed session minutes 9](#_Toc432780750)

[9.4 Communication of DMC Meeting outcome 10](#_Toc432780751)

[10 APPENDIX: Key contact details 11](#_Toc432780752)

**Version History**

| **Version** | **Date** | **Summary** |
| --- | --- | --- |
| 1.0 | 19^th^ August 2011 | First released version. |
| 1.1 | 21^st^ February 2012 | Minor clarification of wording in relation to communication of early stopping recommendations |
| 2.0 | 4^th^ April 2014 | Changes to the criteria for stopping for futility. |
| 3.0 | 3^rd^ November 2015 | The formal interim review of efficacy by the Data Monitoring Committee, which was scheduled to take place at 2.5 years after median randomization, was cancelled and has been removed (section 5.1).  The review of futility at 2.5 years after median randomization that was introduced in version 2.0 of the DMC Charter was cancelled and has been removed (section 5.3).  The review of futility at 3.0 years after median randomization that was originally described in version 1.0 and subsequently modified in version 2.0 of the DMC Charter has been amended (section 5.3). |

# Introduction

Title: Randomized EValuation of the Effects of Anacetrapib through Lipid-

modification (HPS3/TIMI55 – REVEAL)

EUDRACT number: 2010-023467-18

ISRCTN number: 48678192

Sponsor: Clinical Trial Service Unit & Epidemiological Studies Unit (CTSU),

University of Oxford

Funder: Merck Sharp & Dohme (Merck)

## Background

REVEAL is a randomized trial investigating the effects of adding the CETP-inhibitor anacetrapib to effective LDL-lowering treatment with atorvastatin. Over 30,000 participants with pre-existing atherosclerotic vascular disease were randomized between anacetrapib 100 mg daily versus matching placebo, with scheduled median follow-up of about 4 years. At the initial Screening visit, eligible individuals were given Run-in medication consisting of placebo anacetrapib and active atorvastatin, and asked to return to the clinic in 8-12 weeks (see Figure). At the Randomization visit, eligible and consenting individuals were randomly allocated anacetrapib 100 mg or matching placebo, along with active atorvastatin at the same dose started at the Screening visit. The primary aim is to assess the effect of anacetrapib on the composite outcome of major events, defined as coronary death, myocardial infarction or coronary revascularization. Other aims include assessment of the safety of anacetrapib, as well as the effects on a number of other secondary or tertiary outcomes (as described in the protocol).

***Figure:*** *Outline of randomization and follow-up schedule*

The purpose of this document is to describe the roles and responsibilities of the independent Data Monitoring Committee (DMC) for the REVEAL trial, including the methods of providing information to and from the DMC, frequency and format of meetings, and statistical issues.

# Roles and Responsibilities

The independent DMC will act in an advisory capacity to the Steering Committee to review unblinded interim analyses of safety and efficacy of the study treatment in the REVEAL trial. Responsibilities of the DMC are:

1. to provide an independent overview of the safety of trial participants; and
2. to make recommendations about continuation, termination or other modifications to the trial based on their unblinded review of the study clinical outcome data.

The DMC will function independently of all other individuals and bodies associated with the REVEAL trial, including the Investigators, the Steering Committee, and the Funder.

# Appointment and Membership

## DMC Members

On the invitation of the Steering Committee Chair and the Principal Investigators, the following have agreed to serve as members of the DMC:

| **Name** | **Role** | **Location** | **Expertise** |
| --- | --- | --- | --- |
| Peter Sandercock | Chair | Edinburgh,  United Kingdom | Neurology (stroke medicine) /  Clinical trials |
| David DeMets | Member | Wisconsin,  USA | Clinical trials / biostatistics |
| John Kjekshus | Member | Oslo, Norway | Clinical trials / cardiology |
| James Neuberger | Member | Birmingham,  United Kingdom | Hepatology |
| Andrew Tonkin | Member | Melbourne,  Australia | Cardiology / Clinical trials |

Dr Jonathan Emberson (CTSU, Oxford) is the statistician to the DMC. He will be responsible for providing the unblinded report to the DMC and for drafting the minutes of its meetings.

## Ad Hoc DMC Advisors

If required by the DMC Chair, after discussion with other members of the DMC, experts in a particular field (e.g. liver adverse outcomes) may be asked to provide additional advice in confidence on specific aspects of the unblinded data. In such circumstances, these ad hoc advisors would typically be blinded to all other data (including primary and secondary study outcomes), unless the DMC Chair determines that it is necessary for them to be unblinded to any or all the other data in order to provide fully informed advice to the DMC. Not only would the content of such discussions with DMC Advisors be confidential, but every effort should be made to ensure that the fact that their advice has been sought also remains confidential.

## Conflicts of Interest

The DMC Members and Advisors must not otherwise be involved in the conduct of the REVEAL trial and must have no significant financial, scientific or other conflicts of interest with the Sponsor or the Funder. In particular, no DMC Member or Advisor should have a financial investment in the Sponsor, Funder or in any of the Funder’s competitors that would result in questions regarding his/her independence. Furthermore, no DMC Member or Advisor may consult for, be employed by, or enter into any future consulting or employment relationship with the Sponsor or the Funder while serving on the DMC, except that DMC Members or Advisors may (i) concurrently serve on other DMCs for the Sponsor or the Funder, and/or (ii) consult for the Sponsor or the Funder where the annual aggregate compensation for such non-promotional consulting services does not exceed $15,000 (US dollars) exclusive of any DMC compensation.

All potential DMC Members and Advisors will be vetted by the Sponsor and the Funder for significant conflicts of interest prior to their appointment, and should disclose any conflict of interest to the DMC Chair at the start of each meeting thereafter.

## Modifications to DMC Membership

Further DMC Members may be appointed if there are resignations from the current membership or at the request of the DMC Chair. All such appointments must be agreed by the Steering Committee Chair and the Principal Investigators. In addition, ad hoc DMC Advisors may be appointed by the DMC Chair, without consulting or informing the Steering Committee Chair, Principal Investigators, Sponsor or Funder. Any new DMC Member or Advisor would be subject to the terms of this Charter (including requirements for Conflicts of Interest; see section 3.3).

# Specific Monitoring Responsibilities

## Periodic Review of Unblinded Data

During the study, interim analyses of all Serious Adverse Events (SAEs) and other study outcomes (both overall and in key subgroups; see section 5.1.1) will be supplied in strict confidence to the independent DMC. It will request such analyses at a frequency relevant to the stage of the study (typically at 6-12 monthly intervals, with a Chairman’s review typically every 3-6 months) or in response to emerging data from other trials. Additional meetings of the DMC may be called at any time by the Chair or at the request of the Steering Committee Chair and Principal Investigators. Unblinded analyses will include:

- Primary, secondary and tertiary assessments
- Additional safety assessments (as specified in the protocol)
- Analyses of biochemistry (blood lipids and lipoproteins)

## Ad Hoc Review of Suspected Serious Adverse Reactions

Individual unblinded reports of each Suspected Serious Adverse Reaction (SSAR) will be provided to the DMC Chair by Central Coordinating Office clinicians.

## Specific Safety Issues

### Liver effects

During 18 months of follow-up in the DEFINE study (conducted chiefly in Caucasians), 1 patient had consecutive liver transaminase >3x upper limit of normal (ULN) among about 800 patients allocated anacetrapib 100mg daily versus 8 cases among about 800 patients allocated placebo. In a smaller trial in Japan, such liver enzyme elevations were seen in 2 of 80 patients allocated anacetrapib 100mg daily and 3 of 80 allocated anacetrapib 300 mg daily, but in none of the 80 patients allocated placebo.

Both the favourable trend in the DEFINE study and the adverse trend in the Japanese study may be due to chance, but the potential for liver adverse effects will be monitored carefully in the REVEAL trial. In particular, the DMC will monitor unblinded information on various potential signals for liver safety, including:

- Alanine transaminase (ALT; >3x ≤5x ULN; >5x ≤10x ULN; >10x ≤20x ULN; and >20x ULN)
- ALT >3x ULN plus bilirubin >2x ULN plus CK ≤5x ULN
- Liver-related serious adverse events (such as liver failure and hepatitis)

Decisions and recommendations will generally be based on analyses restricted to events for which there is no likely alternative diagnosis (e.g. viral, alcohol or inflammatory liver disease) and that are plausibly related to study treatment. All liver safety information will be considered both in the trial overall, and separately among patients from Asia and from the other participating countries.

### Visit frequency in Asian participants

All participants are scheduled to have follow-up visits after randomisation at 2 months, 6 months and then 6 monthly thereafter, with additional “Early Recall” visits as required to address potential safety concerns. Since experience with anacetrapib in Asian patients is currently more limited than in Western populations, an extra follow-up visit at 4 months is planned for the first few thousand patients randomized in Asia. The DMC will review the liver safety data when at least 4000 Asian participants have attended a 2 month follow-up visit and at least 2000 Asian participants have attended a 4 month follow-up visit. It will then advise if it considers that the follow-up schedule may revert to being the same as in the rest of the world (for example, if there was no marked excess of ALT >3x ULN in the anacetrapib group).

### Monitoring safety in patients with low baseline LDL cholesterol levels

Although there are theoretical concerns about the safety of very low LDL cholesterol levels, there is no direct evidence of a lower threshold below which lowering of LDL cholesterol is hazardous. Nevertheless, there is a clear need to obtain direct randomized evidence of the safety and efficacy of using anacetrapib in individuals on intensive statin regimens. It is anticipated that around 9% of those allocated to anacetrapib and <1% of those allocated to placebo will have LDL cholesterol <0.5 mmol/L, with 80% and 9% having LDL cholesterol between 0.5 and 1.0 mmol/L, respectively. Therefore, in addition to monitoring overall safety, an evaluation of safety by baseline LDL cholesterol levels will be performed. This will allow review of safety (and efficacy) information among around 2000 individuals with a randomization LDL cholesterol <1.25 mmol/L, and will provide controlled safety information on the clinical effects of lowering LDL cholesterol from a mean of about 1.0 mmol/L to about 0.6 mmol/L.

## Review of Information from External Sources

During the REVEAL trial, there may be new information pertaining to the safety and efficacy of the study treatments, including updates to the Confidential Investigator Brochure or results from other trials of the same or related treatments. The Principal Investigators will review any such information and make a written assessment for consideration by the DMC of whether, in its view: (i) any changes should be made to the trial protocol; (ii) further information should be provided to investigators or participants; and/or (iii) participants should be re-consented.

# Early stopping rules

## For benefit

In the light of the interim analyses of unblinded data and any other information considered relevant, the DMC will advise the Steering Committee if, in its view, the randomized comparisons in the study have provided **both** (i) “proof beyond reasonable doubt” that prolonged use of anacetrapib reduces the primary outcome of major coronary events (with consistent results in key subgroups; see section 5.1.1), as well as coronary death or myocardial infarction (i.e. the primary outcome minus cardiovascular revascularization) and cardiovascular mortality, with a consistent effect on all-cause mortality; **and** (ii) evidence that might reasonably be expected to influence materially patient management by many clinicians who are already aware of the results of other relevant trials. Appropriate criteria of proof beyond reasonable doubt cannot be specified precisely, but in general to justify halting, or modifying, the study prematurely for benefit it is expected that the guidelines set out in Table 1 would be fulfilled. This approach, which will be applied at each review of the emerging data, has the practical advantage that the exact number of interim analyses is of little importance.^^[[1]](#footnote-1)^^ Currently, there is only 1 planned formal interim analysis for early stopping for efficacy and it will take place when at least 3.0 years median follow-up have been achieved.

***Table 1:*** *Guidelines for early stopping for efficacy*

| **Median follow-up** | **Outcome** | **Stopping criteria** |
| --- | --- | --- |
| **≥3.0 years** | Primary outcome | ≥3 SD (with consistent results in key subgroups) |
|  | Coronary death or MI | ≥2.6 SD overall plus ≥2.0 SD for at least one component |
|  | Cardiovascular mortality | ≥2.0 SD |
|  | Total mortality | Directionally consistent effect |

SD = standard deviations

### Key subgroups

For the purposes of DMC early stopping rules, treatment effects are to be assessed in the following key subgroups defined at the randomization visit:

- history of diabetes mellitus (i.e. yes; no)
- HDL cholesterol (i.e. three similar-sized groups);
- LDL cholesterol (i.e. three similar-sized groups);
- age (i.e. <65; ≥65 years);
- sex (i.e. male; female); and
- region (i.e. North America; Asia; Other).

A recommendation to stop early for benefit should only be made if the results seen in key subgroups (such as those listed above) are generally consistent with the beneficial results seen in the whole population. If, in the view of the DMC, the evidence is not sufficiently convincing in one or more of the key subgroups (i.e. the current evidence would not be expected to influence materially patient management if clinicians were made aware of the results), then the DMC would not be expected to recommend stopping the trial for efficacy.

## For safety

The DMC is expected to advise the Steering Committee if clear evidence emerges of an adverse effect on all-cause mortality or on cardiovascular mortality of at least 2 standard deviations (unless there are some mitigating circumstances, such as small numbers of deaths or inconsistent results for fatal and non-fatal events) or if, in the view of the DMC, there is other compelling evidence of hazard that seems likely to outweigh any potential benefit.

## For futility

In formulating their opinions on the possible benefits and safety of anacetrapib, the DMC should consider whether continuing the trial is likely to demonstrate any clinically meaningful effects of anacetrapib. For example, a positive result at 4.0 years median follow-up is unlikely if at 3 years median follow-up and at least 70% of the anticipated total number of primary and secondary events, (i) the hazard ratio for the primary endpoint is greater than 0.95, (ii) there is no evidence of a treatment effect for those patients with >3 years follow-up, and (iii) there are no promising results for any major subgroup (e.g. diabetics, low baseline HDL-cholesterol) or for any major outcome (e.g. coronary death, myocardial infarction or presumed ischaemic stroke). However, the DMC must also consider the possibility that greater benefits might emerge with prolonged follow-up. (For example, the benefits of statin therapy in the first year of treatment are about half those seen in each subsequent year of treatment).

## Communication of early stopping recommendations

If the DMC concludes that either the safety, efficacy or futility criteria for recommending early stopping have been achieved, the DMC Chair will immediately advise the Steering Committee Chair. In these circumstances, the Steering Committee Chair will first discuss the recommendation with the Steering Committee Deputy Chair and Principal Investigators (seeking any further information from the DMC or other sources considered necessary) before convening a meeting or teleconference of the Steering Committee to consider what action (if any) to take.

# Maintenance of Blinding

Physical, electronic and procedural measures will be put in place to maintain the confidentiality of all information relating to unblinded analyses. Unless advised by the DMC in response to clear evidence of benefit or hazard, the Sponsor, Steering Committee, and all investigators, participants, representatives of the Funder, and study staff (except those who provide the confidential analyses to the DMC) will remain blind to the interim results on mortality and major morbidity until the end of the study. The only exception to this is in the assessment and reporting of SSARS, which will be handled as described in the protocol.

# Publications

The Steering Committee will be responsible for drafting the main reports from the study and for review of any other reports. DMC Members will be given the opportunity to provide comments on the main results manuscript prior to its submission for publication.

# Indemnity

The University of Oxford will indemnify the DMC members against any claims or legal actions for performing the role outlined in the DMC Charter. The details of the indemnity will be outlined in detail in the agreements between the University and the DMC Member (or their institution).

# Conduct of meetings

## Agenda

| **Session** | **Present** | **Content** |
| --- | --- | --- |
| **Open*** | DMC members  DMC statistician  Steering Committee Chair and Deputy Chair  Principal Investigators  Study statistician | Principal Investigators to report on:   - Data quality, and completeness of follow-up and adjudication - Any relevant new external evidence (especially results from other relevant ongoing trials) - Any proposals for changes in the study protocol |
| **Closed** | DMC members  DMC statistician | - DMC Chair to review conflict of interest statements from all DMC members - DMC statistician to report on unblinded data on efficacy and safety - DMC to formulate recommendations |
| **Open*** | DMC members  DMC statistician  Steering Committee Chair and Deputy Chair  Principal Investigators  Study statistician | - Principal Investigators to clarify any issues raised - Discussion of any action items - Set date of next meeting |

* No unblinded information is to be presented or discussed during the Open sessions.

## Decision making

In general, DMC decisions should be made by consensus, but where this is not possible, decisions will require a simple majority of the voting members of the DMC present at the meeting. For the avoidance of doubt, the DMC Statistician and any ad hoc advisors are non-voting.

## Minutes and Archiving

### Minutes and meeting documents

Unless otherwise determined by the DMC Chair, minutes for the Open and Closed sessions will be taken by the DMC Statistician. The DMC Chair will be responsible for the accuracy of the minutes, and for their secure storage until the end of the trial. Meeting documents (including closed session minutes) will only be distributed by secure means (e.g. encrypted and password-protected electronic files). These will remain confidential until the end of the trial, at which time they will be archived by the Sponsor and may be made available for public scrutiny (e.g. by regulatory authorities).

### Secure disposal of meeting documents and closed session minutes

Any hard copy meeting documents or closed session minutes must be disposed of securely after each meeting. Each DMC Member will be responsible for either returning these to the statistician for secure disposal, or for shredding the documents themselves and notifying the unblinded statistician that they have done so.

## Communication of DMC Meeting outcome

Within 2 weeks after each meeting, the DMC Chair will provide the Steering Committee Chair with a letter stating the general outcome of the meeting and any recommendations. For example, this letter may simply contain the statement that the trial should continue as planned and give the planned date for the next DMC meeting. Unless indicated otherwise by the DMC Chair, this letter will not be considered confidential. In accordance with the study protocol, the DMC is responsible for providing advice to the Steering Committee. The Steering Committee is responsible for reviewing this advice and, if necessary, agreeing to changes to the Protocol (including stopping early for either benefit or for safety).

# APPENDIX: Key contact details

**Sponsor and Central Coordinating Office**

Clinical Trial Service Unit & Epidemiological Studies Unit (CTSU)

Richard Doll Building

University of Oxford

Old Road Campus

Roosevelt Drive

Oxford OX3 7LF

United Kingdom

[reveal@ctsu.ox.ac.uk](mailto:reveal@ctsu.ox.ac.uk)

Tel: +44 (0) 1865 743743

Fax: +44 (0) 1865 743873

1. Peto R, Pike MC, Armitage P, Breslow NE, Cox DR, Howard SV, et al. Design and analysis of randomized clinical trials requiring prolonged observation of each patient. II. analysis and examples. Br J Cancer. 1977;35:1-39. [↑](#footnote-ref-1)
