# Supplementary material for: Randomized Evaluation of the Effects of Anacetrapib through Lipid-modification (REVEAL)—A large-scale, randomized, placebo-controlled trial of the clinical effects of anacetrapib among people with established vascular disease: Trial design, recruitment, and baseline characteristics
Source: Am Heart J. 2017 May;187:182–90. doi: 10.1016/j.ahj.2017.02.021 (PMC5419667; doi:10.1016/j.ahj.2017.02.021)
Supplement: Supplementary file 3 — REVEAL Study Protocol. [file mmc3.docx]

**HPS 3 / TIMI 55: REVEAL (Randomized EValuation of the Effects of Anacetrapib through Lipid-modification):**

**A large-scale, randomized placebo-controlled trial of the clinical effects of anacetrapib among people with established vascular disease**

**Does inhibition of cholesteryl ester transfer protein (CETP) with anacetrapib prevent vascular events in high-risk patients who are receiving intensive LDL cholesterol lowering treatment?**

Large-scale randomized trials have shown that lowering LDL cholesterol by about 1 mmol/L for 4-5 years reduces the risks of coronary events and of strokes by about one fifth. Furthermore, recent trials assessing more intensive versus standard statin regimens have demonstrated additional benefit with more intensive lowering of LDL cholesterol. Nevertheless, among individuals with a history of vascular disease or diabetes, cardiovascular risk remains elevated even after several years of intensive LDL-lowering treatment. For example, in 2 recent trials, over 10% of coronary heart disease (CHD) patients still suffered a major cardiovascular event during 4-5 years of intensive statin therapy.

Observational studies indicate that lower concentrations of HDL cholesterol and of apolipoprotein (apo) A1 are associated with an elevated risk of CHD events. Cholesteryl ester transfer protein (CETP) facilitates the exchange of cholesteryl esters for triglycerides between HDL particles and apo B containing particles. CETP inhibition leads to increases in HDL cholesterol and apo A1 concentrations, and reductions in LDL cholesterol, apo B and triglyceride concentrations. However, the only reported outcome trial of a CETP inhibitor – torcetrapib – was stopped early: an unexpected excess risk of cardiovascular events and deaths was observed. It is unclear whether this was due to off-target increases in systemic blood pressure and plasma aldosterone levels induced by torcetrapib, or if it was attributable to some other effect of the drug or CETP inhibition.

When used either as monotherapy or in combination with a statin, the CETP inhibitor anacetrapib increases HDL cholesterol and apo A1 concentrations by about 140% and 45% respectively, and reduces LDL cholesterol and apo B concentrations by about 30-40%. Anacetrapib has been well tolerated in early phase studies and, importantly, has no effects on blood pressure or aldosterone levels. The lipid changes that are produced by anacetrapib might well reduce the risk of vascular events substantially, but there is a clear need for large-scale randomized evidence to assess any benefits and hazards reliably.

**A streamlined international trial**

The present study will compare anacetrapib 100 mg once daily versus matching placebo in around 30,000 participants with pre-existing atherosclerotic vascular disease who are also receiving effective LDL-lowering therapy using atorvastatin. Follow-up for about 4 years will allow reliable assessments of any beneficial or adverse effects of anacetrapib on mortality and major morbidity. This important international study will be coordinated by the Central Coordinating Office at the University of Oxford (which will act as the trial Sponsor), with Regional Coordinating Centres responsible for the conduct of the trial in about 400 hospitals worldwide. The study design is streamlined: extra work for collaborating doctors and hospitals has been kept to a minimum, and only essential data will be collected directly using bespoke user-friendly computer-based systems.

Central Coordinating Office, Clinical Trial Service Unit,

Richard Doll Building, Old Road Campus, Roosevelt Drive, Oxford OX3 7LF, UK

Tel: +44(0)1865 743882, fax: +44 (0)1865 743988

**Version History**

| **Version** | **Date** | **Summary** |
| --- | --- | --- |
| 1.0 | 15 October 2010 | First released version. |
| 1.1 | 25 May 2012 | Minor changes and updates to Steering Committee membership. |
| 2.0 | 11 January 2016 | The Steering Committee (which remains blind to analyses of the study results by treatment allocation) has agreed the following changes to the protocol:   1. Cancel any formal interim review of efficacy by the Data Monitoring Committee prior to 3 years’ median follow-up. (The interim review of efficacy originally scheduled to take place at 2.5 years after median randomization, was cancelled and has been removed from the protocol; section 2.5.2.2). 2. Revise the secondary assessments (section 2.3.1.2) to: 3. Major atherosclerotic events (defined as coronary death, myocardial infarction or presumed ischaemic stroke; the key secondary outcome); 4. Presumed ischaemic stroke; and 5. Major vascular events (defined as coronary death, myocardial infarction, coronary revascularization or presumed ischaemic stroke); 6. Make minor changes to the tertiary assessments (section 2.3.1.4)   Power calculations for the new secondary endpoints have been added, based on the number of participants who have been randomized and the blinded event rates observed to date (section 2.4.3).  A brief summary of relevant information that has emerged since the study background and rationale (section 1.1) were written has been added (section 1.2).  A small number of administrative changes and clarifications have also been made. |

**Table of contents**

1 BACKGROUND AND RATIONALE 5

1.1 Does inhibition of cholesteryl ester transfer protein (CETP) prevent major vascular events in patients with vascular disease? 5

1.1.1 Substantial residual cardiovascular risk even with intensive LDL-lowering treatment 5

1.1.2 Higher blood concentrations of HDL cholesterol and of apolipoprotein A1 are associated with lower cardiovascular risk throughout the usual range 5

1.1.3 The clinical effects of currently available treatments for raising HDL cholesterol remain uncertain 6

1.1.4 Inhibition of cholesteryl ester transfer protein as a possible therapeutic target 6

1.1.5 Clinical safety and effectiveness of CETP inhibition is currently uncertain 7

1.1.6 Rationale for intensive background LDL cholesterol-lowering 8

1.2 Brief Summary of New Information Since Protocol Version 1 9

2 PLAN OF INVESTIGATION 10

2.1 Study Aims 10

2.2 Treatment Comparisons 10

2.2.1 Run-in period prior to randomization 10

2.2.2 Randomization to anacetrapib versus placebo 10

2.3 Data Analysis Plan 11

2.3.1 Main and subsidiary analyses 11

2.3.1.1 Primary assessment 11

2.3.1.2 Secondary assessments 11

2.3.1.3 Additional assessments requested by regulatory agencies 11

2.3.1.4 Tertiary assessments 11

2.3.1.5 Additional safety assessments 12

2.3.1.6 Analyses of biochemical efficacy 13

2.3.1.7 Exploratory assessments 13

2.3.1.8 Health economic assessments 13

2.3.2 Statistical analysis 13

2.4 Sample size and predicted number of events 14

2.4.1 Initial assumptions (prior to study start) 14

2.4.2 Statistical power (prior to study start) 15

2.4.3 Revised statistical power (protocol version 2.0) 16

2.4.4 Planned study duration 17

2.5 Data and Safety Monitoring 17

2.5.1 Recording and reporting of adverse events 17

2.5.1.1 Definition of Serious Adverse Events (SAE) 17

2.5.1.2 Reporting of Serious Adverse Events and other relevant events 17

2.5.1.3 Reporting of Suspected Serious Adverse Reactions (SSARs) 18

2.5.2 Interim analyses: role of the Data Monitoring Committee 18

2.5.2.1 Frequency of reviews 18

2.5.2.2 Early stopping for benefit 19

2.5.2.3 Early stopping for safety 19

2.6 Central and Regional Coordination of Local Clinical Centres 20

2.6.1 Training and monitoring 20

2.6.2 Supply of study materials 20

2.6.3 Data management 20

2.6.4 Biological sample assay, transport and storage 21

2.6.4.1 Local analysis of eligibility and safety bloods 21

2.6.4.2 Central assessment of samples collected at the randomization visit and during follow-up 21

2.6.4.3 Consent approval for unspecified analyses on blood and urine samples 21

2.6.5 Administrative details 22

2.6.5.1 Source documents and archiving 22

2.6.5.2 Sponsor and funding 22

2.6.5.3 Indemnity 22

2.6.5.4 End of trial 22

2.6.5.5 Publications, reports and substudies 22

3 SUMMARY OF PRACTICAL PROCEDURES 23

3.1 Eligibility For the Study 24

3.1.1 Inclusion criteria 24

3.1.2 Exclusion criteria 24

3.2 Screening Visit and Pre-randomization Run-in 25

3.2.1 Assessment of relevant medical history and eligibility 25

3.2.2 Invitation to participate and standardization of LDL-lowering therapy 25

3.2.3 Review of eligibility and LDL cholesterol control by each individual’s own doctor 26

3.3 Randomization Visit (0 months) 26

3.3.1 Final check of eligibility and compliance before randomization 26

3.3.2 Collection of blood and urine samples 27

3.3.3 Random allocation of study treatment 27

3.4 Follow-up Visits (2 and 6 months and then 6-monthly) 27

3.4.1 Recording adverse events and compliance 27

3.4.2 Collection of blood and urine samples 28

3.4.3 Issuing study treatment and arranging further appointments 28

3.4.4 Follow-up for randomized participants not attending study clinics 28

3.4.5 Follow-up of randomized participants via electronic healthcare systems and other methods during and after the scheduled follow-up period 28

3.5 Early Recall Visits and Modifying Study Treatment 29

3.5.1 Monitoring significant biochemical or other problems 29

3.5.1.1 Monitoring elevated liver transaminases 29

3.5.1.2 Monitoring elevated creatine kinase 30

3.5.2 Modifying study treatment 30

3.5.2.1 Discontinuation of anacetrapib (or placebo) and LDL-lowering therapy 30

3.5.2.2 Requirement for changes to LDL-lowering treatment 31

3.5.2.3 Additional monitoring for participants receiving oral anticoagulants 31

3.5.3 Unblinding of study treatment 31

3.5.4 Withdrawal of consent 32

3.6 Confirmation and Verification of Study Outcomes 32

4 REFERENCES 33

5 Appendices 36

5.1 Appendix 1: Organisational Structure and Responsibilities 36

5.2 Appendix 2: Visit schedule and procedures 39

5.2.1 Clinic procedures 39

5.2.2 Central laboratory procedures 40

5.3 Appendix 3: Study Investigators 41

1. BACKGROUND AND RATIONALE

## Does inhibition of cholesteryl ester transfer protein (CETP) prevent major vascular events in patients with vascular disease?

### Substantial residual cardiovascular risk even with intensive LDL-lowering treatment

Large-scale randomized trials have shown that lowering LDL cholesterol concentration by about 1 mmol/L for 4-5 years reduces the risks of coronary events (including myocardial infarction [MI], coronary death and revascularization procedures) and of strokes by about one fifth.[^1^](#_ENREF_1) Furthermore, recent trials have demonstrated that more intensive lowering of LDL cholesterol produces additional reductions in the risk of such events.[^2^](#_ENREF_2) Nevertheless, among individuals with vascular disease or diabetes, cardiovascular risk remains elevated even after some years of intensive LDL-lowering treatment. For example, in two recent trials, over 10% of patients with coronary heart disease (CHD) suffered a major cardiovascular event during 4-5 years of intensive statin therapy.[^3^](#_ENREF_3)^,^ [^4^](#_ENREF_4)

### Higher blood concentrations of HDL cholesterol and of apolipoprotein A1 are associated with lower cardiovascular risk throughout the usual range

HDL cholesterol is inversely associated with CHD risk.[^5-7^](#_ENREF_5) In the Prospective Studies Collaborative (PSC) meta-analysis of 60 observational studies involving 3000 deaths from CHD among 154,000 people, both higher HDL cholesterol and lower non-HDL cholesterol levels were associated with lower CHD risk, and these effects were approximately independent in multiplicative terms (Figure 1).[^8^](#_ENREF_8) Each 0.33 mmol/L (13 mg/dL) higher HDL cholesterol was associated with about one-third lower risk of CHD death.

***Figure 1.*** *(a) Inverse association between HDL cholesterol and subsequent risk of CHD death at both higher (≥5 mmol/L; 200 mg/dL) and lower levels of non-HDL cholesterol; and (b) Positive association between non-HDL cholesterol and subsequent risk of CHD death at both higher (≥1.25 mmol/L; 50 mg/dL) and lower levels of HDL cholesterol.*[*^8^*](#_ENREF_8)

HDL particles vary considerably in size and cholesterol content, and may also vary in their biological activity. Small HDL particles, which are more numerous, typically carry only 1-2 molecules of apo A1 and a few dozen cholesterol molecules, while large ones typically carry 3-4 molecules of apo A1 and over 100 cholesterol molecules. Analyses from the ISIS case-control study[^9^](#_ENREF_9) (which involved 3500 MI patients and 9800 controls) found that apo A1 and apo B were both strongly predictive of MI risk, as would be anticipated from the associations of HDL and non-HDL cholesterol (Figure 1). In the Emerging Risk Factors Collaborative (ERFC) meta-analysis of prospective studies, 1 standard deviation (SD) higher apo A1 (29 mg/dL) or HDL-cholesterol (0.38 mmol/L; 15 mg/dL) was associated with between one-fifth and one-quarter lower risk of CHD.[^10^](#_ENREF_10)

The association between stroke risk and HDL cholesterol or apo A1 is much less clear. Neither the PSC (1000 stroke deaths among 154,000 individuals)[^8^](#_ENREF_8) nor the Asia Pacific Studies Collaboration (850 fatal or non-fatal strokes among 80,000 individuals)[^11^](#_ENREF_11) found any significant association between HDL cholesterol and stroke risk. In the ERFC meta-analysis, the hazard ratio for stroke (after adjustment for potential confounding factors) was 0.96 (95% CI 0.90-1.02) per 1 SD higher HDL cholesterol and 0.97 (95% CI 0.88-1.08) per 1 SD higher apo A1.[^10^](#_ENREF_10) There are limited data on the associations between lipid fractions or apolipoprotein concentrations and the risks of particular stroke subtypes. In the AMORIS cohort (6000 fatal or non-fatal strokes among 149,000 individuals), both HDL cholesterol and apo A1 were inversely associated with the risk of ischaemic, but not haemorrhagic, stroke.[^12^](#_ENREF_12)

### The clinical effects of currently available treatments for raising HDL cholesterol remain uncertain

Randomized trial evidence for beneficial effects from raising HDL cholesterol is limited. Most previous trials have been performed using fibrates, which raise HDL cholesterol only modestly (5-10%), and those studies have produced mixed results.[^13-17^](#_ENREF_13) When used with a statin, niacin 2g daily increases HDL cholesterol by about 20% and apolipoprotein A1 by about 7%, and reduces LDL cholesterol by about 18%.[^18^](#_ENREF_18) In the Coronary Drug Project (which was initiated in the 1960s, before the advent of statins), niacin resulted in a significant reduction of about one-fifth in non-fatal MI or coronary death during the 5-8 year scheduled treatment period.[^19^](#_ENREF_19) However, widespread use of this agent has been limited by poor tolerability (mainly due to flushing produced by cutaneous vasodilatation), and it is not known whether any benefits are additional to those of statin therapy. The combination of extended release niacin with laropiprant (a selective prostaglandin D receptor antagonist that reduces niacin-induced flushing) is being investigated in the ongoing HPS2-THRIVE trial among 25,000 patients with pre-existing atherosclerotic disease, all of whom are receiving simvastatin 40 mg daily plus (if required for adequate control of LDL cholesterol) ezetimibe 10 mg daily.[^20^](#_ENREF_20)

### Inhibition of cholesteryl ester transfer protein as a possible therapeutic target

Cholesteryl ester transfer protein (CETP) is a plasma protein that facilitates the exchange of cholesteryl esters and triglycerides between HDL particles and the atherogenic apolipoprotein B-containing particles. Human genetic mutations currently known to be associated with reduced CETP activity produce mild elevations in HDL cholesterol and apo A1 (about 3-5% per allele), have negligible effects on LDL cholesterol and apo B concentrations, and are associated with a small reduction in cardiovascular risk (about 5% per allele).[^21^](#_ENREF_21) Pharmacological inhibition of CETP produces more marked increases in HDL cholesterol and apo A1, along with reductions in LDL cholesterol and apo B. The magnitude of these effects varies significantly between the three CETP inhibitors that have been developed (Table 1).

***Table 1.*** *Changes* in blood lipid and lipoprotein concentrations with CETP inhibitors*

|  | Torcetrapib | Dalcetrapib | Anacetrapib[^22^](#_ENREF_22) | |
| --- | --- | --- | --- | --- |
|  | 60 mg daily[^23^](#_ENREF_23) | 600 mg daily[^24^](#_ENREF_24) | 40 mg daily | 150 mg daily |
| Total cholesterol | 4% | n/a | 1% | 3% |
| LDL cholesterol | -24% | -4% | -27% | -40% |
| Triglycerides | -9% | -3% | -11% | -11% |
| Apolipoprotein B | -12% | n/a | -20% | -29% |
| HDL cholesterol | 61% | 25% | 86% | 139% |
| Apolipoprotein A1 | 25% | 10% | 32% | 47% |

* Negative values indicate reductions; n/a = not available

### Clinical safety and effectiveness of CETP inhibition is currently uncertain

Torcetrapib was the first CETP inhibitor to be studied in a large-scale outcome trial. The ILLUMINATE trial randomized 15,000 participants at high cardiovascular risk to receive torcetrapib 60 mg daily versus placebo in addition to background LDL-lowering therapy with atorvastatin aiming to achieve LDL cholesterol <100 mg/dL.[^23^](#_ENREF_23) Acting on the advice of the Data Monitoring Committee, the trial was stopped prematurely because of an excess of deaths among those allocated torcetrapib (93 [1.2%] vs. 59 [0.8%]; HR 1.58, 95% CI 1.14-2.19; p=0.006). The mortality result could not be clearly attributed to any particular cause of death, but there was a very definite increase in cardiovascular events (464 [6.2%] vs. 373 [5.0%]; HR 1.25; 95% CI 1.09-1.44; p=0.001). Imaging trials of torcetrapib provided no clear evidence of any effects (adverse or beneficial) on arterial wall markers of atherosclerosis.[^25-27^](#_ENREF_25) Torcetrapib was found to increase systolic blood pressure by about 5 mmHg, as well as producing increases in blood sodium, bicarbonate and aldosterone concentrations. It seems likely that these off-target effects may have contributed to the observed hazard, but it remains unclear whether that is the entire explanation, or whether CETP inhibition itself played a role.

Dalcetrapib has more modest effects on HDL cholesterol than torcetrapib or anacetrapib (Table 1). The Dal-Outcomes study aims to assess the effect of dalcetrapib on the risk of major vascular events among about 15,000 patients with recent acute coronary syndrome. Recruitment started in 2008 and results are anticipated in 2013.[^28^](#_ENREF_28)

Anacetrapib is an orally-active CETP inhibitor which has been well tolerated in early phase studies and, importantly, has no discernible effect on resting or ambulatory blood pressure or on plasma aldosterone concentrations.[^22^](#_ENREF_22)^,^ [^29^](#_ENREF_29)^,^ [^30^](#_ENREF_30) It has a greater impact on the lipid and lipoprotein profile than either torcetrapib or dalcetrapib, producing increases in HDL cholesterol and apo A1 concentrations of about 140% and 45%, respectively, and lowering LDL cholesterol and apo B by about 30-40% at a dose of 150 mg daily (Table 1). It is now being developed at a dose of 100 mg daily,[^30^](#_ENREF_30) which has similar effects on the lipid profile to those seen with 150 mg daily. The present study aims to compare anacetrapib 100 mg once daily versus placebo for about 4 years among at least 30,000 patients with pre-existing atherosclerotic vascular disease who are also receiving effective LDL-lowering therapy using atorvastatin. This will allow reliable assessment not only of the impact of anacetrapib on the risk of major vascular events but also of its safety.

### Rationale for intensive background LDL cholesterol-lowering

There is now reliable evidence that intensive LDL-lowering treatment with a higher dose of a statin reduces the risk of vascular events more than does a standard statin dose, with no evidence of any threshold below which further LDL-lowering is hazardous.[^2^](#_ENREF_2) For example, in the recent Cholesterol Treatment Trialists’ (CTT) meta-analysis, there was no excess of non-vascular death or of cancer among patients in whom LDL cholesterol was lowered from about 1.5 to 1.1 mmol/L (58 to 43 mg/dL), while the proportional reduction in major vascular events was consistent with the effect seen among those with higher baseline LDL cholesterol levels.[^31^](#_ENREF_31) In the present trial, all individuals will receive an atorvastatin regimen intended to reduce their LDL cholesterol below 2 mmol/L (77 mg/dL). For individuals recruited in North East Asia (e.g. China or Japan), an atorvastatin dose of either 10 mg or 20 mg daily will be used depending on the individual’s screening total cholesterol level and current LDL-lowering treatment. For those in all other countries, a similar algorithm will be employed to determine the atorvastatin dose of 20 mg or 80 mg daily. In all countries, total cholesterol will be checked after a minimum of 8 weeks of pre-randomization atorvastatin treatment. Individuals will be excluded (prior to randomization) if their total cholesterol is above 4 mmol/L (155 mg/dL), which is approximately equivalent to LDL cholesterol above 2.5 mmol/L (97 mg/dL). Estimates based on the lipid profiles of similar patient populations in the HPS2-THRIVE study indicate that, after 8 weeks of these atorvastatin regimens, fewer than 10% of screened individuals would have LDL cholesterol above 2.5 mmol/L (97 mg/dL) and would be excluded from this study. Among eligible individuals subsequently randomized to placebo in the present trial, the mean LDL cholesterol should be about 1.7 mmol/L (67 mg/dL). Allocation to anacetrapib 100 mg daily should lower LDL cholesterol by about a further 40% to around 1.0 mmol/L (40 mg/dL), but only about 1% of those participants would be expected to achieve LDL cholesterol levels below 0.5 mmol/L (19 mg/dL), even if they are fully compliant with all of the study treatments (Figure 2).

***Figure 2.*** *Anticipated LDL cholesterol concentrations among randomized trial participants*

*(Individuals with LDL cholesterol above 2.5 mmol/L on the study atorvastatin regimens would be excluded. Full compliance with all of the study treatments has been assumed)*

## Brief Summary of New Information Since Protocol Version 1

The following new information has emerged since the background and rationale for the trial were originally written for protocol version 1.0:

- The results of HPS2-THRIVE have been published: Among participants with atherosclerotic vascular disease, the addition of extended-release niacin-laropiprant to statin-based LDL cholesterol-lowering therapy did not significantly reduce the risk of major vascular events, but did increase the risk of serious adverse events related to gastrointestinal and musculoskeletal systems, skin, diabetes, infection and bleeding.[^32^](#_ENREF_32)
- The Dal-OUTCOMES trial was stopped prematurely in May 2012 due to a lack of clinically meaningful efficacy: Dalcetrapib increased HDL-cholesterol by about 30% (with no effect on LDL-cholesterol) but did not alter the risk of the primary outcome (hazard ratio 1.04; 95% confidence interval, 0.93 to 1.16; P = 0.52) or have a significant effect on any component of the primary outcome. There were no significant safety concerns.[^33^](#_ENREF_33)
- The DEFINE study showed that, after treatment with anacetrapib, the Friedewald formula and two direct methods (Roche and Genzyme) for assessing LDL cholesterol gave lower values than those measured by a Beta Quantification (BQ) method.[^34^](#_ENREF_34) Hence the LDL-lowering effect of anacetrapib may be of the order of 20-30% rather than the 40% previously reported.
- Extended follow-up of a subset of DEFINE participants showed that anacetrapib has a long terminal half-life. Low levels of anacetrapib (7-8% of apparent steady-state on-treatment trough exposures, and 1-2% of apparent steady state peak concentrations) were detected in the blood 2.5-4 years after cessation of therapy.[^35^](#_ENREF_35)
- The results of the IMPROVE-IT trial were announced in November 2014 and subsequently published in June 2015.[^36^](#_ENREF_36) Among 18,000 patients with acute coronary syndrome, the addition of ezetimibe 10mg to simvastatin 40 mg daily reduced the risk of the composite primary outcome of cardiovascular death, myocardial infarction, hospital admission for unstable angina, coronary revascularization (≥30 days after randomization) or stroke (HR 0.94, 95% CI 0.89-0.99; p=0.016). Among the tertiary analyses, there was a significant reduction in ischemic stroke (HR 0.79; 95% CI 0.67-0.94; p=0.008) but not in coronary revascularization (HR 0.96; 95% CI 0.90-1.02).
- The ACCELERATE trial, a 12000 person phase III cardiovascular outcome trial of evacetrapib versus placebo, was stopped early due to insufficient efficacy in October 2015. There is reported to be no safety signal.[^37^](#_ENREF_37)^,^ [^38^](#_ENREF_38) Full presentation of the study results is awaited.

Each of these new pieces of information has been considered by the Trial Steering Committee as they became available. Blind to analyses of the study results by treatment allocation (section 2.4.4), the Trial Steering Committee has agreed to a number of changes to the secondary assessments as described in this version 2.0 of the protocol.

1. PLAN OF INVESTIGATION

## Study Aims

The study will randomize at least 30,000 participants aged 50 years or older with pre-existing atherosclerotic vascular disease between anacetrapib 100 mg daily and matching placebo for a median of about 4 years. The primary aim is to assess the effect of anacetrapib on the composite outcome of Major Coronary Event (MCE), defined as coronary death, myocardial infarction or coronary revascularization (see Section 2.3.1.1). The key secondary aim is to assess the effect of anacetrapib on coronary death, myocardial infarction or presumed ischaemic stroke (see Section 2.3.1.2). Other secondary, tertiary and exploratory assessments (including analyses of safety and biochemical efficacy) are described in Sections 2.3.1.2 to 2.3.1.7)

## Treatment Comparisons

### Run-in period prior to randomization

At the initial Screening visit, eligible individuals (see Section 3.1) will be issued with a 12-week supply of Run-in medication consisting of:

- placebo anacetrapib; and
- active atorvastatin (see Section 3.2.2 for dose).

One tablet of each treatment is to be taken daily with a meal for 8-12 weeks (see Figure 3).

***Figure 3.*** *Outline of randomization and follow-up schedule*

### Randomization to anacetrapib versus placebo

Eligible and consenting individuals will be allocated anacetrapib or placebo using a minimized randomization program that helps maximize balance between the treatment groups with respect to prognostically important variables (including age, gender, history of prior disease, smoking status, dry chemistry total cholesterol, blood pressure, ethnic origin, prior statin use, and study LDL-lowering regimen).[^39^](#_ENREF_39) The algorithm includes a stochastic element (treatment is assigned to the arm determined by the minimization algorithm with a probability of 0.9).

Randomized participants will be issued with a supply of study treatment consisting of:

- anacetrapib 100 mg or matching placebo; and
- active atorvastatin (at the same dose issued at the Screening visit).

One tablet of each treatment is to be taken daily with a meal.

## Data Analysis Plan

### Main and subsidiary analyses

#### Primary assessment

Primary assessment will involve an intention-to-treat comparison among all randomized participants of the effects of allocation to anacetrapib versus placebo on major coronary events (defined as the occurrence of coronary death, myocardial infarction or coronary revascularization procedure) during the scheduled treatment period.

#### Secondary assessments

Secondary assessments will involve intention-to-treat comparisons among all randomized participants of the effects of allocation to anacetrapib versus placebo during the scheduled treatment period on:

1. Major atherosclerotic events (defined as coronary death, myocardial infarction or presumed ischaemic stroke; the key secondary outcome);
2. Presumed ischaemic stroke (i.e. not known to be haemorrhagic); and
3. Major vascular events (defined as coronary death, myocardial infarction, coronary revascularization or presumed ischaemic stroke).

In addition, each of the individual components of the primary outcome (i.e. coronary death; myocardial infarction; and coronary revascularization) will be tested separately.

#### Additional assessments requested by regulatory agencies

These assessments (which have been specifically requested prior to the start of the study by regulatory agencies) will involve intention-to-treat comparisons among all randomized participants of the effects of allocation to anacetrapib versus placebo during the scheduled treatment period on:

1. Cardiovascular death or myocardial infarction; and
2. Cardiovascular death, myocardial infarction or stroke.

#### Tertiary assessments

Tertiary assessments will involve intention-to-treat analyses among all randomized participants of the effects of allocation to anacetrapib versus placebo during the scheduled treatment period on:

1. Coronary death or myocardial infarction, and, separately myocardial infarction alone;
2. Mortality from all causes combined and, separately, within particular categories of causes, including cardiovascular (e.g. coronary [including sudden cardiac death]; other cardiac; stroke; other vascular) and non-vascular (e.g. cancer; infection, respiratory, hepatic; other medical, and non-medical) causes;
3. Confirmed ischaemic stroke, confirmed haemorrhagic stroke, and stroke of unknown/unconfirmed aetiology, considered separately and combined;
4. Major coronary events, major atherosclerotic events, and major vascular events in various subdivisions:
   1. occurring more than one year after randomization;
   2. prior disease type (i.e. coronary heart disease; cerebrovascular disease; peripheral arterial disease; diabetes) and timing (i.e. most recent qualifying vascular event within 12 months of randomization);
   3. three similar-sized groups based on lipid and apolipoprotein measurements (including HDL and LDL cholesterol) from the Randomization visit;
   4. various categories of participant (e.g. according to age, sex, region, blood pressure, kidney function, alcohol intake, cigarette smoking, body mass index, waist:hip ratio, history of heart failure, atorvastatin dose) based on their Randomization visit values;
   5. presence and absence of other treatments used at the Randomization visit (e.g. angiotensin-converting-enzyme inhibitors or angiotensin-receptor blockers, aspirin or other antiplatelet drugs, diuretics, calcium-channel blockers, beta-blockers);
5. Urgent and non-urgent coronary revascularization, considered separately and combined;
6. Non-coronary revascularizations, including percutaneous interventions (with or without stenting), surgical revascularization procedures (e.g. grafting, endarterectomy), and amputation for presumed vascular disease;
7. Hospitalization for heart failure;
8. Development of diabetes mellitus;
9. Combination of first and subsequent occurrences of the primary outcome;
10. Cancer at all sites (fatal or non-fatal), and site-specific cancers considered separately (excluding any known to pre-date randomization and non-melanoma skin cancers); and
11. Serious adverse events (overall and by subtype) due to infection and, separately, due to respiratory disease.

#### Additional safety assessments

Additional safety assessments will include intention-to-treat analyses among all randomized participants of the effects of allocation to anacetrapib versus placebo during the scheduled treatment period on:

- systolic and diastolic blood pressure
- muscle-related outcomes:
  - creatine kinase [CK] >5x and ≤10x laboratory upper limit of normal [ULN] plus ALT >1.5x ULN
  - CK >10x and ≤40x ULN overall and, separately, with muscle symptoms
  - CK >40x ULN overall and, separately, with evidence of end-organ damage such as doubling of serum creatinine (i.e. rhabdomyolysis)
- liver-related outcomes:
  - ALT >3x ULN plus bilirubin >2x ULN, with CK ≤5x ULN
  - ALT >3x ULN on 2 occasions within about one week, with CK ≤5x ULN
  - liver injury of unknown cause
- renal function
  - changes in estimated glomerular filtration rate
  - development of microalbuminuria and macroalbuminuria
- discontinuation of study treatment overall and by various causes

#### Analyses of biochemical efficacy

Biochemical efficacy of anacetrapib 100 mg daily will be assessed in non-fasting specimens taken from all participants at the randomization visit, at the 2 month follow-up visit, at a follow-up visit when median follow-up is about 2 years, and at the final study visit. In addition, samples will be taken annually in 5% of participants. The following biochemical efficacy outcomes will be measured on all samples:

- total cholesterol
- HDL cholesterol
- LDL cholesterol
- non-HDL cholesterol
- triglycerides
- apolipoprotein A1
- apolipoprotein B

In addition, lipoprotein (a) will be measured in all participants at randomization and at about 2 years after the median participant is randomized, and in at least 5% of participants annually.

The effect of anacetrapib on other aspects of lipid and lipoprotein profile (such as lipoprotein particle size) may also be examined. Exploratory analyses will be conducted among particular categories of participant.

#### Exploratory assessments

Exploratory assessments will be made of other possible beneficial or adverse effects of anacetrapib during the scheduled treatment period and in more prolonged follow-up thereafter. In particular, wherever possible, effects on vascular events, cancer, deaths and other serious adverse events will be assessed during at least the first few years following the end of the scheduled treatment period. In interpreting the results of the many exploratory analyses that will be performed, allowance will be made for multiple hypothesis testing, their exploratory (and, perhaps, data-dependent) nature, and for evidence from other studies. Analyses of fatal events will be interpreted in the light of the observed effects on relevant non-fatal events.[^40^](#_ENREF_40)

#### Health economic assessments

The study results will be used to conduct appropriate health economic assessments regarding the use of anacetrapib among patients at risk of vascular events.

### Statistical analysis

All participants randomized to anacetrapib will be compared with all participants randomized to placebo, regardless of whether a participant received all, some or none of their allocated treatment (i.e. intention-to-treat [ITT] analyses). A participant may contribute to more than one assessment if they have events of more than one type (e.g. non-fatal ischaemic stroke followed by coronary death). For the time-to-event analyses, survival analytic methods will be used to evaluate the time to the first event during the entire study period. For each outcome, log-rank method will be used to estimate the average event rate ratio comparing all those allocated active anacetrapib with all those allocated placebo.[^40^](#_ENREF_40) Estimates of the event rate ratio will be shown with 95% confidence intervals, and Kaplan-Meier estimates for the time to each of the primary and secondary outcomes will also be plotted (with their associated log-rank p-values). In all analyses, two-sided p-values (2P) <0.05 will be considered statistically significant (after any adjustment).

The primary outcome will be assessed without adjustment for multiplicity. If a significant difference is demonstrated, then the key secondary outcome (i.e. major atherosclerotic events) and each of the components of the primary outcome (coronary death, myocardial infarction, and coronary revascularization) will be tested without adjustment. If a significant difference is demonstrated in the key secondary outcome, then presumed ischaemic stroke will be assessed. The remaining secondary outcome of major vascular events and the two additional composite outcomes requested by regulatory agencies (see Section 2.3.1.3) will be assessed without adjustment for multiplicity.

If there is directional consistency in the effect of the treatment on the primary outcome and on presumed ischaemic stroke, emphasis will be placed on the subgroup analyses for the secondary outcome of major vascular event (which incorporates both outcomes). Tests for heterogeneity of the proportional effect observed in subgroups will be used (with allowance for multiple comparisons) to determine whether the proportional effects in specific subcategories are clearly different from the overall effect.[^40^](#_ENREF_40)^,^ [^41^](#_ENREF_41) If, however, patient categories can be arranged in some meaningful order (e.g. age at randomization: <60, ≥60<70, ≥70) then assessment of any trend will be made. For subgroups based on continuous variables (e.g. blood pressure), approximate tertiles will be used, using natural breaks to define categories (e.g. systolic blood pressure <140 mmHg rather than <138.7 mmHg). Based on the observed differences in cholesterol during follow-up between all those allocated active anacetrapib and all those allocated placebo (i.e. irrespective of compliance), cholesterol-weighted analyses will be used to estimate the effects of actual compliance with anacetrapib on the primary and secondary outcomes overall and in different circumstances.[^42^](#_ENREF_42) For each of the events listed as additional safety outcomes (section 2.3.1.5), the number of randomized participants with at least 1 event will be compared using standard tests for differences in proportions. For continuous variables, such as blood pressure (section 2.3.1.5) and analyses of biochemical efficacy (section 2.3.1.6), differences in means between the randomized groups will be assessed.

## Sample size and predicted number of events

### Initial assumptions (prior to study start)

***Anticipated rate of major coronary events:*** Blinded data from the ongoing HPS2-THRIVE trial[^20^](#_ENREF_20) indicate that the rate of non-fatal MI or coronary death is about 0.9% per annum (pa) in both China and in Western Europe (Scandinavia and United Kingdom). When coronary revascularization procedures are added, event rates are approximately twice as large: 1.8% pa in China and 1.7% pa in Western Europe.

***Anticipated effects of anacetrapib 100 mg daily on lipids:*** Data from HPS2-THRIVE have been used to model the effects on lipids of adding anacetrapib 100 mg daily to the background atorvastatin therapy. Full compliance with anacetrapib 100 mg daily would be expected to increase HDL cholesterol by 1.3 mmol/L (50 mg/dL) from 1.0 mmol/L to 2.3 mmol/L (39 mg/dL to 89 mg/dL) and to decrease LDL cholesterol by 0.7 mmol/L (27 mg/dL) from 1.7 mmol/L to 1.0 mmol/L (67 mg/dL to 40 mg/dL: see Figure 2). In estimating the likely effects on clinical outcomes, it is assumed that only about three-quarters of the participants will be taking their allocated study treatment at the midpoint of study follow-up.

***Anticipated effects of anacetrapib 100 mg daily on major coronary events:*** There is good evidence from randomized trials that, for every 1 mmol/L (39 mg/dL) reduction in LDL cholesterol achieved with a statin, the risk of major coronary events is reduced by about one-quarter.[^1^](#_ENREF_1)^,^ [^31^](#_ENREF_31) Anacetrapib reduces LDL cholesterol by a different mechanism to that of statins. But, if the relationship between LDL cholesterol reduction and risk reduction is similar, the anticipated 0.5 mmol/L reduction in LDL cholesterol (assuming three-quarters compliance at the study midpoint) would be expected to translate into a 10-15% relative reduction in major coronary events. There are insufficient data from randomized trials to make reliable estimates of the effects of increasing HDL cholesterol on the risks of such events. In the ERFC meta-analysis of prospective observational studies, 0.8 mmol/L (30 mg/dL) higher HDL cholesterol was associated with about two-fifths lower risk of non-fatal MI or coronary death.[^10^](#_ENREF_10) If, as with LDL cholesterol, only about half of the effect associated epidemiologically emerges within about 4-5 years of raising HDL cholesterol, then the anticipated 1 mmol/L increase (assuming three-quarters compliance) would be expected to translate into a 20-25% relative reduction in major coronary events.

### Statistical power (prior to study start)

Based on a major coronary event rate of 1.8% pa and median follow-up of 4 years, a trial of 30,000 participants would have 88% power at 2P<0.01 to detect a 15% relative risk reduction (see Table 2). Moreover, if the relative risk reduction was larger than 15% then the study might well produce compelling results earlier than anticipated, and might provide reliable information in important subgroups and for other major outcomes. For example, with 30,000 participants and an event rate of 0.9% pa, there would be 83% power at 2P<0.01 to detect a 20% relative reduction in the key secondary outcome of coronary death or myocardial infarction. Follow-up of 4 years in such a large trial would provide reliable information on the safety of anacetrapib.

***Table 2.*** *Statistical power to detect 15% reduction in the primary outcome of major coronary events (coronary death, MI or coronary revascularization) and 20% reductions in the original secondary outcomes among 30,000 participants with median follow-up of 4 years*

| Event rate  (pa) | Proportional reduction | Placebo  15000 | Active  15000 | Power  (2P<0.01) | Power  (2P<0.05) |
| --- | --- | --- | --- | --- | --- |
| Coronary death, MI or coronary revascularization | | | | | |
| 1.80% | 15% | 1080  (7.20%) | 918  (6.12%) | 88% | 96% |
| Coronary death or MI | | |  |  |  |
| 0.90% | 20% | 540  (3.60%) | 432  (2.88%) | 83% | 94% |
| Coronary revascularization | | |  |  |  |
| 1.20% | 20% | 720  (4.80%) | 576  (3.84%) | 94% | 98% |
| Presumed ischaemic stroke | | |  |  |  |
| 1.00% | 20% | 600  (4.00%) | 480  (3.20%) | 87% | 96% |
| Cardiovascular death | |  |  |  |  |
| 0.75% | 20% | 450  (3.00%) | 360  (2.40%) | 74% | 89% |

### Revised statistical power (protocol version 2.0)

Based on an observed blinded major coronary event rate of 2.7% pa and median follow-up of 4 years, a trial of 30,449 participants (i.e. the number randomized) would have 98% power at 2P<0.01 to detect a 15% relative risk reduction (see Table 3).

***Table 3.*** *Statistical power to detect 15% reductions in the primary outcome of major coronary events (coronary death, MI or coronary revascularization) and in the revised secondary assessments among 30,449 participants with median follow-up of 4 years*

| Event rate  (pa) | Proportional  reduction | Placebo  15224 | Active  15225 | Power  (2P<0.01) | Power  (2P<0.05) |
| --- | --- | --- | --- | --- | --- |
| Coronary death, MI or coronary revascularization | | |  |  |  |
| 2.7% | 15% | 1644  (10.8%) | 1398  (9.2%) | 98% | >99% |
| Coronary death, MI or presumed ischaemic stroke | | | | | |
| 2.2% | 15% | 1340  (8.8%) | 1139  (7.5%) | 95% | 99% |
| Presumed ischaemic stroke | | | | | |
| 0.8% | 15% | 487  (3.2%) | 414  (2.7%) | 46% | 70% |
| Coronary death, MI, presumed ischaemic stroke or coronary revascularization | | | | | |
| 3.4% | 15% | 2070  (13.6%) | 1760  (11.6%) | >99% | >99% |

### Planned study duration

Follow-up of all randomized participants is planned to continue for a median of at least 4 years, and until at least 1900 participants have had an unrefuted major coronary event (primary endpoint) plus at least 950 participants have had a coronary death or myocardial infarction. During the study, an independent Lipid Monitoring Committee will review unblinded tabulations of the achieved blood lipids (but not results for individuals) and will inform the Steering Committee if, in their view, the achieved lipid differences vary materially from those anticipated in the protocol (see section 2.4.1). The Steering Committee may use this information, together with blinded tabulations of the overall event rates (i.e. not split by allocated anacetrapib or placebo treatment), to determine whether modifications to the protocol (such as the numbers of participants recruited or the duration of follow-up) are required for the reliable assessment of the effects of anacetrapib. Wherever possible and subject to funding, extended follow-up of all surviving randomized participants will continue for several years beyond the final study visit in order to provide valuable information on the longer-term safety and efficacy of the study treatment (see section 3.4.5.).

## Data and Safety Monitoring

### Recording and reporting of adverse events

#### Definition of Serious Adverse Events (SAE)

Serious adverse events are defined as those adverse events that:

- result in death;
- are life-threatening;
- require in-patient hospitalization or prolongation of existing hospitalization;
- result in persistent or significant disability or incapacity;
- result in congenital anomaly or birth defect;
- are cancer; or
- are important medical events in the opinion of the responsible investigator (i.e. not life-threatening or resulting in hospitalization, but may jeopardise the participant or require intervention to prevent one or other of the outcomes listed above);

Pregnancies and overdoses of study medication are also to be recorded as serious adverse events. Overdose is defined as any occasion in which an investigator learns that a participant has taken more than 6 times the specified dose of study medication.

#### Reporting of Serious Adverse Events and other relevant events

All SAEs reported by participants at each follow-up visit will be recorded directly on the study computer-based data entry system (see Section 2.6.3), regardless of whether the participant continues to take study treatment or not. Clinic staff are to record whether any SAE is thought likely to be due to study treatment (see Section 2.5.1.3). Participants will also be asked specifically whether they have had any unexplained muscle pain or muscle weakness to determine whether additional measures are required to detect and manage statin-related myopathy (see Sections 3.4.2 and 3.5.1.2). Other adverse events not considered serious (as defined above) will only be recorded if they lead to discontinuation of study treatment or are believed to be due to study treatment. SAEs that are potential primary or secondary study endpoints, or important safety endpoints will undergo central review, verification and coding as described in Section 3.6. In addition, blood pressure and blood tests for assessment of safety are to be recorded at each visit (see Section 2.6.4.1). Line-listings of all reported SAEs (blind to treatment allocation) will be provided on a bi-weekly basis to Merck Sharp & Dohme Corp. (from here on referred to as "Merck") for regulatory purposes.

#### Reporting of Suspected Serious Adverse Reactions (SSARs)

As recommended by regulatory authorities,[^43^](#_ENREF_43) a Suspected Serious Adverse Reaction (SSAR) should be considered an unanticipated problem requiring expedited reporting only if it is either an event that is uncommon and strongly associated with drug exposure (such as angioedema, agranulocytosis, hepatic injury, or Stevens-Johnson syndrome), or an event that is not commonly associated with drug exposure, but uncommon in the study population (e.g. tendon rupture, progressive multifocal leukoencephalopathy).

Any SAE that is considered, with a reasonable probability, to be due to study treatment is, potentially, a SSAR. In making this assessment, there should be consideration, based on the available information, of the probability of an alternative cause, the timing of the reaction with respect to study treatment, the response to withdrawal of the study treatment, and (where appropriate) the response to subsequent re-challenge. Reports of all such events are to be forwarded immediately to a clinician at the Regional Coordinating Centre (RCC) or Central Coordinating Office (CCO). This clinician will obtain standard information, including participant study number, identity of reporting person, description of event, and reason for attribution to study treatment. All such reports will then be forwarded urgently to the Clinical Coordinators (or their delegated deputies) at the CCO, who will review the evidence for seriousness and relatedness, seek any additional information required (including relevant information relating to medical history and treatment both prior to and following randomization), and assess expectedness. Each such SSAR will generally be classified as “expected” and not subject to expedited reporting if the Clinical Coordinators (or their deputies) consider it either (i) to be a study endpoint (listed in sections 2.3.1.1 to 2.3.1.4),[^43^](#_ENREF_43)^,^ [^44^](#_ENREF_44) or (ii) to have been reported previously as being associated with either anacetrapib (based on the latest version of the Investigator’s brochure and emerging data from this and other ongoing studies) or atorvastatin (based on the latest version of the clinical data sheet). Any SSARs that are considered to be “unexpected” (Suspected Unexpected Serious Adverse Reactions [SUSARS]) and are believed to be related to randomized treatment (rather than atorvastatin) will be unblinded. All SUSARs that are believed to be related to atorvastatin or to active anacetrapib will be reported immediately to Merck for regulatory authority submission, to the Chairman of the Data Monitoring Committee and, as required, to other relevant parties (including appropriate ethics committees and Institutional Review Boards [IRBs]). In addition, all expected SSARs will be reported in an expedited fashion, blinded to Merck and unblinded to the Chairman of the Data Monitoring Committee.

### Interim analyses: role of the Data Monitoring Committee

#### Frequency of reviews

During the study, interim analyses of all SAEs and other study outcomes (both overall and in key subgroups, including by region and by baseline lipid levels) will be supplied in strict confidence to the independent Data Monitoring Committee (DMC). The DMC will request such analyses at a frequency relevant to the stage of the study (typically at 6-12 monthly intervals, with a Chairman’s review every 3-6 months) or in response to emerging data from other trials. Unless advised by the DMC in response to clear evidence of benefit or hazard, the Steering Committee, collaborators, participants, representatives of Merck, and all study staff (except those who provide the confidential analyses to the DMC) will remain blind to the interim results on mortality and major morbidity until the end of the study.

#### Early stopping for benefit

In the light of these interim analyses and any other information considered relevant, the DMC will advise the Steering Committee if, in their view, the randomized comparisons in the study have provided **both** (i) “proof beyond reasonable doubt” that prolonged use of anacetrapib reduces the primary outcome (major coronary events), plus the key secondary outcome (coronary death or myocardial infarction), plus cardiovascular mortality, with a consistent effect on all-cause mortality; **and** (ii) evidence that might reasonably be expected to influence materially patient management by many clinicians who are already aware of the results of other relevant trials. Any recommendation to stop the trial early for benefit should be based primarily on adjudicated events, with a sensitivity analysis showing a similar trend when all events (adjudicated and non-adjudicated) are included. In general, at least 75% of relevant events should have been adjudicated prior to any review that might lead to early stopping for benefit. Appropriate criteria of proof beyond reasonable doubt cannot be specified precisely, but in general to justify halting, or modifying, the study prematurely for benefit then it is expected that the guidelines set out in Table 4 would be fulfilled. This approach, which will be applied at each review of the emerging data, has the practical advantage that the exact number of interim analyses is of little importance.[^40^](#_ENREF_40) For example, if allowance was made for interim assessments of the primary outcome only, then it can be shown that adjustment of a two-sided P value of 0.05 might yield a value of 0.049. But, since early stopping also requires consistency of the effect on the primary outcome across key subgroups plus highly significant effects on other vascular outcomes plus directional consistency for total mortality, the required adjustment would be even smaller. Consequently, a final two-sided P value of 0.05 will be considered significant.

***Table 4.*** *Guidelines for early stopping for efficacy*

| **Median follow-up** | **Outcome** | **Stopping criteria** |
| --- | --- | --- |
| **≥3.0 years** | Primary outcome | ≥3 SD (with consistent results in key subgroups) |
|  | Coronary death or MI | ≥2.6 SD overall plus ≥2.0 SD for at least one component |
|  | Cardiovascular mortality | ≥2.0 SD |
|  | Total mortality | Directionally consistent effect |

SD = standard deviations

#### Early stopping for safety

The DMC would also be expected to advise the Steering Committee if clear evidence emerged of an adverse effect on all-cause mortality or on cardiovascular mortality (at least 2 standard deviations) or if, in the view of the DMC, there was other compelling evidence of hazard that seemed likely to outweigh any potential benefit.

## Central and Regional Coordination of Local Clinical Centres

The Study will be coordinated by the Central Coordinating Office (CCO), based at the Clinical Trial Service Unit (CTSU) of Oxford University, working with Regional Coordinating Centres (RCCs) which will be responsible for selection of Local Clinical Centres (LCCs) within their region and for the administrative support and monitoring of those LCCs. At each LCC, a lead investigator and research coordinator (usually a qualified nurse, but in some cases may be medically qualified or have other relevant qualifications and experience) will be responsible for identification, recruitment, and follow-up (see Appendix 1: Organisational Structure and Responsibilities). It is intended that at least 30,000 participants will be randomized at about 400 LCCs worldwide.

### Training and monitoring

The study will be conducted in accordance with the principles of International Conference on Harmonisation Guidelines for Good Clinical Research Practice (ICH-GCP) and relevant local, national and international regulations (including the EU Clinical Trial Directive and the US Code of Federal Regulations Chapter 21). Prior to initiation of the study at any LCC, the RCC will confirm that the LCC has adequate facilities and resources to carry out the study (and, if considered necessary, a site visit will be undertaken). LCC lead investigators and study clinic staff will be provided with materials detailing relevant study procedures for LCCs, and clinic staff will be trained in study methods (including use of the bespoke computer-based study management systems; see Section 2.6.3).

During the study, the relevant RCC and/or the CCO will arrange monitoring visits to study centres as considered appropriate based on perceived training needs and the results of central statistical monitoring of study data. The purpose of such visits will be to ensure that the study is conducted according to the protocol, particularly by helping LCC staff to resolve any local problems with the study and by providing additional, focussed training. Particular attention will be given to the effectiveness of strategies to recruit appropriate participants, the completeness of follow-up, the maintenance of participant compliance with the study treatments, and the reporting of study outcomes and collection of relevant supporting documentation. A report of each visit will be prepared by the study monitor and provided to LCC, RCC and CCO staff.

### Supply of study materials

Study treatments will be manufactured, packaged, labelled and delivered to each LCC or RCC by Merck under the direction of the CCO. An inventory of study drug supplies will be maintained on the study computer system and monitored at the CCO. LCC lead investigators will be responsible for making appropriate arrangements for the storage and issuing of study treatments, and for the disposal of unused study drug in accordance with study Standard Operating Procedures (SOPs).

### Data management

All data in the study will be processed electronically using a set of custom-written applications. The LCC clinic staff will use a bespoke laptop-based application for performing LCC tasks (including: entering participant data at study visits; minimized randomization and issue of appropriate study treatment; and local trial administration), with frequent synchronization of data to central databases. RCC and CCO staff will use a suite of administration applications to manage centres and study participants, including clinical supervision (review of SAEs and safety blood results), management of follow-up and compliance, tracking of samples for central analysis, collection of supporting documentation for relevant events, and endpoint adjudication. Data transfers will be cryptographically secured, and all data will be stored securely. All data accesses will require a unique username and password, and any changes to data will require the user to enter their username and password as an electronic signature. Staff will have access restricted to only the functionality and data that are appropriate to their role in the study.

### Biological sample assay, transport and storage

#### Local analysis of eligibility and safety bloods

Dry chemistry analysers will be used in all LCC study clinics for eligibility checks at the Screening visit (ALT, CK and creatinine) and Randomization visit (ALT and CK); for baseline measures of total cholesterol (at Screening and Randomization Visits); and for safety analyses during follow-up (ALT at each visit, with other liver function tests and CK as required).

#### Central assessment of samples collected at the randomization visit and during follow-up

Samples of blood and urine are to be collected from all randomized participants at the Randomization visit for central analysis and storage, including subsequent DNA extraction (subject to relevant consent, see Section 2.6.4.3). Further samples are to be collected from all participants at the 2 month visit, at around 2 years after the median participant is randomized and at the final study visit. In addition, samples of blood are to be collected annually from 5% of participants (starting 1 year after the first participant is randomized) (see Appendix 2: Visit schedule and procedures). RCCs will supply LCC clinic staff with vacuum tubes to collect these blood samples, which are to be kept cool before centrifugation, separation into bar-coded cryovials, and storage at below -18°C within a day of the clinic visit. Samples are to be transferred to below -40°C within 4 weeks. At appropriate intervals, all samples will be collected from the LCCs (by the RCC or CCO) and transferred to a central laboratory for analysis (see section 5.2.2), and for long-term storage in liquid nitrogen vapour or in electric freezers (remnant blood for DNA extraction only).

#### Consent approval for unspecified analyses on blood and urine samples

Sample tubes will be labelled with a unique Sample ID which will be linked to the participant and the study visit using the study data entry system (i.e. samples will be pseudonymised). Outside the study clinic, staff involved in the transport, storage and analysis of these samples will have no means of linking tubes to an identifiable participant. Consent for protocol-specified analyses (including assays of lipid and lipoprotein profile) will be included in the main consent form. In addition, all participants will be asked if they would provide Supplementary Consent to allow samples of plasma, serum and urine that have been collected to be retained and used for unspecified analyses in the future. Similarly, Supplementary Consent will be sought to permit genetic material in the blood samples to be analysed. In all cases, participants will be free to opt in or out of any part of the Supplementary Consent without affecting their eligibility for the trial.

### Administrative details

#### Source documents and archiving

Source documents for the study constitute the clinic visit records (including blood and urine assay results) held in the study main data store, the additional information obtained on reported serious adverse events that are relevant to the outcome measures (see Section 3.6), death certificates, and drug supply records. These will be retained for at least 15 years from the completion of the study. Merck and regulatory agencies will have the right to commission a confidential audit of such records in the CCO, RCCs, and LCCs provided this does not result in unblinding while the study is in progress.

#### Sponsor and funding

This study has been designed by the independent investigators at the Clinical Trial Service Unit (CTSU) at Oxford University in collaboration with the TIMI Study Group based at Brigham Women’s Hospital, Harvard Medical School, Boston, and with Merck. The University of Oxford will act as the trial Sponsor worldwide. The data will be collected, analysed and published independently of the source of funding. Merck is providing funding for the design and overall conduct of the study.

#### Indemnity

Merck will, at all times, indemnify the study investigators and study staff from claims that may be made against them for any injury sustained by a study participant as a consequence of participation in the study in accordance with this protocol. The indemnity will be outlined in detail in the agreements between the CCO, RCCs and LCCs (and, if required, in a letter from Merck).

#### End of trial

The end of the main phase of the trial is defined as the date of the last visit of the last participant. It is planned that wherever possible, and subject to funding, follow-up of all participants will continue for at least 2 years after the date of the last visit of the last participant (see Section 3.4.5).

#### Publications, reports and substudies

The independent Steering Committee will be responsible for drafting the main reports from the study and for review of any other reports. Draft copies of any manuscripts will be provided to lead investigators at each LCC, and all other collaborators (including Merck) for review prior to publication. Papers will be written in the name of the Collaborative Group, with individual investigators named personally at the end of the report (or, to comply with journal requirements, in web-based material posted with the report).

Proposals for substudies on participants randomized into the study must be approved by the Steering Committee before they begin. In considering such proposals, the Steering Committee will need to be satisfied that the proposed substudy is of a high quality, and that it will not compromise the main study in any way (for example by reducing the recruitment rate or compliance with study treatment).

1. SUMMARY OF PRACTICAL PROCEDURES

| **PRE-SCREENING PHASE** | |
| --- | --- |
|  | - Identify potentially eligible individuals (age ≥50 years with vascular disease) - Invite to attend Screening clinic appointment in local study clinic |
| **SCREENING VISIT (-2 to 3 MONTHS) AND PRE-RANDOMIZATION RUN-IN PHASE** | |
|  | - Medical history, relevant current treatment and other eligibility factors recorded - Blood pressure recorded - Written informed consent sought from eligible and willing individuals - Blood sample taken for dry chemistry assays (total cholesterol, ALT, CK, and creatinine) - Consenting participants asked to stop non-study statin or ezetimibe - Active atorvastatin plus placebo anacetrapib issued - Randomization visit appointment scheduled for about 10 weeks later - Individual’s doctor (and/or investigator) informed about entry into run-in phase |
| **RANDOMIZATION VISIT (0 MONTHS)** | |
|  | - All serious adverse events during Run-in and non-study treatments recorded - Final check of compliance, eligibility and consent - Assessment of quality of life, including EQ5D questionnaire - Blood pressure, height, weight, and hip, waist and neck circumference recorded - Blood sample taken for dry chemistry assays (ALT, CK, and total cholesterol) and for central analyses and frozen storage - Urine collected for albumin:creatinine ratio and storage - Randomization via study clinic computer: allocated anacetrapib 100 mg daily or placebo - Treatment pack issued: atorvastatin plus randomized treatment - First follow-up visit appointment scheduled for 2 months’ time - Participant’s doctor informed of participant’s randomization |
| **FOLLOW-UP VISITS AT 2 and 6 MONTHS, THEN 6-MONTHLY** | |
|  | - Serious adverse events, compliance, and changes to non-study medication recorded - Non-serious adverse events attributed to study treatment recorded - Reasons for stopping study treatments recorded - Blood pressure recorded - Blood sample taken for dry chemistry safety assays, and for central analysis and storage for future assays (in all participants at the 2 month visit, at 2 years after median participant randomized, and at the final visit, and in 5% of participants annually) - Urine collected for albumin:creatinine ratio and storage (final visit only) - Follow-up treatment pack issued: atorvastatin plus randomized treatment - Next Follow-up visit scheduled for 6 months and then 6 monthly - Assessment of cognitive function and quality of life (including EQ5D questionnaire) at final visit only |
| **MONITORING OF SAFETY AND EFFICACY** | |
|  | - Central monitoring of blood results and adverse events (with Early Recall visits to monitor any problems) - Further details on relevant outcomes sought from participant’s doctor and other sources (e.g. registries and electronic healthcare records) - Relevant events confirmed centrally blind to treatment allocation |

## Eligibility For the Study

Patients are eligible for randomization if:

1. at least one of the inclusion criteria is satisfied; and
2. none of the exclusion criteria applies; and
3. their own doctor does not consider there to be
   1. a definite indication for a CETP inhibitor^[[1]](#footnote-1)^; or
   2. a definite contraindication to either anacetrapib or atorvastatin

### Inclusion criteria

Patients must be aged at least 50 at the time of initial invitation, and at least one of the following inclusion criteria must be satisfied:

- History of MI; or
- Cerebrovascular atherosclerotic disease (i.e. history of presumed ischaemic stroke or carotid revascularization); or
- Peripheral arterial disease (i.e. history of non-coronary revascularization, including aortic aneurysm repair or graft); or
- Diabetes mellitus with other evidence of symptomatic coronary heart disease (i.e. treatment or hospitalization for angina, or a history of coronary revascularization or acute coronary syndrome).

### Exclusion criteria

None of the following must be satisfied:

- Acute MI, acute coronary syndrome or stroke within 4 weeks prior to Screening Visit or during Run-in (but such individuals may be entered later, if appropriate);
- Planned coronary revascularization procedure within the next 6 months (such individuals may be entered later, if appropriate);
- Definite history of chronic liver disease, or abnormal liver function (i.e. ALT >2x ULN). Note: Individuals with a history of acute hepatitis are eligible provided this ALT limit is not exceeded;
- Severe renal insufficiency (i.e. creatinine >200 µmol/L [2.3 mg/dL], dialysis or functioning renal transplant);
- Evidence of active inflammatory muscle disease (e.g. dermatomyositis, polymyositis), or CK >3x ULN;
- Previous significant adverse reaction to a statin or anacetrapib;
- Current treatment with any of the following lipid-lowering treatments:

1. a regimen considered to produce substantially greater LDL cholesterol reduction than atorvastatin 80 mg daily for individuals in non-Asian countries or 20 mg daily for those in North East Asia; or
2. fibric acid derivative (“fibrate”, including gemfibrozil); or
3. niacin (nicotinic acid) at doses above 100 mg daily

- Concurrent treatment with a medication that is contraindicated with anacetrapib or atorvastatin:

1. any potent CYP3A4 inhibitor, such as:
2. macrolide antibiotics (erythromycin, clarithromycin, telithromycin);
3. systemic imidazole or triazole antifungals (e.g. itraconazole, posaconazole);
4. protease inhibitors (e.g. atazanavir);
5. nefazodone
6. ciclosporin
7. daptomycin
8. systemic use of fusidic acid

Note: Individuals who are taking such drugs temporarily may be re-screened when they discontinue them, if considered appropriate;

- Known to be poorly compliant with clinic visits or prescribed medication;
- Medical history that might limit the individual’s ability to take trial treatments for the duration of the study (e.g. severe respiratory disease; history of cancer or evidence of spread within last 5 years, other than non-melanoma skin cancer; or recent history of alcohol or substance misuse);
- Women of child-bearing potential (unless using adequate contraception);
- Current participation in a clinical trial with an unlicensed drug or device.

Individuals will also be excluded at the Screening visit if it is considered unlikely that they will achieve total cholesterol <3.5 mmol/L (135 mg/dL) on the highest atorvastatin dose available in their region (atorvastatin 80 mg daily in non-Asian countries or 20 mg daily in North East Asia).

In addition, individuals will be excluded at the Randomization visit if any of the following are true:

- Total cholesterol above 4 mmol/L [155 mg/dL]
- Non-compliant with run-in treatment (<90% scheduled run-in medication taken)
- Individual is no longer willing to be randomized into the 4-5 year trial
- The individual’s doctor is of the view that their patient should not be randomized (see Section 3.2.3)

## Screening Visit and Pre-randomization Run-in

### Assessment of relevant medical history and eligibility

Potentially eligible individuals will be given information about the study and invited to attend a Screening Visit. At that visit, relevant medical history and other factors pertinent to eligibility will be recorded directly onto the Screening Visit Form on the study IT system. The LCC clinic staff will check inclusion and exclusion criteria (with the assistance of the study IT system), record relevant current medication and measure blood pressure.

### Invitation to participate and standardization of LDL-lowering therapy

Individuals who appear to be eligible will have the study explained to them by the clinic staff, using the Participant Information Leaflet and Consent Form as a basis for discussion. Each individual will have an opportunity to initiate discussion, and have time to think about their participation in the study, perhaps after discussing it with their family, primary care physician, or a local physician. (Eligible individuals who choose to do this will be asked to attend a repeat Screening visit within a few weeks.) Attendees will be discouraged from participating if it is thought unlikely that they would be willing and able to continue attending Follow-up visits for at least 4-5 years.

Potentially eligible individuals who agree to take part will be asked to provide written informed consent to take part in the study. In addition, individuals will be asked to indicate on the consent form which, if any, of the samples of plasma, serum, cells or urine may be stored long-term for future unspecified research analyses. A blood sample will be taken for immediate dry chemistry analysis of total cholesterol, ALT, CK and creatinine. These results will be recorded onto the study clinic IT system, which will provide an assessment of eligibility (see Section 3.1) and guidance on the atorvastatin dose to be used for that particular individual. In North East Asia (e.g. China or Japan), atorvastatin will be used at a dose of either 10 mg or 20 mg daily, while elsewhere, the dose will be 20 mg or 80 mg daily. All participants are to receive a dose that is at least as effective as their current LDL-lowering treatment. In all countries, a higher dose is to be used in those individuals who – based on their screening total cholesterol and pre-trial lipid-lowering therapy – do not appear likely to achieve total cholesterol <3.5 mmol/L (135 mg/dL) on the lower atorvastatin doses. The LCC clinic staff will issue 12 weeks’ supply of the appropriate study medication, as follows:

- Active atorvastatin one tablet daily (dose determined as above)
- Placebo anacetrapib one tablet daily

Other lipid-modifying treatment (including statin, ezetimibe or resins) will be stopped. For individuals on oral anticoagulants, the managing doctor will be advised to check their international normalised ratio (INR) about one week after starting the Run-in treatment. An appointment will be made for the Randomization visit in 8-12 weeks.

### Review of eligibility and LDL cholesterol control by each individual’s own doctor

The pre-randomization Run-in phase is intended to help identify and exclude before randomization those individuals who would be unlikely to comply with long-term study treatment and follow-up. During run-in, each individual’s responsible doctor (e.g. primary care physician or hospital specialist) will be given a description of their patient’s medical history, previous lipid-modifying treatment and Screening Visit total cholesterol result, and advised of the atorvastatin dose that would be provided during the trial. The doctor would be asked to indicate whether, in their view, these blood results (or any other factor) make their patient unsuitable for entry into the randomized phase of the study.

## Randomization Visit (0 months)

### Final check of eligibility and compliance before randomization

Individuals who attend their Randomization Clinic appointment will be asked if they have experienced MI, arterial revascularization (coronary or non-coronary), stroke, other SAE or any other significant problems during the Run-in period, and whether any arterial revascularization procedure is currently planned for them. Any SAE considered to be due to study treatment (i.e. possible SAR) is to be discussed immediately with a RCC/CCO clinician for expedited reporting (see Section 2.5.1.3). Details of all non-study treatments will be sought, compliance with Run-in treatment checked (at least 90% of scheduled study treatment should have been taken), and consent information checked. Information about smoking history and alcohol intake will be sought and an assessment of quality of life is to be made (including the EQ5D questionnaire). Blood pressure, height, weight, and hip, waist and neck circumference will be measured. Details will be recorded directly onto the Randomization Form on the clinic IT system (which is designed to obtain complete information and to prompt appropriate actions).

### Collection of blood and urine samples

Eligible individuals will be asked if they are still willing to take study treatment for at least 4-5 years. If so, they will have a blood sample taken for immediate dry chemistry measurement of total cholesterol, ALT and CK in the clinic. In order to remain eligible, total cholesterol must be ≤4 mmol/L (155 mg/dL), ALT must be ≤2x ULN, and CK must be ≤3x ULN. (If an individual is excluded because of abnormal ALT or CK, their usual doctor will be informed so that further investigation can be arranged.) Blood and urine samples will be processed in preparation for subsequent transportation to the central laboratory (see Section 2.6.4).

### Random allocation of study treatment

Eligible and consenting individuals will be allocated anacetrapib or placebo using a minimized randomization program on the clinic IT system (see Section 2.2.2).[^39^](#_ENREF_39) Participants will be allocated a numbered treatment pack containing a 7-month supply of the following treatments:

- One tablet daily of active anacetrapib 100 mg or matching placebo
- One tablet daily of active atorvastatin (at a dose to match that used during run-in)

The numbered treatment packs will be issued to the participant by the LCC clinic staff or their local hospital pharmacy. An appointment for the first post-randomization Follow-up Visit will then be allocated by the study staff, with guidance from the clinic IT system. The participant’s doctor(s) will be notified that the participant has been randomized.

## Follow-up Visits (2 and 6 months and then 6-monthly)

### Recording adverse events and compliance

Following randomization, all participants are scheduled to attend Follow-up visits at 2 and 6 months, and then 6-monthly until the end of the study (see Section 2.4.3). At each visit, details of all hospital admissions, other SAEs, unexplained muscle pain or weakness, and non-serious adverse events attributed to study treatment will be sought.^[[2]](#footnote-2)^ Any SAE considered to be due to study treatment (i.e. possible SAR) is to be discussed immediately with a RCC/CCO clinician for expedited reporting (see Section 2.5.1.3). Changes to non-study treatment will be sought, and compliance with study treatment reviewed. For participants who discontinue study treatment, the reasons for doing so will be sought. Blood pressure will be measured at each visit (and, at the final visit, hip, waist and neck circumference and weight will be measured). At the final visit, cognitive function and quality of life (including EQ5D questionnaire) are to be assessed. Details are to be recorded directly onto the Follow-up Form on the clinic IT system.

### Collection of blood and urine samples

At each Follow-up visit, a non-fasting blood sample will be taken for immediate dry chemistry analysis of ALT. If ALT>2x ULN with symptoms that suggest possible hepatitis (e.g. loss of appetite, nausea, jaundice, lethargy or malaise), or if ALT>3x ULN even in the absence of such symptoms, then dry chemistry analysis of alkaline phosphatase and bilirubin are to be performed and an Early Recall visit may need to be arranged (see Section 3.5.1.1). If ALT>1.5x either ULN or the Randomization visit result, or if the participant has new or unexplained muscle symptoms (i.e. muscle pain or weakness) then CK is also to be measured. If CK>5x ULN then creatinine is to be measured. If CK>3x ULN, an Early Recall visit may need to be arranged (see Section 3.5.1.1). For all participants at the 2 month visit, at a follow-up visit about 2 years after the median participant is randomized, and at the final visit, and for 5% of participants annually, additional non-fasting blood samples are to be taken for central laboratory assays and storage (see Section 2.6.4.2). A urine sample is to be taken for central analysis and storage at the final follow-up visit only.

### Issuing study treatment and arranging further appointments

Provided continuing study treatment remains appropriate, participants will be given a further 7-month supply of their randomly allocated study treatment (anacetrapib 100 mg or placebo) and study atorvastatin, and any previously allocated treatment will be retrieved (except at the 2-month visit). An appointment will then be made for their next scheduled Follow-up visit.

### Follow-up for randomized participants not attending study clinics

Follow-up information is to be collected from all study participants, irrespective of whether they continue to take study treatment, usually at routine Follow-up clinic visits. If, however, a participant becomes unwilling or unable to attend then LCC clinic staff will telephone the participant (or, where appropriate, their relative or carer) at the time of each of their scheduled Follow-up appointments and complete the necessary Follow-up form on the clinic IT system. If this is not possible, then RCC staff will attempt to check a participant’s progress by direct correspondence with the participant’s own doctors, or by reviewing available information on routine healthcare systems and registries (see Section 3.4.5). If safety monitoring of blood is no longer possible (e.g. because the participant no longer attends clinic visits and no other means of measuring safety bloods can be arranged), then the participant will be asked to discontinue all study treatment and advised to see their own doctor so that the alternative LDL-lowering therapy can be considered.

### Follow-up of randomized participants via electronic healthcare systems and other methods during and after the scheduled follow-up period

Additional information about major clinical outcomes (including, but not limited to, those reported by participants themselves) will be sought from available electronic healthcare systems and routine data sources (for example, in the UK, NHS Connecting for Health, the Medical Research Information Service, and registries for specific diseases, devices or procedures). Such information will be used to clarify and enhance information recorded during clinic visits. Wherever possible, extended follow-up of all surviving randomized participants will continue for several years beyond the final study visit in order to provide valuable information on the longer-term safety and efficacy of the study treatment. This can be particularly informative for assessing effects on outcomes (e.g. cancers, neurodegenerative and cognitive disorders) that may only become evident many years after initiation of treatment, and for deriving appropriate cost-effectiveness analyses. Such strategies have been used in a number of previous trials, including the ALERT,[^45^](#_ENREF_45) HPS[^46^](#_ENREF_46) and WOSCOPS[^47^](#_ENREF_47) statin trials. As well as seeking long-term follow-up information via routine data sources, participant questionnaires may be administered by telephone, by mail or by electronic means (e.g. SMS text messaging or web-based survey tools).

## Early Recall Visits and Modifying Study Treatment

### Monitoring significant biochemical or other problems

An Early Recall Visit may be arranged for any participant who requires review outside their planned visit schedule. Examples of circumstances where this may be necessary include the assessment of abnormal values in safety bloods from routine Follow-up visits, and review of symptoms believed by the participant to be related to study treatment. As at routine study visits, the results of blood tests performed at Early Recall visits will be entered by LCC staff into the clinic IT system (which is designed to prompt appropriate actions) and these results will be monitored centrally by clinical staff at the CCO and RCCs in accordance with the study SOPs. For all participants randomized in Asia, an Early Recall will be conducted at 4 months to allow an additional check of biochemical safety.

#### Monitoring elevated liver transaminases

CCO clinicians will be responsible for reviewing elevated liver transaminase results, and for advising on the need for (and timing of) Early Recall visits and whether study treatment should be stopped or restarted. In so doing, CCO clinicians will collaborate with the local investigator, as necessary, and will be guided by the principles described below (but the clinical picture in some cases may necessitate a more cautious approach, e.g. more frequent study visits or a lower threshold for stopping study treatment).

If ALT>2x ULN with symptoms that suggest possible hepatitis (e.g. loss of appetite, nausea, jaundice, lethargy or malaise), or if ALT>3x ULN even in the absence of such symptoms, then treatment may continue but an Early Recall visit should be conducted in about 1 week. If ALT>2x and ≤3x ULN and there are no hepatitis symptoms, then treatment may continue and an Early Recall visit should be conducted in about 3 weeks.

At the next appointment for such individuals, ALT is to be reassessed and subsequent actions are to be guided by the following: (i) if ALT>2x ULN with symptoms that suggest possible hepatitis, or ALT>3x ULN even in the absence of such symptoms, then study treatment is to be stopped and an Early Recall visit conducted in about 6 weeks; (ii) otherwise, if ALT>2x ULN, then study treatment may continue and an Early Recall visit conducted in about 6 weeks; (iii) if ALT≤2x ULN then study treatment may continue and the participant may return to scheduled follow-up.

If at any time ALT>10x ULN or ALT>3x ULN with bilirubin>2x ULN then all study treatment (anacetrapib or placebo plus atorvastatin) is to be stopped and an Early Recall visit conducted in about 1 week.

For participants who stop study treatment with ALT>3x ULN, alternative causes of liver dysfunction should be investigated (e.g. careful history of alcohol, non-study medications, travel and diet, hepatobiliary ultrasound, and viral and autoimmune serology). Restarting study treatment should only be considered in such participants if a plausible alternative explanation has been identified. The decision to restart study treatment should be based on the clinical presentation, including the evolving pattern of liver function tests, the presence or absence of symptoms, and findings from other investigations. The CCO clinician will determine whether both study treatments (anacetrapib or placebo plus atorvastatin) are to be restarted simultaneously or sequentially. If any study treatment is restarted, there should be 2 further Early Recall visits at 4-weekly intervals. If ALT does not remain ≤2x ULN, then the study treatment is to be stopped permanently.

When study treatment is stopped, appropriate advice is to be provided to the participant’s managing doctor. If a participant develops a raised ALT after stopping all study treatment, they would not need to be managed according to the above study visit scheme.

#### Monitoring elevated creatine kinase

Participants with CK>10x ULN plus unexplained muscle pain or weakness are considered to have myopathy (by definition) and should be instructed to stop all study medication (i.e. anacetrapib or placebo plus atorvastatin) permanently. Serum creatinine should also be recorded.

Otherwise, if CK>3x “reference” level (defined as the lower of (i) laboratory ULN and (ii) result at the Randomization visit) study treatment should be continued and an Early Recall visit conducted in about 1 week. At the next visit, CK, ALT and (if CK>10x ULN) creatinine should be recorded. If CK>5x reference with unexplained muscle symptoms or CK>10x reference without such symptoms, then all study medication is to be stopped permanently. Otherwise, if CK >3x reference, a CCO clinician should determine whether study treatment may continue and the timing of future study visits. This decision will be informed by the clinical presentation, including the evolving pattern of CK and ALT, the presence or absence of muscle symptoms, and findings from other investigations.

For participants on study treatment, if CK≤3x reference then the participant may return to routine follow up. If study treatment is restarted following a previous finding of CK>3x reference, then further Early Recall visits will be arranged to monitor CK.

Following any decision to stop study treatment permanently, CK is to be checked at approximately 3-weekly visits (weekly if CK>10x reference) until CK≤3x reference (or it is clear that CK is stable and the risk of myopathy is low). When study treatment is stopped, appropriate advice will be provided to the participant’s managing doctor.

### Modifying study treatment

#### Discontinuation of anacetrapib (or placebo) and LDL-lowering therapy

If adverse events occur that are believed to be due to anacetrapib and/or atorvastatin, or if a significant elevation of liver transaminase or CK develops (see Section 3.5.1), any or all of the study treatments may be permanently or temporarily discontinued. The following events are also sufficient reason to discontinue the study anacetrapib or placebo and/or atorvastatin:

- SAE considered likely to be due to one or more of the study treatments (see Section 2.5.1.3);
- Current use of treatments that are contraindicated with atorvastatin (which still allows the allocated study anacetrapib or placebo to be continued);
- Clear indication or contraindication for anacetrapib (including current use of a non-study treatment that is contraindicated with anacetrapib);
- At the request of the participant or their doctors (for whatever reason) or any other situation where continuing study treatment is not considered to be in the participant’s best interests by their own doctors or the Study clinical team (including child-bearing potential unless using adequate contraception).

If a participant is receiving a contraindicated treatment for a limited period (e.g. a short course of erythromycin or systemic imidazole/triazole antifungal agent), they should stop the relevant study treatment temporarily and restart once the contraindicated treatment has been stopped. CCO clinicians will be available to provide advice on such cases.

Whenever possible, the clinic IT system will prompt LCC Clinic staff to consider whether there are specific reasons for modifying either the randomly-allocated anacetrapib or placebo and/or the study atorvastatin. Participants who have only study atorvastatin stopped may take non-study statin or other lipid-lowering medication (but not a CETP inhibitor) at the discretion of the local investigator and their managing doctor. CCO clinicians will provide advice if required.

#### Requirement for changes to LDL-lowering treatment

Randomized participants who are receiving study atorvastatin and who, in the opinion of their managing doctors, require more or less intensive LDL-lowering therapy may have the dose of study atorvastatin increased (to a maximum of 20 mg daily in Far East, 80 mg daily elsewhere) or reduced. Furthermore, any randomized participant may stop the study atorvastatin and take an LDL-lowering regimen prescribed by their managing doctors. In both circumstances, the randomly-allocated anacetrapib or placebo should generally be continued.

#### Additional monitoring for participants receiving oral anticoagulants

Anticoagulant control with warfarin (or similar drugs) may be disturbed when atorvastatin is started or stopped. For participants who are taking warfarin (or similar drugs), the study IT system will automatically prompt the LCC doctor/nurse to remind the participant of the need for additional monitoring of anticoagulant control if study atorvastatin is altered.

### Unblinding of study treatment

There are 2 main situations in which unblinding of the treatment allocation (anacetrapib or placebo) for an individual participant may be warranted:

- When knowledge of the treatment allocation could materially influence the immediate medical management (e.g. after overdose); and
- When a Clinical Coordinator (or their deputy) reviews a report of a SSAR (see Section 2.5.1.3).

Urgent unblinding is available on a 24-hour basis via the Clinical Trial Service Unit (CTSU) telephone service. Requests for unblinding will be reviewed urgently, and authorized, by the CCO on-call clinician.

### Withdrawal of consent

Participants may decide that they no longer wish to take one or more of the study treatments or are no longer willing to attend study visits. These decisions are not considered to be withdrawals of consent, and appropriate procedures for dealing with them are described elsewhere in this protocol (e.g. for discontinuation of study treatment see Section 3.5.2.1; for alternative methods of follow-up see Section 3.4.4). However, participants are free to withdraw consent for some or all aspects of the study at any time. In order to ensure that relevant safeguards are put in place to maintain the individual’s safety (e.g. if an important safety issue comes to light that might affect a participant who has previously withdrawn from the study) and to prevent a breach of the individual’s decision to withdraw (e.g. to prevent re-invitation of an individual who had previously withdrawn consent), the decision to withdraw should be put in writing and a copy of this should be maintained at the LCC (with key data items being recorded on the study IT system). This written information should specify which aspect(s) of the study consent is being withdrawn: for example, direct contact from study staff; collection of information from a relative or friend; collection of information from non-study doctors or routine data sources; or the storage and analysis of samples for protocol-specified future unspecified assays. (In accordance with FDA guidance, data that have already been collected and incorporated into the study database, including the results of laboratory assays, will continue to be processed.)

## Confirmation and Verification of Study Outcomes

The RCCs will seek additional information only about reports of SAEs that might be importantly relevant to assessment of the efficacy or safety of the study treatment. In general, this will be limited to events initially reported as MI, angina, stroke, coronary or non-coronary revascularization procedure, cancer (excluding non-melanoma skin cancer), myopathy, rhabdomyolysis, non-viral hepatitis, and all deaths. Other events may be added to this list if the Steering Committee consider that this is appropriate in order to ensure a reliable assessment of the clinical effects (particularly safety) of anacetrapib. Relevant information may come from the records held at the LCCs and other hospitals, from participant’s own doctors, from electronic sources and registries (see Section 3.4.5). In some cases (e.g. cancer, blood dyscrasia) it may be necessary to obtain information that pre-dates randomization into the study. The RCC will be responsible for the provisional confirmation of these events, with clinicians based at or overseen by the CCO providing the final adjudication. All review, processing and adjudication of SAEs will be conducted in accordance with the study SOPs and will be blinded to study treatment allocation (anacetrapib or placebo).

1. REFERENCES

1. Cholesterol Treatment Trialists' Collaboration. Efficacy and safety of cholesterol-lowering treatment: prospective meta-analysis of data from 90,056 participants in 14 randomised trials of statins. Lancet. 2005; **366**(9493): 1267-78.

2. Cannon CP, Steinberg BA, Murphy SA, Mega JL, Braunwald E. Meta-analysis of cardiovascular outcomes trials comparing intensive versus moderate statin therapy. Journal of the American College of Cardiology. 2006; **48**(3): 438-45.

3. Pedersen TR, Faergeman O, Kastelein JJP, Olsson AG, Tikkanen MJ, Holme I, et al. High-Dose Atorvastatin vs Usual-Dose Simvastatin for Secondary Prevention After Myocardial Infarction: The IDEAL Study: A Randomized Controlled Trial. JAMA. 2005; **294**(19): 2437-45.

4. LaRosa JC, Grundy SM, Waters DD, Shear C, Barter P, Fruchart J-C, et al. Intensive Lipid Lowering with Atorvastatin in Patients with Stable Coronary Disease. N Engl J Med. 2005; **352**(14): 1425-35.

5. Gordon T, Castelli WP, Hjortland MC, Kannel WB, Dawber TR. High density lipoprotein as a protective factor against coronary heart disease. The Framingham Study. Am J Med. 1977; **62**(5): 707-14.

6. Gordon T, Kannel WB, Castelli WP, Dawber TR. Lipoproteins, cardiovascular disease, and death. The Framingham study. Arch Intern Med. 1981; **141**(9): 1128-31.

7. Gordon DJ, Probstfield JL, Garrison RJ, Neaton JD, Castelli WP, Knoke JD, et al. High-density lipoprotein cholesterol and cardiovascular disease. Four prospective American studies. Circulation. 1989; **79**(1): 8-15.

8. Lewington S, Whitlock G, Clarke R, Sherliker P, Emberson J, Halsey J, et al. Blood cholesterol and vascular mortality by age, sex, and blood pressure: a meta-analysis of individual data from 61 prospective studies with 55,000 vascular deaths. Lancet. 2007; **370**(9602): 1829-39.

9. Parish S, Peto R, Palmer A, Clarke R, Lewington S, Offer A, et al. The joint effects of apolipoprotein B, apolipoprotein A1, LDL cholesterol, and HDL cholesterol on risk: 3510 cases of acute myocardial infarction and 9805 controls. Eur Heart J. 2009; **30**: 2137-46.

10. Di Angelantonio E, Sarwar N, Perry P, Kaptoge S, Ray KK, Thompson A, et al. Major lipids, apolipoproteins, and risk of vascular disease. JAMA. 2009; **302**(18): 1993-2000.

11. Woodward M, Barzi F, Feigin V, Gu D, Huxley R, Nakamura K, et al. Associations between high-density lipoprotein cholesterol and both stroke and coronary heart disease in the Asia Pacific region. Eur Heart J. 2007; **28**(21): 2653-60.

12. Holme I, Aastveit AH, Hammar N, Jungner I, Walldius G. Relationships between lipoprotein components and risk of ischaemic and haemorrhagic stroke in the Apolipoprotein MOrtality RISk study (AMORIS). J Intern Med. 2009; **265**(2): 275-87.

13. Frick MH, Elo O, Haapa K, Heinonen OP, Heinsalmi P, Helo P, et al. Helsinki Heart Study: primary-prevention trial with gemfibrozil in middle-aged men with dyslipidemia. Safety of treatment, changes in risk factors, and incidence of coronary heart disease. N Engl J Med. 1987; **317**(20): 1237-45.

14. Rubins HB, Robins SJ, Collins D, Fye CL, Anderson JW, Elam MB, et al. Gemfibrozil for the secondary prevention of coronary heart disease in men with low levels of high-density lipoprotein cholesterol. Veterans Affairs High-Density Lipoprotein Cholesterol Intervention Trial Study Group. N Engl J Med. 1999; **341**(6): 410-8.

15. Secondary prevention by raising HDL cholesterol and reducing triglycerides in patients with coronary artery disease: the Bezafibrate Infarction Prevention (BIP) study. Circulation. 2000; **102**(1): 21-7.

16. Meade T, Zuhrie R, Cook C, Cooper J. Bezafibrate in men with lower extremity arterial disease: randomised controlled trial. BMJ. 2002; **325**(7373): 1139.

17. The FIELD study investigators. Effects of long-term fenofibrate therapy on cardiovascular events in 9795 people with type 2 diabetes mellitus (the FIELD study): randomised controlled trial. Lancet. **366**(9500): 1849-61.

18. Maccubbin D, Bays HE, Olsson AG, Elinoff V, Elis A, Mitchel Y, et al. Lipid-modifying efficacy and tolerability of extended-release niacin/laropiprant in patients with primary hypercholesterolaemia or mixed dyslipidaemia. Int J Clin Pract. 2008; **62**(12): 1959-70.

19. Clofibrate and niacin in coronary heart disease. JAMA. 1975; **231**(4): 360-81.

20. HPS2-THRIVE: Treatment of High density lipoprotein to Reduce the Incidence of Vascular Events. [cited March 14 2013]; Available from: <http://www.thrivestudy.org/>

21. Thompson A, Di Angelantonio E, Sarwar N, Erqou S, Saleheen D, Dullaart RP, et al. Association of cholesteryl ester transfer protein genotypes with CETP mass and activity, lipid levels, and coronary risk. JAMA. 2008; **299**(23): 2777-88.

22. Bloomfield D, Carlson GL, Sapre A, Tribble D, McKenney JM, Littlejohn TW, 3rd, et al. Efficacy and safety of the cholesteryl ester transfer protein inhibitor anacetrapib as monotherapy and coadministered with atorvastatin in dyslipidemic patients. Am Heart J. 2009; **157**(2): 352-60 e2.

23. Barter PJ, Caulfield M, Eriksson M, Grundy SM, Kastelein JJ, Komajda M, et al. Effects of torcetrapib in patients at high risk for coronary events. N Engl J Med. 2007; **357**(21): 2109-22.

24. Stein EA, Stroes ES, Steiner G, Buckley BM, Capponi AM, Burgess T, et al. Safety and tolerability of dalcetrapib. Am J Cardiol. 2009; **104**(1): 82-91.

25. Kastelein JJ, van Leuven SI, Burgess L, Evans GW, Kuivenhoven JA, Barter PJ, et al. Effect of torcetrapib on carotid atherosclerosis in familial hypercholesterolemia. N Engl J Med. 2007; **356**(16): 1620-30.

26. Bots ML, Visseren FL, Evans GW, Riley WA, Revkin JH, Tegeler CH, et al. Torcetrapib and carotid intima-media thickness in mixed dyslipidaemia (RADIANCE 2 study): a randomised, double-blind trial. Lancet. 2007; **370**(9582): 153-60.

27. Nissen SE, Tardif JC, Nicholls SJ, Revkin JH, Shear CL, Duggan WT, et al. Effect of torcetrapib on the progression of coronary atherosclerosis. N Engl J Med. 2007; **356**(13): 1304-16.

28. Dal-OUTCOMES trial. [cited October 30 2009]; Available from: <http://clinicaltrials.gov/show/NCT00658515>

29. Krishna R, Anderson MS, Bergman AJ, Jin B, Fallon M, Cote J, et al. Effect of the cholesteryl ester transfer protein inhibitor, anacetrapib, on lipoproteins in patients with dyslipidaemia and on 24-h ambulatory blood pressure in healthy individuals: two double-blind, randomised placebo-controlled phase I studies. Lancet. 2007; **370**(9603): 1907-14.

30. Cannon CP, Dansky HM, Davidson M, Gotto AM, Jr., Brinton EA, Gould AL, et al. Design of the DEFINE trial: determining the EFficacy and tolerability of CETP INhibition with AnacEtrapib. Am Heart J. 2009; **158**(4): 513-9 e3.

31. Cholesterol Treatment Trialists' Collaboration. Efficacy and safety of more intensive lowering of LDL cholesterol: a meta-analysis of data from 170,000 participants in 26 randomised trials. Lancet. 2010; **376**(9753): 1670-81.

32. Landray MJ, Haynes R, Hopewell JC, Parish S, Aung T, Tomson J, et al. Effects of extended-release niacin with laropiprant in high-risk patients. N Eng J Med. 2014; **371**(3): 203-12.

33. Schwartz GG, Olsson AG, Abt M, Ballantyne CM, Barter PJ, Brumm J, et al. Effects of dalcetrapib in patients with a recent acute coronary syndrome. N Engl J Med. 2012; **367**(22): 2089-99.

34. Davidson M, Liu SX, Barter P, Brinton EA, Cannon CP, Gotto AM, Jr., et al. Measurement of LDL-C after treatment with the CETP inhibitor anacetrapib. Journal of lipid research. 2013; **54**(2): 467-72.

35. Gotto AM, Jr., Cannon CP, Li XS, Vaidya S, Kher U, Brinton EA, et al. Evaluation of lipids, drug concentration, and safety parameters following cessation of treatment with the cholesteryl ester transfer protein inhibitor anacetrapib in patients with or at high risk for coronary heart disease. Am J Cardiol. 2014; **113**(1): 76-83.

36. Cannon CP, Blazing MA, Giugliano RP, McCagg A, White JA, Theroux P, et al. Ezetimibe Added to Statin Therapy after Acute Coronary Syndromes. N Engl J Med. 2015; **372**(25): 2387-97.

37. ACCELERATE trial press release. 2015 [cited Dec 17th 2015]; Available from: https://investor.lilly.com/releasedetail.cfm?ReleaseID=936130

38. Nicholls SJ, Lincoff AM, Barter PJ, Brewer HB, Fox KAA, Gibson CM, et al. Assessment of the clinical effects of cholesteryl ester transfer protein inhibition with evacetrapib in patients at high-risk for vascular outcomes: Rationale and design of the ACCELERATE trial. Am Heart J. 2015; **170**(6): 1061-9.

39. Pocock SJ, Simon R. Sequential treatment assignment with balancing for prognostic factors in the controlled clinical trial. Biometrics. 1975; **31**(1): 103-15.

40. Peto R, Pike MC, Armitage P, Breslow NE, Cox DR, Howard SV, et al. Design and analysis of randomized clinical trials requiring prolonged observation of each patient. II. analysis and examples. Br J Cancer. 1977; **35**(1): 1-39.

41. Peto R, Pike MC, Armitage P, Breslow NE, Cox DR, Howard SV, et al. Design and analysis of randomized clinical trials requiring prolonged observation of each patient. I. Introduction and design. Br J Cancer. 1976; **34**(6): 585-612.

42. Cuzick J, Edwards R, Segnan N. Adjusting for non-compliance and contamination in randomized clinical trials. Stat Med. 1997; **16**(9): 1017-29.

43. U.S. Department of Health and Human Services Food and Drug Administration. Guidance for Clinical Investigators, Sponsors, and IRBs. Adverse Event Reporting to IRBs - Improving Human Subject Protection. U.S. Department of Health and Human Services, Food and Drug Administration; 2009.

44. Eurpoean Commision Enterpise and industry directorate - General. ENTR/CT 3, Revision 2: Detailed guidance on the collection, verification and presentation of adverse reaction reports arising from clinical trials on medicinal products for human use Brussels; 2006.

45. Holdaas H, Fellstrom B, Cole E, Nyberg G, Olsson AG, Pedersen TR, et al. Long-term cardiac outcomes in renal transplant recipients receiving fluvastatin: the ALERT extension study. Am J Transplant. 2005; **5**(12): 2929-36.

46. Bulbulia R, Bowman L, Wallendszus K, Parish S, Armitage J, Collins R. MRC/BHF Heart Protection Study (HPS): Mortality, Cancer Incidence and Major Vascular Events During 4 Years Post-trial Follow-up of HPS. Journal of the American College of Cardiology. 2007; **49**(9 Supp 1): 393A.

47. Ford I, Murray H, Packard CJ, Shepherd J, Macfarlane PW, Cobbe SM. Long-term follow-up of the West of Scotland Coronary Prevention Study. N Engl J Med. 2007; **357**(15): 1477-86.

1. Appendices

## Appendix 1: Organisational Structure and Responsibilities

**Principal Investigators**

The Principal Investigators have overall responsibility for:

- Design and conduct of the Study
- Preparation of the Protocol and subsequent revisions
- Managing the CCO
- Development of SOPs and computer systems

**Steering Committee**

The Steering Committee is responsible for:

- Agreement of the final Protocol
- Agreeing the Data Analysis Plans
- Reviewing progress of the study and, if necessary, agreeing changes to the Protocol
- Reviewing new studies that may be of relevance
- Review and approval of study reports and sub-study proposals

**Data Monitoring Committee**

The independent Data Monitoring Committee is responsible for:

- Reviewing unblinded interim data according to the schedule outlined in the Protocol
- Advising the Steering Committee if, in their view, the randomized data provide evidence that may warrant early termination for either efficacy or safety (see Section 2.5.2).

**Lipid Monitoring Committee**

The independent Lipid Monitoring Committee is responsible for:

- Reviewing unblinded interim data on blood lipid results
- Advising the Steering Committee if, in their view, the randomized lipid changes are significantly less than anticipated and merit a change to the study design and execution (e.g. modifications to duration of follow-up; see Section 2.4).

**Central Coordinating Office**

The CCO is responsible for the overall coordination of the Study, including:

- Study planning and organisation of Steering Committee meetings
- Agreement of each regional recruitment plan (including countries, number of LCCs, number of participants, and timelines)
- Contractual issues with RCCs and budget administration
- Ensuring necessary regulatory approvals
- Assistance with Ethics Committee applications
- Design, implementation and maintenance of IT systems for the study (including CCO/RCC IT system for administration and clinic IT system for direct data entry)
- Provision of laptops and other study materials to RCCs and LCCs, and provision of IT support to RCCs
- Monitoring of drug supply in liaison with Merck (who will be responsible for drug distribution to each LCC)
- Central laboratory assay and long-term storage of blood and urine samples
- Auditing and monitoring of overall progress of the study
- Clinical safety monitoring, including reporting of SSARS to the Chairman of the Data Monitoring Committee and to Merck (see Section 2.5.1.3)
- Responding to technical, medical and administrative queries from the RCCs
- Management of endpoint adjudication
- Liaison with the Data Monitoring Committee and Merck, and (where appropriate) with regulatory authorities and other outside agencies

**Regional Coordinating Centres**

Each RCC is responsible, under the direction of its Regional Coordinator, for:

- Liaison with regulatory authorities as appropriate
- Identification of potential LCCs and agreement of their recruitment plans (including number of participants and timelines)
- Contractual issues with LCCs and regional budget administration
- Obtaining any central Ethics Committee approval (where appropriate) and assisting LCCs with local Ethics Committee applications
- Training of LCC clinic staff and assistants
- Assisting LCC’s with the identification of suitable individuals
- Distribution of study laptop computers and other study materials to LCCs
- Responding to technical, medical and administrative queries from the LCCs
- Monitoring LCCs through site visits (by the study monitors) and by responding to regular or occasional reports on regional progress prepared by the CCO
- Ensuring appropriate follow-up of abnormal safety blood results
- Ensuring appropriate confirmation of reported events in line with study SOPs (including collection and initial processing of relevant documentation)
- Collection and short-term storage of blood and urine samples from LCCs, and subsequent transport of them to the CCO
- Organisation of meetings of collaborators within the region

**Local Clinical Centres**

The LCC lead investigator and LCC clinic staff are responsible for:

- Obtaining Local Ethics Committee approval (aided by the RCC)
- Obtaining local management approval where necessary
- Provision of adequate clinic space and access to appropriate systems for the identification of potentially eligible individuals
- Conducting clinic procedures; managing and distributing study drugs (in conjunction, if required, with the hospital pharmacy), and maintaining the laptop computer and other study equipment in accordance with the Protocol and SOPs
- Ensuring adequate local laboratory facilities for safety monitoring and, if necessary, processing and temporarily storing samples for central analysis
- Dealing with routine enquiries from participants and their families in collaboration with the RCC
- Obtaining appropriate information when requested to confirm potential primary and secondary study endpoints

## Appendix 2: Visit schedule and procedures

### Clinic procedures

* Registration activities may occur before or during the screening visit and are to be checked at each visit.

** In North America, all non-serious adverse events will be recorded (including those not attributed to study treatment).

*** Remote follow-up may be used for some participants who are unwilling or unable to attend study visits, and for all surviving participants for several years

**** Measured using desktop analyser in the clinic

***** For details of sample collection and storage see section 5.2.2.

### Central laboratory procedures

| **Sample Collection** |  | **Randomization visit** | **2 month visit** | **Annual sample 1*** | **Annual sample 2*** | **Annual sample 3**** | **Annual sample 4*** | **Final visit** |
| --- | --- | --- | --- | --- | --- | --- | --- | --- |
| Pre-specified analysis | Lipid and lipoprotein profile*** | All | All | 5% | 5% | All | 5% | All |
|  | Lipoprotein (a) | All |  | 5% | 5% | All | 5% | >5% |
|  | HbA1c | All |  |  |  |  |  | All |
|  | Creatinine | All |  |  |  |  |  | All |
|  | Albuminuria**** | All |  |  |  |  |  | All |
| Long-term storage | Genetic material | All |  |  |  |  |  |  |
|  | Plasma | All | All | 5% | 5% | All | 5% | All |
|  | Serum | All | All | 5% | 5% | All | 5% | All |
|  | Urine | All |  |  |  |  |  | All |
|  |  |  |  |  |  |  |  |  |
| * Annual samples to be collected from 5% randomized participants annually | | | | | | | | |
| ** Annual sample 3 to be collected from 100% randomized participants when median follow-up is ~2 years | | | | | | | | |
| *** Total, LDL and HDL cholesterol, triglycerides, apolipoproteins A1 and B | | | | | | |  |  |
| **** Urinary albumin:creatinine ratio | | | | | | |  |  |

## Appendix 3: Study Investigators

**STEERING COMMITTEE**

**(Major organisational and policy decisions; blinded to treatment allocation)**

| Chair | Rory Collins |
| --- | --- |
| Deputy Chair | Eugene Braunwald |
| Principal investigators  & Clinical Coordinators | Martin Landray  Louise Bowman |
| Regional representatives | China: Lixin Jiang |
|  | North America: Christopher Cannon, Stephen Wiviott |
|  | United Kingdom: Jane Armitage, Richard Haynes |
|  | Italy: Aldo Maggioni |
|  | Germany: Georg Ertl, Christiane Angermann |
|  | Scandinavia: Terje Pedersen  Japan: Shinya Goto, Tamio Teramoto |
| Health economists | Alastair Gray, Boby Mihaylova |
| Statistician | Jemma Hopewell |
| Other members | Colin Baigent, Philip Barter, Yiping Chen, Zhengming Chen, Jonathan Tobert, Peter Sleight |
| Merck representatives (non-voting) | Robert Blaustein, Paul DeLucca, Yale Mitchel  Gerard van Leijenhorst |

**LIPID MONITORING COMMITTEE**

**(Assessment of unblinded effects on lipid profile)**

| Chair | Chris Granger |
| --- | --- |
| Members | Helen Colhoun |

Statistical programmer providing unblinded analyses: Karl Wallendszus

**DATA MONITORING COMMITTEE**

**(Interim analyses and response to specific concerns)**

| Chair | Peter Sandercock |
| --- | --- |
| Members | David DeMets, John Kjekshus  Andrew Tonkin; James Neuberger |
| Statistician (non-voting) | Jonathan Emberson |

**NATIONAL COORDINATORS**

**(Coordination, legal and regulatory issues in each country)**

| Canada | Jacques Genest |
| --- | --- |
| China | Lixin Jiang |
| Denmark | Kenneth Egstrup |
| Finland | Antero Kesäniemi |
| Germany | Christoph Wanner |
| Italy | Aldo Maggioni |
| Japan | Shinya Goto, Tamio Teramoto |
| Norway | Ottar Nygård |
| Sweden | Lena Jonasson |
| UK | Louise Bowman |
| USA | Christopher Cannon |

**Central Coordinating Office**

Clinical Trial Service Unit (CTSU), Richard Doll Building,

Old Road Campus, Roosevelt Drive, Oxford OX3 7LF, UK

Tel: +44(0)1865 743882, fax: +44 (0)1865 743988

Email: ccoreveal@ctsu.ox.ac.uk

1. It is not anticipated that any CETP inhibitor will be licensed and available for routine use during the recruitment phase of this trial. [↑](#footnote-ref-1)
2. In North America, all non-serious adverse events will be recorded (including those not attributed to study treatment). [↑](#footnote-ref-2)
