# Supplementary material for: Randomized Evaluation of the Effects of Anacetrapib through Lipid-modification (REVEAL)—A large-scale, randomized, placebo-controlled trial of the clinical effects of anacetrapib among people with established vascular disease: Trial design, recruitment, and baseline characteristics
Source: Am Heart J. 2017 May;187:182–90. doi: 10.1016/j.ahj.2017.02.021 (PMC5419667; doi:10.1016/j.ahj.2017.02.021)
Supplement: Supplementary file 4 — REVEAL Data Analysis Plan. [file mmc4.docx]

**Randomized EValuation of the Effects of Anacetrapib through Lipid-modification (HPS3/TIMI55 – REVEAL)**

**Data Analysis Plan**

**EDMS #4298**

**Version 1.0**

**Table of Contents**

[1 Version History 2](#_Toc452624088)

[2 Introduction 3](#_Toc452624089)

[2.1 Background 3](#_Toc452624090)

[3 Roles and Responsibilities 4](#_Toc452624091)

[4 Comparisons of anacetrapib versus placebo 4](#_Toc452624092)

[4.1 Primary assessment 4](#_Toc452624093)

[4.2 Secondary assessments 4](#_Toc452624094)

[4.3 Additional assessments 5](#_Toc452624095)

[4.4 Tertiary assessments 5](#_Toc452624096)

[4.5 Additional safety assessments 7](#_Toc452624097)

[4.6 Analyses of biochemical efficacy 8](#_Toc452624098)

[4.7 Exploratory assessments 9](#_Toc452624099)

[4.8 Health economic assessments 9](#_Toc452624100)

[5 Details of analyses 9](#_Toc452624101)

[5.1 Methods of analysis 9](#_Toc452624102)

[5.2 Allowance for multiplicity of comparisons 10](#_Toc452624103)

[5.3 Tests for heterogeneity of effects 10](#_Toc452624104)

[5.4 Impact of non-compliance with study treatment 11](#_Toc452624105)

[5.5 Analysis of safety and biochemical outcomes 11](#_Toc452624106)

[5.6 Coding and categorization of adverse events 11](#_Toc452624107)

[6 References 12](#_Toc452624108)

# Version History

| 1.0 | Initial version | Created by: Martin Landray, Louise Bowman, Jemma Hopewell  Reviewed by: Merck (May 2016)  Reviewed and approved by: Steering Committee (May 2016)  Released by: Martin Landray (2^nd^ June 2016) |
| --- | --- | --- |

# Introduction

Title: Randomized EValuation of the Effects of Anacetrapib through Lipid-

modification (HPS3 / TIMI55 – REVEAL)

EUDRACT number: 2010-023467-18

ISRCTN number: 48678192

Sponsor: Clinical Trial Service Unit & Epidemiological Studies Unit (CTSU),

University of Oxford

Funder: Merck Sharp & Dohme (Merck)

## Background

This Data Analysis Plan describes the strategy, rationale and statistical methods that will guide assessment of the clinical efficacy and safety of anacetrapib in the REVEAL trial.

REVEAL is a randomized trial investigating the effects of adding the CETP-inhibitor anacetrapib to effective LDL-lowering treatment with atorvastatin. Over 30,000 participants with pre-existing atherosclerotic vascular disease were randomized between anacetrapib 100 mg daily versus matching placebo, with scheduled median follow-up of about 4 years. At the initial Screening visit, eligible individuals were given Run-in medication consisting of placebo anacetrapib and active atorvastatin, and asked to return to the clinic in 8-12 weeks (see Figure). At the Randomization visit, eligible and consenting individuals were randomly allocated anacetrapib 100 mg or matching placebo, along with active atorvastatin at the same dose started at the Screening visit. The primary aim is to assess the effect of anacetrapib on the composite outcome of major coronary event (MCE), defined as coronary death, myocardial infarction or coronary revascularization (see Section 4.1). The key secondary aim is to assess the effect of anacetrapib on coronary death, myocardial infarction or presumed ischaemic stroke (see Section 4.2). Other secondary, tertiary and exploratory assessments (including analyses of safety and biochemical efficacy) are described in Sections 4.2 to 4.7.

***Figure.*** *Outline of randomization and follow-up schedule*

In November 2015 (after approximately 3 years’ median follow-up), the independent Data Monitoring Committee (DMC) considered whether there were reasons to recommend early stopping for efficacy and futility. In summary, for efficacy, the DMC was to consider whether the randomized comparisons in the study provided (i) “proof beyond reasonable doubt” that prolonged use of anacetrapib reduces the primary outcome of major coronary events (with consistent results in key subgroups), as well as coronary death or myocardial infarction, and cardiovascular mortality, with a consistent effect on all-cause mortality; and (ii) evidence that might reasonably be expected to influence materially patient management.

For futility, the DMC was to consider whether continuing the trial was likely to demonstrate any clinically meaningful effects of anacetrapib. For example, a positive result at 4.0 years median follow-up would be unlikely if at 3 years median follow-up and at least 70% of the anticipated total number of primary and secondary events, (i) the hazard ratio for the primary endpoint is greater than 0.95, (ii) there is no evidence of a treatment effect for those patients with >3 years follow-up, and (iii) there are no promising results for any major subgroup (e.g. diabetics, low baseline HDL-cholesterol) or for any major outcome (e.g. coronary death, myocardial infarction or presumed ischaemic stroke). However, the DMC was requested to consider also the possibility that greater benefits might emerge with prolonged follow-up. (For example, the benefits of statin therapy in the first year of treatment are about half those seen in each subsequent year of treatment).

The DMC recommended to the Steering Committee that the trial continue without modification.

# Roles and Responsibilities

All analyses for reports, presentations and publications will be prepared by the coordinating centre at the Clinical Trial Service Unit, University of Oxford (the regulatory sponsor of the REVEAL trial).

# Comparisons of anacetrapib versus placebo

All comparisons will involve comparing outcome during the scheduled treatment period (i.e. from date of randomization to date of final follow-up regardless of whether the participant continues on study treatment or not) among all those participants allocated at randomization to receive anacetrapib 100 mg daily versus all those allocated to receive matching placebo (i.e. “intention-to-treat” analyses).^[[1]](#footnote-1),^^[[2]](#footnote-2)1-3^ Unless otherwise indicated, analyses will be of the first occurrence of the specified outcome. For those events that were subject to adjudication (see Protocol), analyses include all confirmed and unrefuted events.

## Primary assessment

The primary assessment will involve an intention-to-treat comparison among all randomized participants of the effects of allocation to anacetrapib versus placebo on the incidence of major coronary events (defined as the occurrence of coronary death, myocardial infarction or coronary revascularization procedure) during the scheduled treatment period.

## Secondary assessments

Secondary assessments will involve intention-to-treat comparisons among all randomized participants of the effects of allocation to anacetrapib versus placebo during the scheduled treatment period on:

1. Major atherosclerotic events (defined as coronary death, myocardial infarction or presumed ischaemic stroke; the key secondary outcome);
2. Presumed ischaemic stroke (i.e. not known to be haemorrhagic); and
3. Major vascular events (defined as coronary death, myocardial infarction, coronary revascularization or presumed ischaemic stroke).

In addition, each of the individual components of the primary outcome (i.e. coronary death; myocardial infarction; and coronary revascularization) will be tested separately.

## Additional assessments

These assessments (which have been specifically requested prior to the start of the study by regulatory agencies) will involve intention-to-treat comparisons among all randomized participants of the effects of allocation to anacetrapib versus placebo during the scheduled treatment period on:

1. Cardiovascular death or myocardial infarction; and
2. Cardiovascular death, myocardial infarction or stroke.

## Tertiary assessments

Tertiary assessments will involve intention-to-treat analyses among all randomized participants of the effects of allocation to anacetrapib versus placebo during the scheduled treatment period on:

1. Coronary death or myocardial infarction, and, separately, myocardial infarction alone
2. Mortality
   - from all causes combined; and, separately, within particular categories of causes, i.e.:
     - all cardiovascular causes combined; and, separately:
   - coronary (including sudden cardiac death)
   - other cardiac
   - stroke
   - other vascular
     - all non-cardiovascular causes combined; and, separately:
   - cancer
   - infection
   - respiratory
   - hepatic
   - other medical*
   - non-medical

* including undetermined cause

1. Stroke
   - of any type combined; and, separately, of particular types, i.e.:
   - confirmed ischaemic stroke
   - confirmed haemorrhagic stroke
   - stroke of unknown/unconfirmed aetiology
2. Major coronary events, major atherosclerotic events, and major vascular events, separately, in various subdivisions:
   1. occurring more than one year after randomization;
   2. disease type prior to randomization:
      - coronary heart disease
      - cerebrovascular disease
      - peripheral arterial disease
      - diabetes*

and timing of most recent qualifying vascular event: <12; ≥12 months

* diabetes at randomization is defined as self-reported diabetes recorded on screening or randomization form; or diabetes-related adverse event recorded on or before date of randomization; or use of hypoglycaemic medication reported on randomization form

- 1. three similar-sized groups based on lipid and lipoprotein measurements* from the Randomization visit:
     - HDL cholesterol (mmol/L): <0.9;≥0.9<1.1;≥1.1
     - LDL cholesterol (mmol/L): <1.4;≥1.4<1.7;≥1.7
     - total cholesterol (mmol/L): <3.2: ≥3.2<3.7; ≥3.7
     - non-HDL cholesterol (mmol/L): <2.2; ≥2.2<2.6; ≥2.6
     - triglycerides (mmol/L): <1.2; ≥1.2<1.7; ≥1.7
     - apolipoprotein B (mg/dL): <60; ≥60<70; ≥70
     - apolipoprotein A1 (mg/dL) <110; ≥110<125; ≥125
     - lipoprotein (a) (nmol/L): <15; ≥15<55; ≥55

* using results measured in the central laboratory

- 1. various other categories of participant based on their Randomization visit values:
     - age (years): <65; ≥65<70; ≥70
     - sex: male; female
     - region: North America; Europe; Asia
     - blood pressure (mmHg):
  - systolic <125;≥125<140;≥140
  - diastolic <75;≥75<85;≥85
    - kidney function
  - estimated Glomerular Filtration Rate (ml/min/1.73m^2^) derived using the CKD-EPI equation^4^: <60; ≥60
  - urinary albumin:creatinine ratio (mg/mmol): normo-albuminuria (<3); micro-albuminuria (≥3 <30); macro-albuminuria (≥30)
    - alcohol intake: current drinker; former/never drinker
    - cigarette smoking: current; former; never
    - body mass index (kg/m^2^): <25; ≥25<30; ≥30
    - waist:hip ratio: low (<0.87 in women; <0.94 in men); medium (≥0.87<0.93 in women; ≥0.94<1.00 in men); high (≥0.93 in women; ≥1.00 in men)
    - history of heart failure: yes; no
    - atorvastatin dose (mg): low (10 in China; 20 in rest of the world); high (20 in China; 80 in rest of the world)
  1. presence and absence of other treatments used at the Randomization visit:
     - angiotensin-converting-enzyme inhibitors or angiotensin-receptor blockers:
     - aspirin or other antiplatelet drugs
     - diuretics
     - calcium-channel blockers
     - beta-blockers

1. Urgent and non-urgent coronary revascularization, considered separately and combined;
2. Non-coronary revascularizations, including percutaneous interventions (with or without stenting), surgical revascularization procedures (e.g. grafting, endarterectomy), and amputation for presumed vascular disease;
3. Hospitalization for heart failure;
4. Development of diabetes mellitus* among those not known to be diabetic at randomization (see section 4.4.iv.b);

* development of diabetes is defined as a post-randomization diabetes-related adverse event; or use of hypoglycaemic medication reported on at least one follow-up form

1. Combination of first and subsequent occurrences of the primary outcome;
2. Cancer (fatal or non-fatal combined, and excluding any known to pre-date randomization and non-melanoma skin cancers)
   - at all sites combined; and, separately, at particular sites
     - gastrointestinal
     - respiratory
     - breast
     - melanoma
     - genitourinary
     - haematological
     - other or not specified
3. Serious adverse events (overall and by subtype) due to:
   - Infection at all sites combined; and, separately, at particular sites:
     - Respiratory
     - Renal and urinary
     - Gastrointestinal
     - Skin
     - Systemic (including septicaemia)
     - Other or unspecified
   - Respiratory disease of all types combined; and, separately, of particular types:
     - Bronchial
     - Pulmonary vascular
     - Pleural
     - Other lower respiratory tract
     - Upper respiratory tract
     - Other respiratory

## Additional safety assessments

Additional safety assessments will include intention-to-treat analyses among all randomized participants of the effects of allocation to anacetrapib versus placebo during the scheduled treatment period on:

1. blood pressure-related outcomes:
   - blood pressure at 12 months and at final follow-up visit

- mean level of systolic blood pressure (SBP) and diastolic blood pressure (DBP)
- level of SBP (<140; ≥140<160; ≥160<180; ≥180 mmHg) and level of DBP (<90; ≥90<100; ≥100<110; ≥110 mmHg)
- change from randomization in SBP and DBP (in each case <-10; ≥-10<0; ≥0<10; ≥10 mmHg)
  - serious adverse events due to hypertension

1. muscle-related outcomes:
   - creatine kinase [CK] >5x and ≤10x laboratory upper limit of normal [ULN] plus ALT >1.5x ULN
   - CK >10x and ≤40x ULN overall and, separately, with muscle symptoms
   - CK >40x ULN overall and, separately, with evidence of end-organ damage, such as doubling of serum creatinine (i.e. rhabdomyolysis^[[3]](#footnote-3)^)
2. liver-related outcomes:
   - ALT >3x ULN plus bilirubin >2x ULN, with CK ≤5x ULN
   - ALT >3x ULN on 2 occasions within about one week (i.e. 1 to 14 days), with CK ≤5x ULN
   - liver injury (i.e. ALT >3x ULN plus bilirubin >2x ULN; ALT >10x ULN [regardless of bilirubin level]; liver transplantation; or death due to liver disease) by cause*

* causes to be classified as: known (e.g. infection; alcohol; cancer; drug-related [excluding randomized anacetrapib/placebo]); and unknown (including cases believed due to study treatment with no alternative cause identified)

1. renal function at final visit
   - estimated glomerular filtration rate (eGFR)
     - difference in mean eGFR
     - development of impaired renal function (i.e. eGFR <60ml/min/1.73m2) among those with normal renal function (i.e. eGFR ≥60ml/min/1.73m2) at randomization.
   - albuminuria
     - development of albuminuria (urinary albumin:creatinine ratio [uACR] ≥3 mg/mmol) in those with normo-albuminuria at randomization
     - development of macro-albuminuria in those with normo- or micro-albuminuria at randomization.
2. discontinuation of study treatment* overall and, separately, by various causes
   - serious adverse events**
   - non-serious adverse events**
   - other reasons (various categories; e.g. cannot attend clinic, participant concern, medical advice, administrative reason)

* reason recorded at time of latest discontinuation (without any subsequent restart)

** categorised by assigned System Organ Class (see section 5.6)

## Analyses of biochemical efficacy

Biochemical efficacy of anacetrapib 100 mg daily will be assessed in non-fasting specimens taken from all participants at the randomization visit, at the 2 month follow-up visit, at a follow-up visit when median follow-up is about 2 years and at the final study visit. In addition, samples will be taken annually in 5% of participants. The following biochemical efficacy outcomes will be measured using standard automated assays on all samples:

- total cholesterol
- HDL cholesterol
- LDL cholesterol
- non-HDL cholesterol
- triglycerides
- apolipoprotein A1
- apolipoprotein B

In addition, lipoprotein (a) is to be measured in all participants at randomization and at about 2 years after the median participant is randomized, and in at least 5% of participants annually. Samples of genetic material, plasma, serum, and urine have been biobanked for possible future analyses.

The effect of anacetrapib on other aspects of lipid and lipoprotein profile (such as lipoprotein particle size) may also be examined. In particular, a sub-study is comparing the assessment of LDL cholesterol using a direct method (which is used routinely for REVEAL samples) with a beta quantification method among around 2000 samples taken at 2 years of follow-up. The results of this sub-study may inform the interpretation of the main analyses of the effect of anacetrapib on LDL cholesterol concentration.

## Exploratory assessments

Exploratory assessments will be made of other possible beneficial or adverse effects of anacetrapib during the scheduled treatment period. Examples include all serious adverse events and all non-serious adverse reactions, both overall and categorised by assigned System Organ Class (see section 5.6). Other analyses will include disturbances of cardiac rhythm (atrial fibrillation/flutter; ventricular tachycardia/fibrillation), mood (assessed using the Mental Health Inventory [MHI]-5 questionnaire), and cognitive function (assessed using the Modified Telephone Interview for Cognitive Status [TICS-M] questionnaire). Additional exploratory analyses may be undertaken, as considered appropriate, and cautiously interpreted (see section 5.2 for approaches to handling multiple hypothesis testing). These may include exploration of the effects of anacetrapib on additional clinical outcomes over time (e.g. ≤1 year vs >1 year post-randomization) or in particular sub-groups of participant (e.g. high HDL-cholesterol or low LDL-cholesterol at randomization). The results of the REVEAL trial will also be considered in the context of information from randomized clinical trials of the effects of other lipid-modifying drugs (e.g. statins, ezetimibe) on cardiovascular events. In addition, a sub-study is assessing the concentration of anacetrapib in serial measures of blood and adipose tissue in around 500 individuals. The results of this sub-study may inform the interpretation of the analyses of possible beneficial or adverse effects of anacetrapib on clinical outcomes.

## Health economic assessments

Appropriate health economic assessments regarding the use of anacetrapib among patients at risk of vascular events will be conducted. Analyses of EQ5D questionnaires recorded at randomization and final follow-up visits will be used to determine the effects of clinical outcomes on quality of life. The detailed analysis plan for health economic assessments is outside the remit of this document.

# Details of analyses

## Methods of analysis

All participants randomized to anacetrapib will be compared with all participants randomized to placebo, regardless of whether a participant received all, some or none of their allocated treatment (i.e. intention-to-treat [ITT] analyses).^2,3^ A participant may contribute to more than one assessment if they have events of more than one type (e.g. non-fatal ischaemic stroke followed by coronary death). For the time-to-event analyses, survival analytic methods will be used to evaluate the time to the first event during the entire study period. For each outcome, log-rank method will be used to estimate the average event rate ratio comparing all those allocated active anacetrapib with all those allocated placebo.^3^ Estimates of the event rate ratio will be shown with 95% confidence intervals. Kaplan-Meier estimates for the time to each of the primary and secondary outcomes will also be plotted (with their associated log-rank p-values). Cox regression may be used where rate ratios are extreme (e.g. >2 or <0.5). In all analyses, two-sided p-values (2P) <0.05 will be considered statistically significant (after any allowance for multiplicity as outlined in section 5.2). Recurrent events will be analysed using the negative binomial and sensitivity analyses will be performed using alternative methods such as the Andersen-Gill approach.

At the time of finalizing the Data Analysis Plan in May 2016, around 99% of the 28,613 surviving randomized participants had attended their most recent scheduled visit. Since loss-to-follow-up at study end is anticipated to be minimal (<1%), no exploratory analyses investigating the impact of missing data not at random/informative missingness are planned.

## Allowance for multiplicity of comparisons

The primary outcome will be assessed without adjustment for multiplicity. If a significant difference is demonstrated, then the key secondary outcome (i.e. major atherosclerotic events) and each of the components of the primary outcome (coronary death, myocardial infarction, and coronary revascularization) will be tested without adjustment. If a significant difference is demonstrated in the key secondary outcome, then presumed ischaemic stroke will be assessed. The remaining secondary outcome of major vascular events and the two additional composite outcomes requested by regulatory agencies (section 4.3) will be assessed without adjustment for multiplicity.

If there is directional consistency in the effect of the treatment on the primary outcome and on presumed ischaemic stroke, emphasis will be placed on the subgroup analyses for the secondary outcome of major vascular event (which incorporates both outcomes).

For tertiary, additional pre-specified and exploratory analyses (as noted in section 4.7), allowance in their interpretation will be made for multiple hypothesis testing,^2,3^ taking into account the nature of events (including timing, duration and severity) and evidence from other studies. In addition to the pre-specified comparisons, many other analyses will be performed with due allowance for their exploratory and, perhaps, data-dependent nature. Conventionally, two-sided P-values <0.05 are often described as “significant”. But, the larger the number of events on which a comparison is based and the more extreme the P-value after any allowance has been made for the nature of the particular comparison (i.e. primary, secondary or tertiary; pre-specified or exploratory), the more reliable the comparison and, hence, the more definite any finding will be considered. Analyses of fatal events will be interpreted in the light of the observed effects on relevant non-fatal events.^3^

## Tests for heterogeneity of effects

Tests for heterogeneity of the proportional effect observed in subgroups will be used (whilst controlling the false discovery rate to allow for multiple comparisons) to determine whether the proportional effects in specific subcategories are clearly different from the overall effect.^2,3^ If, however, patient categories can be arranged in some meaningful order (e.g. age at randomization: <65; ≥65<70; ≥70) then assessment of any trend will be made. Unless otherwise stated, those with missing values of baseline values will be included in the subgroup that includes the median (for continuous variables) or the largest group (for categorical variables), and the number of missing values will be clearly indicated.

When a number of different subgroups are considered, chance alone may lead to there being no apparent effect in several subgroups in which the effect of treatment really is about the same as is observed overall. In such circumstances, “lack of direct evidence of benefit” is not good “evidence of lack of benefit”, and clearly significant overall results would provide strong indirect evidence of benefit in some small subgroups where the results, considered in isolation, are not conventionally significant (or, even, perhaps, slightly adverse).^2,3,5^  Hence, unless the proportional effect in some specific subcategory is clearly different from that observed overall, the effect in that subcategory is likely to be best estimated indirectly by applying the proportional effect observed among all patients in the trial to the absolute risk of the event observed among control patients in that category.^5^

## Impact of non-compliance with study treatment

Based on the observed differences in cholesterol during follow-up between all those allocated active anacetrapib and all those allocated placebo (i.e. irrespective of compliance), cholesterol-weighted analyses will be used to estimate the effects of actual compliance with anacetrapib on the primary and secondary outcomes overall and in different circumstances.^6^ The effect of full compliance with anacetrapib will also be estimated based on the observed intention-to-treat effects on the primary and secondary outcomes of allocation to anacetrapib and the average in-trial compliance with the randomized treatment (determined by participant reports and treatment issue records).

## Analysis of safety and biochemical outcomes

For each of the events listed as additional safety outcomes (section 4.5), the number of randomized participants with at least 1 event will be compared using standard tests for differences in proportions. For analyses of continuous variables, differences in means between the randomized groups will be assessed (unless otherwise specified). Exploratory analyses will be conducted among particular categories of participant. For participants selected for blood sampling who were alive at the time of the scheduled follow-up assessment but failed to provide a sample, LDL- and HDL-cholesterol values will be imputed based on baseline and 2 month values while taking into account their background atorvastatin and reported compliance.

## Coding and categorization of adverse events

All adverse events are coded using Medical Dictionary for Regulatory Activities (MedDRA) version 14.0. Unless otherwise stated, events are to be categorised according to System Organ Class (SOC), with priority given to cancer and infection as follows: If the preferred term (PT) maps to the Cancer SOC, then it is assigned to this SOC, regardless of whether this is its primary SOC. Otherwise, if the PT is linked to the Infection SOC, then it is assigned to this SOC. Otherwise (if it is not in cancer or infection) then the PT is assigned to its MedDRA Primary SOC.

# References

1. Peto R, Peto J. Asymptotically efficient invariant test procedures. J Roy Stat Soc 1972;135(2):185-207.

2. Peto R, Pike MC, Armitage P, et al. Design and analysis of randomized clinical trials requiring prolonged observation of each patient. I. Introduction and design. Br J Cancer 1976;34:585-612.

3. Peto R, Pike MC, Armitage P, et al. Design and analysis of randomized clinical trials requiring prolonged observation of each patient. II. analysis and examples. Br J Cancer 1977;35:1-39.

4. Levey AS, Stevens LA, Schmid CH, et al. A new equation to estimate glomerular filtration rate. Ann Intern Med 2009;150:604-12.

5. Collins R, MacMahon S. Reliable assessment of the effects of treatment on mortality and major morbidity, I: clinical trials. Lancet 2001;357:373-80.

6. Cuzick J, Edwards R, Segnan N. Adjusting for non-compliance and contamination in randomized clinical trials. Stat Med. 1997;16(9):1017-29.

1. Following investigation of a serious breach of good clinical practice at one centre (centre id 3314), the Steering Committee determined (blind to knowledge of any unblinded results) at its meeting in February 2014 that all data from this centre should be excluded from all analyses. [↑](#footnote-ref-1)
2. A single censoring date is to be used in the REVEAL analyses for both fatal and non-fatal events. The censoring date is the earliest date of: (1) A valid death report (fatal serious adverse event); (2) A final follow-up conducted in person or by telephone to the participant, relative or carer; (3) For anyone without a final follow-up conducted in person or by telephone to participant, relative or carer the approximate end of the final follow-up window i.e. 31^st^ January 2017; or (4) The date of withdrawal of consent. [↑](#footnote-ref-2)
3. For the purposes of these analyses, rhabdomyolysis is defined as CK>40x ULN with muscle symptoms or CK>10x ULN with evidence of end-organ damage such as doubling of serum creatinine [↑](#footnote-ref-3)
